# Supplementary material for: Concentrations, Number of Doses, and Formulations of Aluminium Adjuvants in Vaccines: A Systematic Review with Meta-Analysis and Trial Sequential Analysis of Randomized Clinical Trials
Source: Vaccines (Basel). 2023 Nov 27;11(12):1763. doi: 10.3390/vaccines11121763 (PMC10871092; doi:10.3390/vaccines11121763)
Supplement: Supplementary file 1 [file vaccines-11-01763-s001.zip › vaccines-2689401-supplementary.pdf]

SUPPLEMENTAL MATERIAL

This supplemental material has been provided by the authors to give readers additional information about their work.

Supplement to: Marija Barbateskovic, Sarah Louise Klingenberg SL, Sara Russo Krauss S, De Zhao Kong, Zhang Tong Wu, Sesilije B Petersen, Mette Kenfelt, Christian Gluud. Concentrations, number of doses, and formulations of aluminium adjuvants in vaccines. A systematic review with meta-analysis and Trial Sequential Analysis of randomized clinical trials.

Correspondence: Professor Christian Gluud: christian.gluud@ctu.dk; Phone: +45 3545 7175

Supplemental material for

Concentration, number of doses and formulation of aluminium adjuvants in vaccines. A systematic review with meta-analysis and Trial Sequential Analysis

SEARCH STRATEGIES **3**
PRISMA FLOWCHART **10**
TRIALS AWAITING CLASSIFICATION **11**
EXCLUDED TRIALS **18**
RISK OF BIAS SUMMARY **19**
CHARACTERISTICS OF INCLUDED TRIALS **20**
ADDITIONAL INFORMATION AND ANALYSES ON THE OUTCOMES **38**
Comparison 1 – Higher vs lower aluminium concentration **38**
Individual serious adverse events 38
Individual adverse events 39
Serological response 59
Comparison 2 – Higher versus lower number of doses of aluminium adjuvant **60**
All-cause mortality 60
Serious adverse events 61
Individual serious adverse events 62
Adverse events 63
Adverse events – Trial Sequential Analysis 63
Individual adverse events 64
Serological response 65
Comparison 3 – Aluminium phosphate versus aluminium hydroxide **66**
All-cause mortality 66
Serious adverse events 66
Adverse events 67
Adverse events – Trial Sequential Analysis 67
Individual adverse events 68
Serological response 68
DIFFERENCES BETWEEN PROTOCOL AND REVIEW **69**
REFERENCES **70**

SEARCH STRATEGIES

Cochrane Central Register of Controlled Trials (2023, Issue 1) in the Cochrane Library (3862 hits)

#1	MeSH descriptor: [Adjuvants, Immunologic] explode all trees
#2	MeSH descriptor: [Vaccine Excipients] explode all trees
#3	((immunologic* or alum* or vaccine*) and (adjuvan* or excipient*))
#4	#1 or #2 or #3
#5	MeSH descriptor: [Vaccines] explode all trees
#6	MeSH descriptor: [Vaccination] explode all trees
#7	(vaccin* or immuni?ation* or innocula*)
#8	(biothrax or DT* or sanofi or daptacel or infanrix or kinrix or quadracel or pediarix or pentacel or vaxelis or pedvax or havrix or vaqta or engerix or recombivax or twinrix or gardasil or ixiaro or bexsero or trumenba or prevnar or tenivac or tdvax or adacel or boostrix)
#9	#5 or #6 or #7 or #8
#10	#4 and #9

MEDLINE Ovid (1946 to 20 January 2023) (4727 hits)

1.	exp Adjuvants, Immunologic/
2.	exp Vaccine Excipients/
3.	((immunologic* or alum* or vaccine*) and (adjuvan* or excipient*)).mp. [mp=title, book title, abstract, original title, name of substance word, subject heading word, floating sub-heading word, keyword heading word, organism supplementary concept word, protocol supplementary concept word, rare disease supplementary concept word, unique identifier, synonyms]
4.	1 or 2 or 3
5.	exp Vaccines/
6.	exp Vaccination/
7.	(vaccin* or immuni?ation* or innocula*).mp. [mp=title, book title, abstract, original title, name of substance word, subject heading word, floating sub-heading word, keyword heading word, organism supplementary concept word, protocol supplementary concept word, rare disease supplementary concept word, unique identifier, synonyms]
8.	(biothrax or DT* or sanofi or daptacel or infanrix or kinrix or quadracel or pediarix or pentacel or vaxelis or pedvax or havrix or vaqta or engerix or recombivax or twinrix or gardasil or ixiaro or bexsero or trumenba or prevnar or tenivac or tdvax or adacel or boostrix).mp. [mp=title, book title, abstract, original title, name of substance word, subject heading word, floating sub-heading word, keyword heading word, organism

supplementary concept word, protocol supplementary concept word, rare disease supplementary concept word, unique identifier, synonyms]

9. 5 or 6 or 7 or 8

10. 4 and 9

11. randomized controlled trial.pt.

12. controlled clinical trial.pt.

13. clinical trials as topic.sh.

14. (random* or placebo*).ab. or trial.ti.

15. 11 or 12 or 13 or 14

16. exp animals/ not humans.sh.

17. 15 not 16

18. 10 and 17

Embase Ovid (1974 to 20 January 2023) (7208 hits)

1. exp immunological adjuvant/

2. exp vaccine excipient/

3. ((immunologic* or alum* or vaccine*) and (adjuvan* or excipient*)).mp. [mp=title, abstract, heading word, drug trade name, original title, device manufacturer, drug manufacturer, device trade name, keyword heading word, floating subheading word, candidate term word]

4. 1 or 2 or 3

5. exp vaccine/

6. exp Vaccination/

7. (vaccin* or immuni?ation* or innocula*).mp. [mp=title, abstract, heading word, drug trade name, original title, device manufacturer, drug manufacturer, device trade name, keyword heading word, floating subheading word, candidate term word]

8. (biothrax or DT* or sanofi or daptacel or infanrix or kinrix or quadracel or pediarix or pentacel or vaxelis or pedvax or havrix or vaqta or engerix or recombivax or twinrix or gardasil or ixiaro or bexsero or trumenba or prevnar or tenivac or tdvax or adacel or boostrix).mp. [mp=title, abstract, heading word, drug trade name, original title, device manufacturer, drug manufacturer, device trade name, keyword heading word, floating subheading word, candidate term word]

9. 5 or 6 or 7 or 8

10. 4 and 9

11. Randomized controlled trial/ or Controlled clinical study/ or randomization/ or intermethod comparison/ or double blind procedure/ or human experiment/ or retracted article/

12. (random$ or placebo or parallel group$1 or crossover or cross over or assigned or allocated or volunteer or volunteers).ti,ab.

13. (compare or compared or comparison or trial).ti.

14. ((evaluated or evaluate or evaluating or assessed or assess) and (compare or compared or comparing or comparison)).ab.

15. (open adj label).ti,ab.

16. ((double or single or doubly or singly) adj (blind or blinded or blindly)).ti,ab.

17. ((assign$ or match or matched or allocation) adj5 (alternate or group$1 or intervention$1 or patient$1 or subject$1 or participant$1)).ti,ab.

18. (controlled adj7 (study or design or trial)).ti,ab.

19. (erratum or tombstone).pt. or yes.ne.

20. or/11-19

21. (random$ adj sampl$ adj7 ('cross section$' or questionnaire$ or survey$ or database$1)).ti,ab. not (comparative study/ or controlled study/ or randomi?ed controlled.ti,ab. or randomly assigned.ti,ab.)

22. Cross-sectional study/ not (randomized controlled trial/ or controlled clinical study/ or controlled study/ or randomi?ed controlled.ti,ab. or control group$1.ti,ab.)

23. (((case adj control$) and random$) not randomi?ed controlled).ti,ab.

24. (Systematic review not (trial or study)).ti.

25. (nonrandom$ not random$).ti,ab.

26. 'Random field$'.ti,ab.

27. (random cluster adj3 sampl$).ti,ab.

28. (review.ab. and review.pt.) not trial.ti.

29. 'we searched'.ab. and (review.ti. or review.pt.)

30. 'update review'.ab.

31. (databases adj4 searched).ab.

32. (rat or rats or mouse or mice or swine or porcine or murine or sheep or lambs or pigs or piglets or rabbit or rabbits or cat or cats or dog or dogs or cattle or bovine or monkey or monkeys or trout or marmoset$1).ti. and animal experiment/

33. Animal experiment/ not (human experiment/ or human/)

34. or/21-33

35. 20 not 34

36. 10 and 35

LILACS (VHL Regional Portal; 1982 to 20 January 2023) (396 hits)
(((immunologic* OR alum* OR vaccine*) AND (adjuvan* OR excipient*)) AND (vaccin* OR immunesation* OR immunization* OR innocula* OR biothrax OR dt* OR sanofi OR daptacel OR infanrix OR kinrix OR quadracel OR pediarix OR pentacel OR vaxelis OR pedvax OR havrix OR vaqta OR engerix OR recombivax OR twinrix OR gardasil OR ixiaro OR bexsero OR trumenba OR prevnar OR tenivac OR tdvax OR adacel OR boostrix)) AND (db:("LILACS"))

BIOSIS (Web of Science) (1969 to 20 January 2023) (1950 hits)

#5	#4 AND #3
#4	TI=(random* or blind* or placebo* or meta-analys* or trial*) OR TS=(random* or blind* or placebo* or meta-analys*)
#3	#2 AND #1
#2	TS=(vaccin* or immunisation* or immunization* or innocula* or biothrax or sanofi or daptacel or infanrix or kinrix or quadracel or pediarix or pentacel or vaxelis or pedvax or havrix or vaqta or engerix or recombivax or twinrix or gardasil or ixiaro or bexsero or trumenba or prevnar or tenivac or tdvax or adacel or boostrix)
#1	TS=((immunologic* or alum* or vaccine*) and (adjuvan* or excipient*))

Science Citation Index Expanded (1900 to 20 January 2023) and Conference Proceedings Citation Index – Science (1990 to 20 January 2023) (Web of Science) (3304 hits)

#5	#4 AND #3
#4	TI=(random* or blind* or placebo* or meta-analys* or trial*) OR TS=(random* or blind* or placebo* or meta-analys*)
#3	#2 AND #1
#2	TS=(vaccin* or immunisation* or immunization* or innocula* or biothrax or sanofi or daptacel or infanrix or kinrix or quadracel or pediarix or pentacel or vaxelis or pedvax or havrix or vaqta or engerix or recombivax or twinrix or gardasil or ixiaro or bexsero or trumenba or prevnar or tenivac or tdvax or adacel or boostrix)
#1	TS=((immunologic* or alum* or vaccine*) and (adjuvan* or excipient*))

Chinese Biomedical Literature Database (CBM) (1860 to 18 March 2023) (0 hits)

#1	adjuvants OR immunologic [Common fields: precise]
#2	vaccine excipients [Common fields: precise]
#3	#1 OR #2
#4	immunologic OR alum OR vaccine [Common fields: precise]
#5	adjuvan OR excipient [Common fields: precise]
#6	#4 AND #5
#7	#3 OR #6
#8	vaccines OR vaccination [Common fields:precise]
#9	vaccin* OR Immunilation* OR Innocula* [Common fields: precise]
#10	(biothrax vaccine OR DT vaccine * OR sanofi vaccine OR daptacel vaccine OR infanrix vaccine OR kinrix vaccine OR quadracel vaccine OR pediarix vaccine OR pentacel vaccine OR vaxelis vaccine OR pedvax vaccine OR havrix vaccine OR vaqta vaccine OR engerix vaccine OR recombivax vaccine OR twinrix vaccine OR gardasil vaccine OR ixiaro vaccine OR bexsero vaccine OR trumenba vaccine OR prevnar

	vaccine OR tenivac vaccine OR tdvax vaccine OR adacel vaccine OR boostrix vaccine) [Common fields: precise]
#11	#8 OR #9 OR #10
#12	#7 AND #11
#13	randomized controlled trial [Common fields: precise]
#14	controlled clinical trial [Common fields: precise]
#15	random* OR placebo* [Absrtact: precise]
#16	trial [Title: precise]
#17	#13 OR #14 OR #15 OR #16
#18	animals NOT humans [Common fields: precise]
#19	#17 NOT #18
#20	#12 AND #19

The search was also performed in Chinese.

China Network Knowledge Information (CNKI 1979 to 18 March 2023) (0 hits)

#1	'adjuvants' + ' immunologic' [TKA]
#2	'vaccine excipients' [TKA]
#3	' immunologic' + 'alum' + 'vaccine' [TKA]
#4	' adjuvant' + 'excipient' [TKA]
#5	#3 AND #4
#6	#1 OR #2 OR #5
#7	Vaccines [TKA]
#8	Vaccination [TKA]
#9	'vaccin' + 'immunilation ' + ' innocula' [Title or keyword]
#10	'biothrax vaccine' OR 'DT vaccine' OR 'sanofi vaccine' OR 'daptacel vaccine' OR 'infanrix vaccine' OR 'kinrix vaccine' OR 'quadracel vaccine' OR 'pediarix vaccine' OR 'pentacel vaccine' OR 'vaxelis vaccine' OR 'pedvax vaccine' OR 'havrix vaccine' OR 'vaqta vaccine' OR 'engerix vaccine' OR 'recombivax vaccine' OR 'twinrix vaccine' OR 'gardasil vaccine' OR 'ixiaro vaccine' OR 'bexsero vaccine' OR 'trumenba vaccine' OR 'prevnar vaccine' OR 'tenivac vaccine' OR 'tdvax vaccine' OR 'adacel vaccine' OR 'boostrix vaccine' [Title or keyword]
#11	#7 OR #8 OR #9 OR #10
#12	#6 AND #11
#13	randomized controlled trial [Full text]
#14	controlled clinical trial [Full text]

#15	clinical trials [TKA]
#16	'random' OR 'placebo' [Abstract]
#17	trial [Title]
#18	#13 OR #14 OR #15 OR #16 OR #17
#19	'animals' NOT 'humans' [TKA]
#20	#18 NOT #19
#21	#12 AND #20

The search was also performed in Chinese.

Chinese Science Journal Database (VIP 1989 to 18 March 2023) (0 hits)

#1	'adjuvants' OR ' immunologic' [Title or keyword]
#2	'vaccine excipients' [Title or keyword]
#3	' immunologic' OR 'alum' OR 'vaccine' [Title or keyword]
#4	' adjuvant' OR 'excipient' [Title or keyword]
#5	#3 AND #4
#6	#1 OR #2 OR #5
#7	Vaccines [Title or keyword]
#8	Vaccination [Title or keyword]
#9	'vaccin' OR 'immunilation ' OR ' innocula' [Title or keyword]
#10	'biothrax vaccine' OR 'DT vaccine' OR 'sanofi vaccine' OR 'daptacel vaccine' OR 'infanrix vaccine' OR 'kinrix vaccine' OR 'quadracel vaccine' OR 'pediarix vaccine' OR 'pentacel vaccine' OR 'vaxelis vaccine' OR 'pedvax vaccine' OR 'havrix vaccine' OR 'vaqta vaccine' OR 'engerix vaccine' OR 'recombivax vaccine' OR 'twinrix vaccine' OR 'gardasil vaccine' OR 'ixiaro vaccine' OR 'bexsero vaccine' OR 'trumenba vaccine' OR 'prevnar vaccine' OR 'tenivac vaccine' OR 'tdvax vaccine' OR 'adacel vaccine' OR 'boostrix vaccine' [Title or keyword]
#11	#7 OR #8 OR #9 OR #10
#12	#6 AND #11
#13	randomized controlled trial [Title or keyword]
#14	controlled clinical trial [Title or keyword]
#15	clinical trials [Title or keyword]
#16	'random' OR 'placebo' [Abstract]
#17	trial [Title]
#18	#13 OR #14 OR #15 OR #16 OR #17
#19	'animals' NOT 'humans' [Title or keyword]

#20	#18 NOT #19
#21	#12 AND #20

The search was also performed in Chinese.

Wanfang Database (1990 to 18 March 2023) (0 hits)

#1	'adjuvants' OR ' immunologic' [Title or keyword]
#2	'vaccine excipients' [Title or keyword]
#3	' immunologic' OR 'alum' OR 'vaccine' [Title or keyword]
#4	' adjuvant' OR 'excipient' [Title or keyword]
#5	#3 AND #4
#6	#1 OR #2 OR #5
#7	Vaccines [Title or keyword]
#8	Vaccination [Title or keyword]
#9	'vaccin' OR 'immunilation ' OR ' innocula' [Title or keyword]
#10	'biothrax vaccine' OR 'DT vaccine' OR 'sanofi vaccine' OR 'daptacel vaccine' OR 'infanrix vaccine' OR 'kinrix vaccine' OR 'quadracel vaccine' OR 'pediarix vaccine' OR 'pentacel vaccine' OR 'vaxelis vaccine' OR 'pedvax vaccine' OR 'havrix vaccine' OR 'vaqta vaccine' OR 'engerix vaccine' OR 'recombivax vaccine' OR 'twinrix vaccine' OR 'gardasil vaccine' OR 'ixiaro vaccine' OR 'bexsero vaccine' OR 'trumenba vaccine' OR 'prevnar vaccine' OR 'tenivac vaccine' OR 'tdvax vaccine' OR 'adacel vaccine' OR 'boostrix vaccine' [Title or keyword]
#11	#7 OR #8 OR #9 OR #10
#12	#6 AND #11
#13	randomized controlled trial [Title or keyword]
#14	controlled clinical trial [Title or keyword]
#15	clinical trials [Title or keyword]
#16	'random' OR 'placebo' [Abstract]
#17	trial [Title]
#18	#13 OR #14 OR #15 OR #16 OR #17
#19	'animals' NOT 'humans' [Title or keyword]
#20	#18 NOT #19
#21	#12 AND #20

The search was also performed in Chinese.

PRISMA FLOWCHART

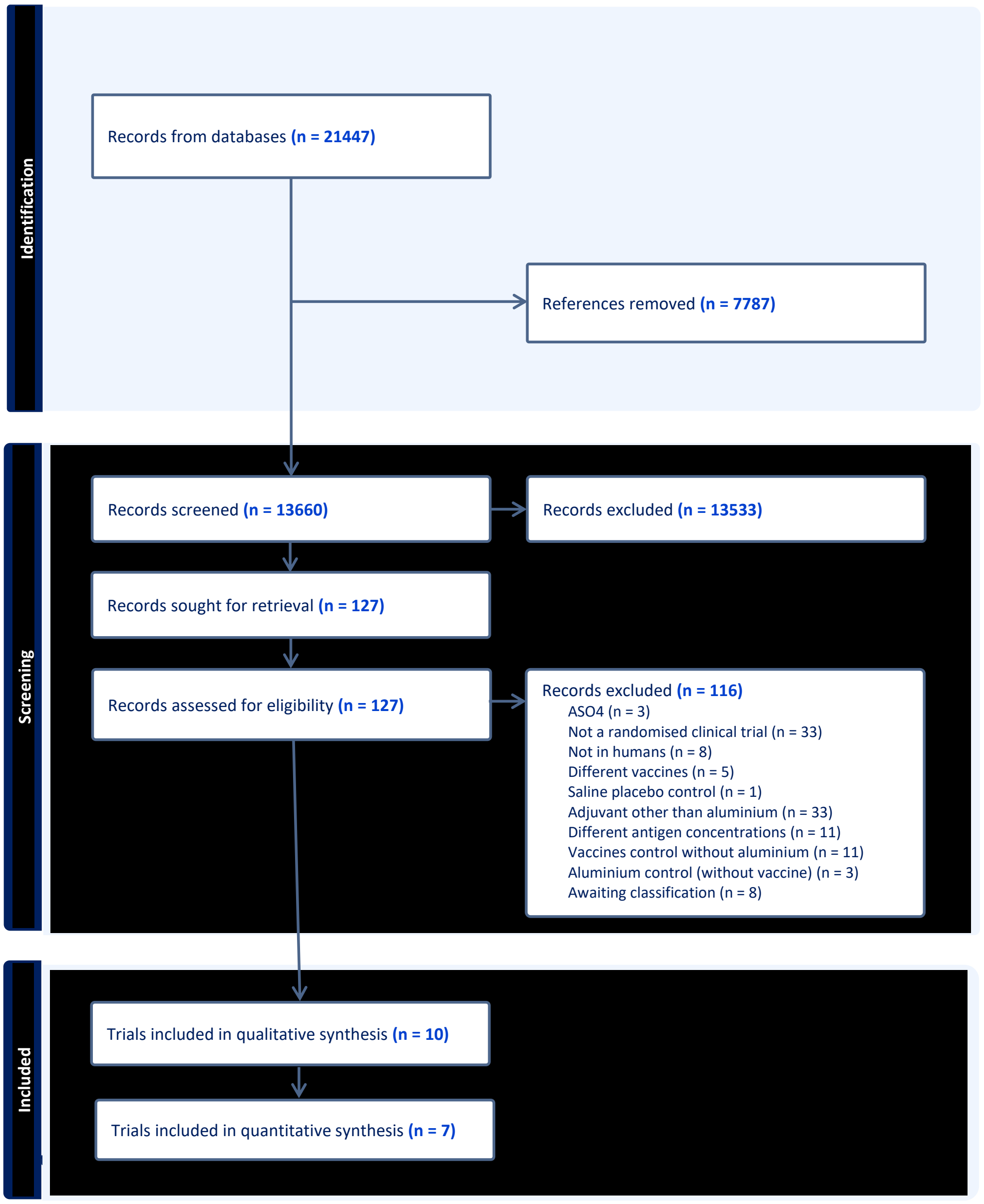

TRIALS AWAITING CLASSIFICATION

1 NCT00000835. A multicenter, randomized, placebo-controlled, double-blind trial to evaluate the safety and immunogenicity of an HIV-1 pseudovirion vaccine

clinicaltrials.gov/show/NCT00000835 (First posted 31 August 2001)

Methods Multicenter, randomised, placebo-controlled, double-blind trial
Participants

- Sex: all
- Age: adults
- Ethnicity: not reported
- Diagnosis: not reported
- Inclusion criteria: negative ELISA for HIV-1 antibody within 8 weeks of initial immunization, CD4 count >= 400 cells/mm3, negative Hepatitis B surface antigen, normal history and physical examination
- Exclusion criteria: medical or psychiatric condition or occupational responsibilities preclude compliance with the protocol, present psychosis, active syphilis, active tuberculosis, Hepatitis B antigenemia, history of immunodeficiency, chronic illness, malignancy, or autoimmune disease, cancer unless there has been surgical excision followed by sufficient observation period to give a reasonable assurance of cure, suicide attempts, recent suicidal ideation or who have past psychosis, anaphylaxis or other serious adverse reactions to vaccines, serious allergic reaction to any substance, requiring hospitalization or emergent medical care (e.g. Stevens-Johnson syndrome, bronchospasm, or hypotension), prior receipt of HIV-1 vaccines or placebo recipient in a previous HIV vaccine trial, use of experimental agents within 30 days prior to study, live attenuated vaccines within 60 days of study, medically indicated subunit or killed vaccines (e.g. influenza, pneumococcal) within 2 weeks prior to study, receipt of blood products or immunoglobulin in the past 6 months, volunteers having identifiable higher risk behavior for HIV infection as determined by screening questions designed to identify risk factors for HIV infection

Interventions

- Vaccine type: HIV-1 Pseudovirion Vaccine
- Aluminium concentration: not reported
- Aluminium type: alum
- Aluminium manufacturer: not reported
- Vaccination schedule: intramuscularly at 0, 1, 3, 6, and 12 months

Outcomes not reported

2 NCT00100724. Trial of rPA-102 Vaccine in Healthy Adult Volunteers

clinicaltrials.gov/ct2/show/NCT00100724 (First posted 6 January 2005)

Methods Phase 2 Multi-Center, Randomized Dose-Finding Trial

Participants

- Sex: all
- Age: 18-55 years
- Ethnicity: not reported
- Diagnosis: healthy
- Inclusion criteria: able to understand the study and give written informed consent. Healthy male or female aged 18-55 years old (inclusive) without significant physical or clinical laboratory abnormalities. Two intact upper arms with sufficient intramuscular (IM) tissue in the deltoid region for vaccine administration. For females, negative serum pregnancy test at screening and agreement to use adequate birth control during the first 2 months of the study. Willingness and ability to return for all follow-up visits and blood draws for the duration of the

study. Willingness to complete the Volunteer Diary and to report concomitant medications and adverse events to the study site monitors during the study period.

- Exclusion criteria: prior history of, or known exposure to any form of B. anthracis or any anthrax immunization. Member of the Armed Services (Active Duty or Reserve) since 1990, with history of previous anthrax vaccination. Employment in an industry involved in contact with ruminant animals, veterinary sciences, or other exposure to B. anthracis, or emergency first responders. Expected to be noncompliant with study visits or planning to move within 12 months. Body mass index of >35 or <19. Known allergy to aluminum hydroxide, kanamycin, or any other aminoglycoside antibiotics (such as gentamicin). Pregnancy (positive urine pregnancy test within 24 hours prior to vaccination), or lactation. HIV positive (by history or screening ELISA). Hepatitis B or C positive (by history or screening HBsAg/anti-HCV ELISA). Active or past internal organ, hematologic malignancy, or metastatic cutaneous malignancy. History of, or current autoimmune disease, including but not limited to systemic lupus erythematosus, scleroderma, and polyarteritis. Immunodeficiency or unstable medical condition as determined by baseline medical history, physical exam, and laboratory assessment. Received or plans to receive licensed live vaccines within 30 days of study vaccination. Received or plans to receive licensed killed vaccines within 14 days of study vaccination. Received or plans to receive immunoglobulin or other blood products within 60 days of study vaccination. Received or plans to receive experimental drugs/vaccines within 30 days prior to, and for the duration of the study. Received or plans to receive systemic immunosuppressive therapy, radiation therapy, or high-dose inhaled steroids within 30 days of study vaccination. Use of systemic chemotherapy within 5 years prior to study. History of Guillain-Barre Syndrome. In addition to the conditions listed above, the following may qualify as a reason to exclude a volunteer from the study: fever along with moderate or serious illness within 3 days of vaccination or any condition that, in the opinion of the investigator, would render vaccination unsafe or would interfere with the study evaluations. Pending resolution of these symptoms, a volunteer may be reconsidered for vaccination.

Interventions
- Vaccine type: rPA102 vaccine
- Aluminium concentration: not reported
- Aluminium type: not reported
- Aluminium manufacturer: not reported
- Vaccination schedule: not reported

Outcomes
- Safety and immunogenicity

**3**	**NCT00359619. Human papillomavirus vaccine immunogenicity and safety trial in young adult women with gsk biologicals novel hpv vaccine**<br><br>**clinicaltrials.gov/show/NCT00359619 (First posted 2 August 2006)**

Methods Randomised, parallel assignment, open label, prevention

Participants
- Sex: female
- Age: adult
- Ethnicity: not reported
- Diagnosis: healthy
- Inclusion criteria: a female who enrolled in the study 102115 and received three doses of vaccine. Written informed consent obtained from the subject prior to enrolment.
- Exclusion criteria: use (or planned use during the study period) of any investigational or non-registered product or off-label use of licensed product (drug or vaccine), chronic administration of immunosuppressants or other immune-modifying drugs occurring less than three months

prior to blood sampling, administration of immunoglobulins and/or any blood products within the three months preceding blood sampling, planned administration of any HPV vaccine, other than that foreseen by the study protocol, during the study period.

Interventions

- Vaccine type: HPV vaccine
- Aluminium concentration: not reported
- Aluminium type: not reported
- Aluminium manufacturer: not reported
- Vaccination schedule: intramuscularly at 0, 1, and 6 months

Outcomes

- Immunogenicity: number of seroconverted subjects against human papillomavirus-16 antibodies; geometric mean titers for human papillomavirus-16 antibodies; number of seroconverted subjects against human papillomavirus-18 antibodies; geometric mean titers for human papillomavirus-18 antibodies.

4 NCT00479648. A phase 2 study of immunogenicity, safety and tolerability of CSL412 in elderly participants

clinicaltrials.gov/show/NCT00479648 (First posted 28 May 2007).

Methods Phase II, double-blind, randomised, controlled, multi-centre trial

Participants

- Sex: all
- Age: adults
- Ethnicity: not reported
- Diagnosis: not reported
- Inclusion criteria: aged ≥ 18 to ≤ 45 OR ≥ 60, ability to provide pre-vaccination venous blood sample
- Exclusion criteria: history of clinically significant medical conditions, immunomodulative therapy, acute infection

Interventions

- Vaccine type: an adjuvanted influenza vaccine
- Aluminium concentration: not reported
- Aluminium type: not reported
- Aluminium manufacturer: not reported
- Vaccination schedule: not reported

Outcomes

- seroprotection rate, HI titre & seroconversion/ significant increase
- grade 3 or higher, vaccine associated fever or vaccine associated site ulceration, abscess or necrosis

5 NCT00952276. A study of different formulations of an adjuvanted a/h1n1 pandemic vaccine in healthy adults and the elderly

clinicaltrials.gov/show/NCT00952276 (First posted 6 August 2009).

Methods Randomized, parallel assignment

Participants

- Sex: all

- Age: adults
- Ethnicity: not reported
- Diagnosis: healthy
- Inclusion criteria: healthy adults aged 18 years or older on the day of inclusion, informed consent has been signed and dated, able to attend all scheduled visits and comply with all trial procedures, for a woman of child-bearing potential, use of an effective method of contraception or abstinence from at least 4 weeks prior to the first vaccination, until at least 4 weeks after last vaccination
- Exclusion criteria: known pregnancy or positive urine pregnancy test, currently breastfeeding a child, participation in another clinical trial investigating a vaccine, drug, medical device, or a medical procedure in the 4 weeks preceding the first trial vaccination, planned participation in another clinical trial during the present trial period, receipt of any vaccine in the 4 weeks preceding the trial vaccination, except for the inactivated seasonal influenza vaccine, within two weeks preceding trial vaccination, planned receipt of any vaccine prior to the Day 42 blood sample, receipt of blood or blood-derived products in the past 3 months which might interfere with the assessment of immune response. Known or suspected congenital or acquired immunodeficiency, immunosuppressive therapy such as anti-cancer chemotherapy or radiation therapy within the preceding 6 months, or long-term systemic corticosteroid therapy (prednisone or equivalent for more than 2 consecutive weeks within the past 3 months), self-reported seropositivity for Human Immunodeficiency Virus (HIV), Hepatitis B antigen, or Hepatitis C, known systemic hypersensitivity to any of the vaccine components, or history of a life-threatening reaction to the vaccine(s) used in the trial or to a vaccine containing any of the same substances, self reported thrombocytopenia contraindicating intramuscular (IM) vaccination, bleeding disorder or receipt of anticoagulants in the 3 weeks preceding inclusion contraindicating IM vaccination, deprived of freedom by an administrative or court order, or in an emergency setting, or hospitalized involuntarily, current alcohol abuse or drug addiction that may interfere with the subject's ability to comply with trial procedures, chronic illness that in the opinion of the Investigator, is at a stage where it might interfere with trial conduct or completion, employees of the Investigator or study center, with direct involvement in the proposed study or other studies under the direction of that Investigator or study center, as well as family members of the employees or the Investigator, previous participation in a swine-origin A/H1N1 pandemic flu trial except if performed in 1976, any confirmed case of influenza (including swine-origin A/H1N1 Influenza) since March 2009, febrile illness (temperature ≥ 100.4°F [≥ 38.0°C]) or moderate or severe acute illness/infection (according to Investigator judgment) on the day of vaccination, personal or family history of Guillain-Barré syndrome, active neoplastic disease or a history of any hematologic malignancy, known seizure/epilepsy history and/or taking anti-seizure medication, receipt of psychiatric drugs. Subjects receiving a single antidepressant drug and stable for at least 3 months prior to enrollment, without decompensating symptoms will be allowed to enroll in the study, any Grade 1, 2, or 3 liver function values (Alanine aminotransferase (ALT), Aspartate aminotransferase (AST), and Alkaline phosphatase) observed in blood sample taken at screening, any Grade 2 or Grade 3 laboratory abnormalities in blood sample taken at screening, receipt of any monovalent 2009 pandemic H1N1 vaccine

Interventions

- Vaccine type: influenza vaccine
- Aluminium concentration: not reported
- Aluminium type: not reported
- Aluminium manufacturer: not reported
- Vaccination schedule: participants will receive a single injection of their randomised vaccine on Day 0

Outcomes

- Number of Participants With Detectable Antibodies Before and Following Vaccination With Either Adjuvanted or Non-adjuvanted A/H1N1 Pandemic Vaccine or a Placebo: Age 18 to 64 Years [Time Frame: Day 0 and Day 21 post-vaccination]
- Number of Participants With Seroprotection Before and Following Vaccination With Either Adjuvanted or Non-adjuvanted A/H1N1 Pandemic Vaccine or a Placebo: Age 18 to 64 Years [Time Frame: Day 0 and Day 21 post-vaccination]
- Geometric Mean Titers (GMTs) of A/H1N1 Antibodies Before and Following Vaccination With Either Adjuvanted or Non-adjuvanted A/H1N1 Pandemic Vaccine or a Placebo: Age 18 to 64 Years [Time Frame: Day 0 and Day 21 post-vaccination]
- Number of Participants With Detectable Antibodies Before and Following Vaccination With Either Adjuvanted or Non-adjuvanted A/H1N1 Pandemic Vaccine or a Placebo: Age ≥ 65 Years [Time Frame: Day 0 and Day 21 post-vaccination]
- Number of Participants With Seroprotection Before and Following Vaccination With Either Adjuvanted or Non-adjuvanted A/H1N1 Pandemic Vaccine or a Placebo: Age ≥ 65 Years [Time Frame: Day 0 and Day 21 post-vaccination]
- Geometric Mean Titers (GMTs) of A/H1N1 Antibodies Before and Following Vaccination With Either Adjuvanted or Non-adjuvanted A/H1N1 Pandemic Vaccine or a Placebo: Age ≥ 65 Years [Time Frame: Day 0 and Day 21 post-vaccination]
- Number of Participants Reporting a Solicited Injection Site or Systemic Reaction Following Vaccination With Either Adjuvanted or Non-adjuvanted A/H1N1 Pandemic Vaccine or a Placebo: Age 18 to 64 Years [Time Frame: Day 0 up to Day 7 post-vaccination]
- Number of Participants Reporting a Solicited Injection Site or Systemic Reaction Following Vaccination With Either Adjuvanted or Non-adjuvanted A/H1N1 Pandemic Vaccine or a Placebo: Age ≥ 65 Years [Time Frame: Day 0 up to Day 7 post-vaccination]

6 NCT01594320. A/H5N1 virus-like particle antigen dose ranging study with adjuvant 1

clinicaltrials.gov/show/NCT01594320 (First posted 9 May 2012)

Methods Randomized, parallel assignment

Participants

- **Sex:** all
- **Age:** adult
- **Ethnicity:** not reported
- **Diagnosis:** healthy
- **Inclusion criteria:** healthy adult male or female, ≥18 and ≤49 years of age, willing and able to give informed consent prior to study enrollment, able to comply with study requirements, and women must have a negative urine pregnancy test prior to each vaccination; will be advised through the Informed Consent process to avoid becoming pregnant over the duration of the study, and must assert that they will employ an effective form of birth control for the duration of the study. Acceptable forms of birth control are: credible history of continuous abstinence from heterosexual activity or prior surgical sterilization, hormonal contraceptives (oral, injectable, implant, patch, ring), double-barrier contraceptives (condom or diaphragm, with spermicide), and intrauterine device (IUD).
- **Exclusion criteria:** any ongoing, symptomatic acute or chronic illness requiring medical or surgical care. Asymptomatic conditions (e.g. hypertension, dyslipidemia) that are being managed medically and that are not associated with evidence of end-organ damage are not exclusionary provided they are clinically stable (defined as no unscheduled medical interventions or change in medications for cause within 3 months), any history of jaundice, or of hepatic injury due to drug (prescription, OTC, or illicit) or alcohol use or viral hepatitis; or the presence of hepatitis B surface antigen or hepatitis C antibody at screening, any grade 1 or higher (as based on the Toxicity Grading Scale [TGS]) abnormality in ALT, AST, alkaline phosphatase or total bilirubin levels, any grade 2 or higher (as based on the TGS) vital sign or clinical laboratory abnormality not specified in criterion 3 above. Note that any abnormal vital

sign may be repeated at the Investigator's discretion. Participation in research involving investigational product (drug / biologic / device) within 45 days before planned date of first vaccination. History of a serious reaction to prior influenza vaccination, history of Guillain-Barré Syndrome (GBS) within 6 weeks following a previous influenza vaccine, received any vaccine in the 4 weeks preceding the study vaccination; or any A/H5N1 avian influenza vaccine at any time. Any known or suspected immunosuppressive condition, acquired or congenital, including HIV infection as determined by history and/or physical examination, chronic administration (defined as more than 14 continuous days) of immunosuppressants or other immune-modifying drugs within 6 months prior to the administration of the study vaccine. An immunosuppressant dose of glucocorticoid will be defined as a systemic dose ≥10 mg of prednisone per day or equivalent. The use of topical, inhaled, and nasal glucocorticoids will be permitted, administration of immunoglobulins and/or any blood products within the 3 months preceding the administration of the study vaccine or during the study. Acute disease at the time of enrollment (defined as the presence of a moderate or severe illness with or without fever, or an oral temperature >38.0°C on the planned day of vaccine administration), known disturbance of coagulation, women who are pregnant or breastfeeding, or plan to become pregnant during the study, suspicion or recent history (within one year of planned vaccination) of alcohol or other substance abuse, any condition that in the opinion of the investigator would pose a health risk to the subject if enrolled or could interfere with evaluation of the vaccine or interpretation of study results (including neurologic or psychiatric conditions deemed likely to impair the quality of safety reporting)

Interventions

- Vaccine type: avian flu vaccine
- Aluminium concentration: not reported
- Aluminium type: not reported
- Aluminium manufacturer: not reported
- Vaccination schedule: two doses of the assigned test article on Study Days 0 and 21

Outcomes

- Number of solicited and unsolicited adverse events in H5N1 VLP antigen dose groups delivering HA with/without Adjuvant 1. [Time Frame: Day 42]
- Measurement of HAI antibody seroconversion rates and GMT achieved by a constant H5N1 VLP antigen dose alone and in combination with Adjuvant 1. [Time Frame: Day 42]

7	**NCT01596725. A/H5N1 virus-like particle antigen dose ranging study with adjuvant 2**<br><br>**clinicaltrials.gov/show/NCT01596725 (First posted 11 May 2012)**

Methods Phase 1 randomized, observer-blinded, dose-ranging trial

Participants

- **Sex:** all
- **Age:** adult
- **Ethnicity:** not reported
- **Diagnosis:** healthy
- **Inclusion criteria:** healthy adult male or female, ≥18 and ≤49 years of age, willing and able to give informed consent prior to study enrollment, able to comply with study requirements, and women must have a negative urine pregnancy test prior to each vaccination; will be advised through the Informed Consent process to avoid becoming pregnant over the duration of the study, and must assert that they will employ an effective form of birth control for the duration of the study. Acceptable forms of birth control are: credible history of continuous abstinence from heterosexual activity or prior surgical sterilization, hormonal contraceptives (oral, injectable, implant, patch, ring), double-barrier contraceptives (condom or diaphragm, with spermicide), and intrauterine device (IUD).

- **Exclusion criteria:** any ongoing, symptomatic acute or chronic illness requiring medical or surgical care. Asymptomatic conditions (e.g. hypertension, dyslipidemia) that are being managed medically and that are not associated with evidence of end-organ damage are not exclusionary provided they are clinically stable (defined as no unscheduled medical interventions or change in medications for cause within 3 months), any history of jaundice, or of hepatic injury due to drug (prescription, OTC, or illicit) or alcohol use or viral hepatitis; or the presence of hepatitis B surface antigen or hepatitis C antibody at screening, any grade 1 or higher (as based on the Toxicity Grading Scale [TGS]) abnormality in ALT, AST, alkaline phosphatase or total bilirubin levels, any grade 2 or higher (as based on the TGS) vital sign or clinical laboratory abnormality not specified in criterion 3 above. Note that any abnormal vital sign may be repeated at the Investigator's discretion. Participation in research involving investigational product (drug / biologic / device) within 45 days before planned date of first vaccination. History of a serious reaction to prior influenza vaccination, history of Guillain-Barré Syndrome (GBS) within 6 weeks following a previous influenza vaccine, received any vaccine in the 4 weeks preceding the study vaccination; or any A/H5N1 avian influenza vaccine at any time. Any known or suspected immunosuppressive condition, acquired or congenital, including HIV infection as determined by history and/or physical examination, chronic administration (defined as more than 14 continuous days) of immunosuppressants or other immune-modifying drugs within 6 months prior to the administration of the study vaccine. An immunosuppressant dose of glucocorticoid will be defined as a systemic dose ≥10 mg of prednisone per day or equivalent. The use of topical, inhaled, and nasal glucocorticoids will be permitted, administration of immunoglobulins and/or any blood products within the 3 months preceding the administration of the study vaccine or during the study. Acute disease at the time of enrollment (defined as the presence of a moderate or severe illness with or without fever, or an oral temperature >38.0°C on the planned day of vaccine administration), known disturbance of coagulation, women who are pregnant or breastfeeding, or plan to become pregnant during the study, suspicion or recent history (within one year of planned vaccination) of alcohol or other substance abuse, any condition that in the opinion of the investigator would pose a health risk to the subject if enrolled or could interfere with evaluation of the vaccine or interpretation of study results (including neurologic or psychiatric conditions deemed likely to impair the quality of safety reporting)

Interventions
- Vaccine type: avian flu vaccine
- Aluminium concentration: not reported
- Aluminium type: not reported
- Aluminium manufacturer: not reported
- Vaccination schedule: two doses of the assigned test article on Study Days 0 and 21

Outcomes
- Number of solicited and unsolicited adverse events in H5N1 VLP antigen dose groups delivering HA with/without Adjuvant 2. [Time Frame: Day 42]
- Measurement of HAI antibody seroconversion rates and GMT achieved by a constant H5N1 VLP antigen dose alone and in combination with Adjuvant 2. [Time Frame: Day 42]

8	**Edson, P.J. Uso do bioterápico preparado a partir do precipitado da vacina tríplice na prevenção dos efeitos adversos locais produzidos pela adjuvante hidróxido de alumínio existente no precipitado da vacina. Pesqui. homeopática 2000;16(2):27-42.**

Not able to retrieve full text

Methods Not reported in abstract
Participants Not reported in abstract
Intervention Not reported in abstract
Outcomes Not reported in abstract

EXCLUDED TRIALS

Trials not meeting the inclusion criteria

(D'Arcy Hart et al. 1957; Meiklejohn 1960; Azurin et al. 1967; Weibel et al. 1967; Woolridge et al. 1967; Anonymous 1969; Fox et al. 1970; Bechelli et al. 1971; Wigand et al. 1971; Comstock and Edwards 1972; Amato Neto et al. 1973; Anonymous 1973; Ajjan and Triau 1975; Rosenbaum et al. 1976; Kuwert et al. 1978; Collier et al. 1979; Egerton et al. 1979; Sarateanu and Ehrengut 1981b, 1981a; Baxendale and Lutticken 1982; Brown AE et al. 1982; Brugh et al. 1983; Kanai et al. 1985; Frasch and Zahradnik 1986; Herrington et al. 1987; Bystryn et al. 1988; Rutter et al. 1988; Keefer et al. 1994; Timms et al. 1994; Dowlati et al. 1996; Coulaud et al. 1997; Roy and Venugopalan 1998; Baldo et al. 1999; Drachenberg et al. 1999; Muderspach Laila et al. 1999; Nct 1999; Pereira Junior 2000; Anonymous 2001b, 2001c, 2001a; Fernandez-Cruz et al. 2001; Gotch and Imami 2001; Nardin et al. 2001; Gardner Jason et al. 2004; Slovin et al. 2004; Kong et al. 2005; Nct 2005b, 2005a; Authier and Gherardi 2006; Knuf et al. 2006; Nct 2006b, 2006a; Bankhead 2007; Lell et al. 2007; Nct 2007b, 2007a; Weitz et al. 2007; Gao et al. 2008; Keam and Harper 2008; Langley et al. 2008; Nct 2008; Evidence-based review of clinical studies on trauma 2009; Anonymous 2009; GlaxoSmithKline Vaccine HPV-007 Study Group 2009; Nct 2009c, 2009d, 2009a, 2009b; Van Buynder et al. 2010; Loebermann et al. 2011; Meier et al. 2011; Di Mario et al. 2012; Kulkarni et al. 2012; Nct 2012b, 2012a; Riedmann 2012; Fries et al. 2013; Brown J et al. 2014; De Serres 2014; Kreijtz et al. 2014; Nagaputra et al. 2014; Nct 2014; Leroux-Roels, Maes, et al. 2016; Leroux-Roels, Marchant, et al. 2016; Leroux-Roels, Van Damme, et al. 2016; Martins et al. 2016; Anonymous 2017b, 2017a; Burny et al. 2017; Leroux-Roels et al. 2017; Nct 2017; Sacks 2017; Barouch et al. 2018; Chichester et al. 2018; Joob and Wiwanitkit 2018; Kooijman et al. 2018; Sullivan et al. 2018; Tomaka et al. 2018; Schmader et al. 2019; Tomaka et al. 2019; Actrn 2020; Arif et al. 2020; Diaz et al. 2020; Moser et al. 2020; Nct 2020; Nielsen et al. 2020; Vink et al. 2020; Xia et al. 2020; Aslam et al. 2021; Bulbul et al. 2021; Ma H et al. 2021; Ma KSK et al. 2021; Ma R et al. 2021; McFarland et al. 2021; Mustafa 2021; Ratnapriya et al. 2021; Vajo et al. 2021; Abbasi 2022; Diemert et al. 2022; Dingermann 2022; Dunkle et al. 2022; Houser et al. 2022; Pactr 2022; Raponi et al. 2022; Siegmund-Schultze 2022; Zhang L et al. 2022; Zhang NR et al. 2022; Zhang Y, Ma X, et al. 2022; Zhang Y, Wang Y, et al. 2022).

RISK OF BIAS SUMMARY

	Random sequence generation (selection bias)	Allocation concealment (selection bias)	Blinding of participants and personnel (performance bias): All outcomes	Blinding of outcome assessment (detection bias): Safety	Blinding of outcome assessment (detection bias): Immunogenicity	Incomplete outcome data (attrition bias): Safety	Incomplete outcome data (attrition bias): Immunogenicity	Selective reporting (reporting bias)	Other bias
August 2017	?	?	?	?	?	+	−	+	+
de Kleijn 2000	?	+	+	?	?	+	+	?	+
Goubau 1992	?	?	?	?	?	?	+	−	+
Leroux-Roels 2013	+	+	?	?	?	+	+	?	+
Murphy 1983	?	?	+	?	+	?	?	?	+
NCT00562237	?	?	?	?	?	?	?	?	?
Rupp 2019	?	?	?	?	?	+	−	+	+
Schwameis 2016	+	+	+	+	+	?	?	+	+
Theeten 2005	?	?	?	+	+	+	+	−	+
Villa 2006	?	?	+	+	+	+	+	?	?

Risk of bias summary: review authors' judgements about each risk of bias item for each included trial

CHARACTERISTICS OF INCLUDED TRIALS

August 2017	
Methods	Phase 2 randomised, observer-blind, placebo-controlled, dose-ranging trial
Participants	**Sex:** females<br>**Age:** adults<br>**Ethnicity:** mixed<br>**Diagnosis:** healthy<br>**Inclusion criteria:** "healthy adult females, ≥ 18 and ≤ 35 years of age. "Healthy" shall be defined by the absence of any illness, acute or chronic, that requires ongoing systemic therapy for the control of symptoms or prevention of disability. Willing and able to give informed consent prior to study enrolment. Able to comply with study requirements. Women who are not surgically sterile must have a negative urine pregnancy test prior to each vaccination; will be advised through the Informed Consent process to avoid becoming pregnant over the duration of the study, and must assert that they will employ an effective form of birth control for the duration of the study. Acceptable forms of birth control are: credible history of continuous abstinence from heterosexual activity, hormonal contraceptives (oral, injectable, implant, patch, ring), double-barrier contraceptives (condom or diaphragm, with spermicide), and IUD".<br>**Exclusion criteria:** "participation in research involving investigational product (drug / biologic / device) within 45 days before planned date of first vaccination. History of a serious reaction to any prior vaccination. Received any vaccine in the 4 weeks preceding the study vaccination; or any RSV vaccine at any time. Any known or suspected immunosuppressive condition, acquired or congenital, as determined by history and/or physical examination. Chronic administration (defined as more than 14 continuous days) of immunosuppressants or other immune-modifying drugs within 6 months prior to the administration of the study vaccine. An immunosuppressant dose of glucocorticoid will be defined as a systemic dose ≥10mg of prednisone per day or equivalent. The use of topical, inhaled, and nasal glucocorticoids will be permitted. Administration of immunoglobulins and/or any blood products within the 3 months preceding the administration of the study vaccine or during the study. Donated blood within 3 weeks of the planned date of first vaccination. Acute disease at the time of enrolment (defined as the presence of a moderate or severe illness with or without fever, or an oral temperature > 38.0°C on the planned day of vaccine administration). Known disturbance of coagulation. Women who are pregnant or breastfeeding, or plan to become pregnant during the study. Suspicion or recent history (within one year of planned vaccination) of alcohol or other substance abuse. Any condition that in the opinion of the Investigator would pose a health risk to the subject if enrolled or could interfere with evaluation of the vaccine or interpretation of study results (including neurologic or psychiatric conditions deemed likely to impair the quality of safety reporting)".

Interventions	**Vaccine type:** respiratory syncytial virus F particle vaccine formulations<br>**Arms extracted:** 2-Dose; 60µg RSV + 0.8 mg alum, 2-Dose; 60µg RSV + 0.4 mg alum; 2-Dose; 60 µg RSV + 0.2mg alum; 1-Dose; 60 µg RSV + 0.8 mg alum; 1-Dose; 60 µg RSV + 0.4 mg alum; 1-Dose; 60 µg RSV + 0.2 mg alum<br>In the meta-analyses, we defined the 0.2 mg alum groups (1st and second dose) as the comparison groups, and then divided the 0.2 mg alum groups (events and number analysed).<br>**Aluminium concentration:** 800 mcg, 400 mcg, 200 mcg<br>**Aluminium type:** aluminium phosphate<br>**Aluminium manufacturer:** Brenntag Biosector Denmark<br>**Vaccination schedule:** intramuscularly on days 0 and 28	
Outcomes	**Immunogenicity**: "serum IgG antibody titers specific for the F-Protein antigen across treatment groups (time frame: day 0 to day 56). Serum IgG antibody concentrations as ELISA units specific for the F protein antigen. Neutralizing antibody titer (time frame: day 0 to day 56). Kinetics of serum IgG antibody titers specific for the F-Protein antigen across time (time frame: day 0 to day 91). Antibodies sharing specificity with Palivizumab (time frame: day 0 to day 91)".<br>**Safety:** "(time frame: day 0 to day 182). Numbers and percentages of subjects with solicited local and systemic adverse events over the seven days post-injection; and all adverse events, solicited and unsolicited, including adverse changes in clinical laboratory parameters. In addition, medically attended events, serious adverse events, and significant new medical conditions will be collected for six months".	
Are there other groups in the trial not analysed in this review?	No	
Notes	**Contact with authors:** the author (allison.august11@gmail.com) was contacted on February 15, 2022 and enquired on the followings:<br>• proportion of participants experiencing each individual adverse events at maximum follow-up<br>• GMC or GMT and confidence intervals for each trial arm at maximum follow-up<br>• how was blinding of outcome assessors achieved<br>• proportion of participant who developed RSV after vaccination in each trial arm<br>No additional data provided.<br>**Funding:** this research was supported by Novavax and funded in part by PATH	
**Risk of bias assessment**		
**Bias**	**Authors' judgement**	**Support for judgement**
Random sequence generation	Unclear	Not described
Allocation concealment	Unclear	Not described
Blinding of participants and personnel	Unclear	Not described
Blinding of outcome assessment<br>*Safety*	Unclear	Not described

Blinding of outcome assessment *Immunogenicity*	Unclear	Not described
Incomplete outcome data *Safety*	Low	Drop outs < 5%
Incomplete outcome data *Immunogenicity*	High	Drop outs > 5%
Selective reporting	Low	Pre-published protocol. All outcomes reported. NCT01960686.
Other bias	Low	The trial appeared to be free of other issues that could put it at risk of bias.

De Kleijn 2000

Methods	Randomized, blinded and comparative trial
Participants	**Sex:** both<br>**Age:** toddlers<br>**Ethnicity:** mixed<br>**Diagnosis:** healthy<br>**Inclusion criteria:** health check and written informed consent.<br>**Exclusion criteria:** "temporary exclusion: administration of plasma products up to 3 months prior to the study, acute febrile illness (temperature > 38.5°C), evidence of serious disease demanding medical treatment, concurrent use of antibiotics. Definite exclusions: known allergy to one of the vaccine components, history of a severe reaction after vaccination, prior meningococcal disease, prior administration of meningococcal vaccine, immunodeficiency, congenital or chronical illness or a neurological disorder. No other vaccinations were given during this study"
Interventions	**Vaccine type:** monovalent meningococcal OMV vaccines<br>**Arms extracted:** monovalent meningococcal OMV vaccine with Al(OH)3 as adsorbent in two different vaccination schedules. Monovalent meningococcal OMV vaccine with AlPO4 as adsorbent in two different vaccination schedules.<br>**Aluminium concentration:** Al(OH)3 860 mcg, AlPO4 1340 mcg<br>**Aluminium type:** aluminium hydroxide and aluminium phosphate<br>**Aluminium manufacturer:** not reported<br>**Vaccination schedule:** "the two vaccine schedules used were: three vaccinations with a time interval between the vaccinations of 3–6 weeks, or two vaccinations with a time interval of 6–10 weeks. A booster vaccination was given 20–40 weeks after the last vaccination"
Outcomes	**Safety:** "the parents were asked to keep a diary for 7 days to record local and systemic adverse reactions. Between 18 and 30 h after each vaccination, the parents were contacted by telephone to assess the occurrence of side effects by a structured interview. During the next visit, adverse reactions, covering the 7-day period after vaccination, were registered by a structured interview and assessment of the diaries. Monitoring of local adverse reactions included the presence and size of redness, the presence and size of swelling, itching, degree of pain and the

	degree of not using the arm. Systemic adverse reactions registered were the presence of fever (> 38.5°C), the temperature, headache, listlessness, decreased appetite, nausea, joint complications, cutaneous symptoms, non-attendance to day-care, sleepiness, unusual crying, use of medication, contacts with the health care system and illness in the family".<br>**Immunogenicity:** serum bactericidal activity assay	
Are there other groups in the trial not analysed in this review?	No	
Notes	Table 1 listing the results of adverse events does not display results for dose 4 versus dose 3 in a way we could use for meta-analysis.<br>**Contact with authors:** the author (guy.berbers@rivm.nl) was contacted on February 15 2022 and enquired on the followings:<br>• Aluminium manufacturer<br>• Trial funding<br>• How was the blinding of outcome assessors achieved<br>• Proportion of participants with one or more non-serious adverse events at maximum follow-up<br>No additional data provided.<br>**Funding:** not reported	
**Risk of bias assessment**		
**Bias**	**Authors' judgement**	**Support for judgement**
Random sequence generation	Unclear	Not reported
Allocation concealment	Low	The vaccines and vaccination schedules were allocated by a list of random numbers.
Blinding of participants and personnel	Low	The vaccines with the two different adsorbents had identical appearance and differed only by coloured caps and lot numbers (red, 80003A; blue, 80002A). The investigators did not know the keys to these differences.
Blinding of outcome assessment<br>*Safety*	Unclear	Not described
Blinding of outcome assessment<br>*Immunogenicity*	Unclear	Not described
Incomplete outcome data<br>*Safety*	Low	Drop outs < 5%
Incomplete outcome data<br>*Immunogenicity*	Low	Drop outs < 5%
Selective reporting	Unclear	No pre-published protocol
Other bias	Low	The trial appeared to be free of other issues that could put it at risk of bias.
**Goubau 1992**		
Methods	Randomized double-blind comparison	
Participants	Sex: both<br>Age: adults	

	Ethnicity: not reported<br>Diagnosis: healthy<br>Inclusion criteria: volunteers were in good health at entry and women were advised not to become pregnant during the trial.<br>Exclusion criteria: "subjects were excluded from the study if they had travelled to a country of high HAV endemicity within the previous three months, had a history of liver disease or chronic alcohol consumption, were seropositive for anti- HAV antibodies or had hepatitis B surface antigen, or had elevated serum liver enzyme activity (alanine aminotransferase/aspartate aminotransferase, ALT/AST) at screening"
Interventions	**Vaccine type:** inactivated hepatitis A vaccine<br>**Arms extracted:** group C and D (vaccine + 1 mg aluminium vs vaccine + 0.5 mg aluminium)<br>**Aluminium concentration:** 1000 mcg and 500 mcg<br>**Aluminium type:** aluminium hydroxide<br>**Aluminium manufacturer:** not reported<br>**Vaccination schedule:** "three doses of the candidate inactivated hepatitis A vaccine were administered intramuscularly in the deltoid region at one-month intervals. A fourth vaccine dose, containing 720 E1.U of the HM175 formulation was administered to all subjects at month 12"
Outcomes	**Safety:** "a thorough physical examination was given, and a medical history was taken at the first visit, with further evaluation at months 1,2 and 12. On the day of each vaccination and for the three following days, systemic reactions (headache, gastrointestinal symptoms, dizziness, fatigue, fever) and local reactions (soreness, induration, redness, swelling) and any other findings were recorded by the vaccinee on symptom sheets. The temperature was recorded (°C) and local redness and induration were measured (ram). Other systemic and local symptoms were to be scored similarly by the vaccinee as absent, mild, moderate or severe".<br>**Immunogenicity:** "blood samples were taken at months 0, 1, 2, 3, 6, 12 and 13 for analysis of transaminases (ALT/AST) and for titration of anti-HAV antibodies. Two techniques were used to assay vaccine-induced anti-HAV antibodies in serum: enzyme-linked immunosorbent assay (ELISA) and radioimmunofocus inhibition test (RIFIT). Total antibodies against HAV were also measured using an ELISA inhibition assay"
Are there other groups in the trial not analysed in this review?	Yes
Notes	**Contact with authors:** the author (patrick.goubau@uclouvain.bel) was contacted on February 15, 2022 and enquired on the followings:<br>• Aluminium manufacturer<br>• Trial funding<br>• How was the blinding of outcome assessors achieved<br>• Proportion of participants with one or more serious adverse events at maximum follow-up (assigned per trial arm)<br>• All-cause mortality at maximum follow-up<br>No additional data provided.

	**Funding:** not reported	
**Risk of bias assessment**		
**Bias**	**Authors' judgement**	**Support for judgement**
Random sequence generation	Unclear	Not reported
Allocation concealment	Unclear	Not reported
Blinding of participants and personnel	Unclear	Not reported
Blinding of outcome assessment *Safety*	Unclear	Not reported
Blinding of outcome assessment *Immunogenicity*	Unclear	Not reported
Incomplete outcome data *Safety*	Unclear	Not reported
Incomplete outcome data *Immunogenicity*	Low	Drop outs < 5%
Selective reporting	High	No pre-published protocol. There were serious adverse events but those were not allocated per trial arm.
Other bias	Low	The trial appeared to be free of other issues that could put it at risk of bias.

Leroux-Roels 2013

Methods	Phase I/II, doubleblind, randomised, single-center, dose-ranging trial, with five parallel groups
Participants	**Sex:** both<br>**Age:** adults<br>**Ethnicity:** white<br>**Diagnosis:** healthy<br>**Inclusion criteria:** healthy HSV-seronegative men and women, aged 18–45 years.<br>**Exclusion criteria:** "pregnancy or lactation, any previous vaccination against herpes simplex, any previous administration of MPL and history of allergic disease likely to be exacerbated by the vaccination, history of convulsions, epilepsy, or any other signs of central nervous system disease, any suspected or confirmed immune disorder or immunosuppressive therapy"
Interventions	**Vaccine type:** glycoprotein D genital herpes vaccine<br>**Arms extracted:** group 3 Vs group 4 (vaccine + Al(OH)3 Vs vaccine + AlPO4)<br>**Aluminium concentration:** 500 mcg<br>**Aluminium type:** aluminium hydroxide and aluminium phosphate<br>**Aluminium manufacturer:** GSK<br>**Vaccination schedule:** vaccines were administered intramuscularly in the non-dominant deltoid according to a 0, 1 and 6 month schedule
Outcomes	**Safety:** "local and general AEs were recorded by the study participants on a diary card on the day of vaccination and for the three subsequent days. Additionally, unsolicited symptoms were recorded during 30 days after each dose and data regarding any SAEs were collected throughout the study period".

	**Immunogenicity:** "blood samples were collected for evaluation of anti-gD antibodies, anti-HSV neutralizing antibodies, and CMI at screening, prior to the first vaccination, one month post dose 1 (month 1) and dose 2 (month 2), 2 month post dose 2 (month 3), pre-dose 3 (month 6), one month post dose 3 (month 7) and six months post dose 3 (month 12). Seropositivity rates and GMTs, with 95% confidence intervals for anti-gD antibodies and anti-HSV neutralising antibodies were calculated by group for all time points for which blood samples were taken. Cell mediated immunity was evaluated through lymphoproliferation assays and quantification of IL-2 and IFN-γ secretion. Serological status at study enrolment and humoral immune response to vaccine were assessed by two ELISAs for anti-gD antibody titers"	
Are there other groups in the trial not analysed in this review?	Yes	
Notes	**Funding:** GlaxoSmithKline Biologicals SA took responsibility for all costs associated with this trial and development and publishing of the manuscript	
**Risk of bias assessment**		
**Bias**	**Authors' judgement**	**Support for judgement**
Random sequence generation	Low	Subjects were randomised on a 1:1:1:1:1 basis to receive one of the five vaccines formulations of the candidate herpes simplex vaccine. Monodose vials of the vaccines were coded according to randomization lists prepared by GlaxoSmithKline using an algorithm of pseudo random numbers
Allocation concealment	Low	Subjects eligible for inclusion in the trial were allocated a trial number in the order in which they were enrolled. Each subject received only the vaccines labelled with his/her trial number
Blinding of participants and personnel	Unclear	Not described
Blinding of outcome assessment *Safety*	Unclear	Not described
Blinding of outcome assessment *Immunogenicity*	Unclear	Not described
Incomplete outcome data *Safety*	Low	No drop outs
Incomplete outcome data *Immunogenicity*	Low	No drop outs
Selective reporting	Unclear	No pre-published protocol

Other bias	Low	The trial appeared to be free of other issues that could put it at risk of bias

Murphy 1983

Methods	Double-blind randomised trial
Participants	**Sex:** both<br>**Age:** children<br>**Ethnicity:** mixed<br>**Diagnosis:** healthy<br>**Inclusion criteria:** "children attending a comprehensive care program in a county hospital were recruited. They were of low socioeconomic status, and approximately 75% were Hispanic. Informed consent was obtained from one parent of each child who participated in the study".<br>**Exclusion criteria:** "those with low birth weight, neurologic or cardiovascular diseases, failure to thrive, or a family history of seizures were excluded from the study"
Interventions	**Vaccine type:** diphtheria-tetanus-pertussis vaccine<br>**Arms extracted:** extracted antigen with aluminium phosphate adjuvant; extracted antigen with alum adjuvant; whole cell antigen with alum adjuvant; whole cell antigen with aluminium phosphate adjuvant.<br>**Aluminium concentration:** not reported<br>**Aluminium type:** aluminium phosphate and alum<br>**Aluminium manufacturer:** not reported<br>**Vaccination schedule:** three injections administered intramuscularly in the midlateral thigh muscle
Outcomes	**Safety:** "parents were instructed in taking temperatures, and they were asked to record temperature between 6 and 12 hours after vaccination and the following morning. A card was provided on which parents would check (1) if the injection site was red, warm, tender, discolored, or otherwise abnormal; (2) if there was any unusual crying or screaming; (3) if the baby received any medication; and (4) other comments. Parents were asked to return the following day, at which time the card was reviewed, their child was examined by the pediatric nurse practitioner and a pediatrician, and a rectal temperature was taken. The reactogenicity of the four preparations was assessed by evaluating systemic and local responses. Local reactions were evaluated at the clinic visit 24 hours after immunization at which time local erythema or induration was measured and parental records were reviewed".<br>**Immunogenicity:** "sera were obtained from 42 patients prior to the first immunization and 2 months after administration of the third dose of vaccine. Pertussis antibody was evaluated utilizing the microagglutination technique developed by Manclark. Diphtheria antitoxin response was assayed utilizing a cell culture technique.The indirect hemagglutination technique utilized by the Centers for Disease Control was employed to assay tetanus antitoxin"
Are there other groups in the trial not analysed in this review?	No
Notes	**Contact with authors:** not found

	**Funding:** not reported	
**Risk of bias assessment**		
Bias	Authors' judgement	Support for judgement
Random sequence generation	Unclear	Assignment to one of the four groups was accomplished by a randomization table based on the enrollee number
Allocation concealment	Unclear	Not reported
Blinding of participants and personnel	Low	The vaccine provided by the manufacturer was coded so that neither the investigators nor the parents of the vaccinees were aware of the composition of the material administered
Blinding of outcome assessment<br>*Safety*	Unclear	Not described
Blinding of outcome assessment<br>*Immunogenicity*	Low	Sera, which were sent to the manufacturer for antibody determination, were coded so that the testers were unaware of which vaccine the serum donor had received
Incomplete outcome data<br>*Safety*	Unclear	n.a.
Incomplete outcome data<br>*Immunogenicity*	Unclear	n.a.
Selective reporting	Unclear	No pre-published protocol
Other bias	Low	The trial appeared to be free of other issues that could put it at risk of bias
**NCT00562237**		
Methods	Randomized, Observer-Blind, Placebo-Controlled trial	
Participants	**Sex:** both<br>**Age:** adults<br>**Ethnicity:** not reported<br>**Diagnosis:** healthy<br>**Inclusion criteria:** "being healthy and ≥ 18 and ≤ 49 years of age. Willing and able to give informed consent".<br>**Exclusion criteria:** "having participated in an influenza H5 vaccine trial in the past. Known to be allergic to any constituent of the vaccine. Serious adverse reactions to previous (influenza) vaccination. Currently participating in another clinical trial or having participated in any clinical trial in the month preceding the start of the study. Using medication that influences the immune system"	
Interventions	**Vaccine type:** egg-derived pandemic surface antigen influenza vaccine<br>**Arms extracted:** S205 10 mcg HA + 500 mcg AlOH; S205 30 mcg HA + 500 mcg AlOH; S205 10 mcg HA + 1250 mcg AlOH; S205 30 mcg HA + 1250 mcg AlOH<br>**Aluminium concentration:** 500 mcg, 125 mcg<br>**Aluminium type:** aluminium hydroxide	

	**Aluminium manufacturer:** not reported<br>**Vaccination schedule:** 2 intramuscular injections	
Outcomes	Safety and immunogenicity	
Are there other groups in the trial not analysed in this review?	Yes	
Notes	This trial discontinued on 20 June 2008 because the interim immunogenicity results do not justify these formulations as pandemic vaccine candidates. No results reported	
**Risk of bias assessment**		
**Bias**	**Authors' judgement**	**Support for judgement**
Random sequence generation	Unclear	Not described
Allocation concealment	Unclear	Not described
Blinding of participants and personnel	Unclear	Not described
Blinding of outcome assessment<br>*Safety*	Unclear	Not described
Blinding of outcome assessment<br>*Immunogenicity*	Unclear	Not described
Incomplete outcome data<br>*Safety*	Unclear	Not described
Incomplete outcome data<br>*Immunogenicity*	Unclear	Not described
Selective reporting	Unclear	n.a.
Other bias	Unclear	n.a.

Rupp 2019

Methods	Phase 1/2 randomised, double-blind, multicenter trial
Participants	**Sex:** both<br>**Age:** infants<br>**Ethnicity:** mixed<br>**Diagnosis:** healthy<br>**Inclusion criteria:** healthy and able to attend all scheduled visits.<br>**Exclusion criteria:** "prior administration of any pneumococcal vaccine, any non-live vaccine within 14 days, or any live vaccine within 30 days. History of invasive pneumococcal disease. Known hypersensitivity to any vaccine component. Received systemic corticosteroids within 14 days of first vaccination. Known or suspected impairment of immune function. Febrile illness within 72 hours before vaccination. Received blood transfusion or blood products within 30 days. Mother has documented human immunodeficiency virus or is hepatitis B surface antigen positive. Has asplenia or failure to thrive"
Interventions	**Vaccine type:** 15-valent pneumococcal conjugate vaccine (PCV15)<br>**Arms extracted:** 3 vs 1<br>3: all serotypes at 2 µg/dose, except 6B at 4 µg/dose. Alum 250 µg/dose<br>1: all serotypes at 2 µg/dose, except 6B at 4 µg/dose. Alum 125 µg/dose<br>**Aluminium concentration:** 250 mcg and 125 mcg<br>**Aluminium type:** aluminium phosphate<br>**Aluminium manufacturer:** not reported

	**Vaccination schedule:** 4-dose regimen at 2, 4, 6, and 12–15 months of age
Outcomes	**Safety:** "subjects were followed for adverse events for 14 days following each vaccination using validated vaccination report card. In both adult and infant cohorts, solicited injection-site (local) AEs included redness, swelling, hard lump, and pain/tenderness. Solicited systemic AEs in the adult cohort included headache, fatigue, muscle pain, and joint pain while irritability, drowsiness, hives/welts, and appetite lost were solicited in the infant cohort. Serious AEs were collected during entire study period and/or study completion. Body temperatures (rectal/axillary) were recorded for 7 days following each vaccination. Body temperature was also taken days 8–14 postvaccination if fever was suspected".<br>**Immunogenicity:** "blood was collected approximately 30 days postdose 3, pre-dose 4, and 30 days postdose 4. Sera were used to measure IgG using pneumococcal electrochemiluminescence (Pn-ECL) assay, and opsonophagocytic (OPA) killing activity using multiplex OPA (MOPA-4) assay to all 15 vaccine serotypes"
Are there other groups in the trial not analysed in this review?	Yes
Notes	**Contact with authors:** the author (luwy_musey@merck.com) was contacted on February 15, 2022 and enquired on the followings:<br>• How was the blinding of outcome assessors achieved<br>• Aluminium manufacturer<br>No additional data provided.<br>**Funding:** Merck Sharp & Dohme Corp., a subsidiary of Merck & Co., Inc., Kenilworth, NJ (sponsor)

Risk of bias assessment

**Bias**	**Authors' judgement**	**Support for judgement**
Random sequence generation	Unclear	Not described
Allocation concealment	Unclear	Not described
Blinding of participants and personnel	Unclear	Not described
Blinding of outcome assessment *Safety*	Unclear	Masking: triple (Participant, Care Provider, Investigator)
Blinding of outcome assessment *Immunogenicity*	Unclear	Masking: triple (Participant, Care Provider, Investigator)
Incomplete outcome data *Safety*	Low	Drop outs < 5%
Incomplete outcome data *Immunogenicity*	High	Drop outs > 5%
Selective reporting	Low	Pre-published protocol. All the outcomes were reported. NCT02037984
Other bias	Low	The trial appeared to be free of other issues that could put it at risk of bias

Schwameis 2016

Methods	Randomised, double-blind, adjuvant-controlled, dose escalation first-in-man trial
Participants	**Sex:** both<br>**Age:** adults<br>**Ethnicity:** white<br>**Diagnosis:** healthy<br>**Inclusion criteria:** "signed informed consent obtained before any trial-related activities. Ability to comprehend the full nature and purpose of the study, including possible risks and side effects; ability to co-operate with the investigator and to comply with the requirements of the entire study. Men or women aged >18 and <65 years. In female subjects a negative urine pregnancy test during screening and the willingness not to become pregnant during the entire study period by practising reliable methods of contraception. Normal findings in the medical history and by physical examination or the investigator considers all abnormalities to be clinically irrelevant. Normal laboratory values or the investigator considers all abnormalities to be clinically irrelevant".<br>**Exclusion criteria:** "treatment with an investigational drug within one month prior to this trial. History of immunodeficiency or immunosuppressive therapy, known Human Immunodeficiency Virus (HIV) infection or Hepatitis B/C infection. Drug addiction including alcohol dependence. Inability or unwillingness to avoid more than the usual intake of alcohol during the 48 hours after vaccination. Blood donations during the 1st month prior to this study. Recent infection (within 1 week prior to visit 1). Relevant history of renal, hepatic, gastrointestinal, cardiovascular, respiratory, skin, hematological, endocrine, inflammatory or neurological diseases, that in the opinion of the investigator may interfere with the aim of the study. Ascertained or presumed hypersensitivity to the active principle and/or formulations' ingredients; history of anaphylaxis to drugs or major allergic reactions in general, which the investigator considers may compromise the safety of the volunteers. Clinically relevant abnormal laboratory values indicative of physical illness or signs and symptoms of relevant autoimmunity. Use of medication during 2 weeks before the visit 1, which the investigator considers may affect the validity of the study except hormonal contraception in female subjects; prior to taking any medication during 72 h prior to visit 1, the study center should be consulted. Pregnancy (positive pregnancy test at screening or during study phase), lactation or unreliable contraception in female subjects with child-bearing potential. Subjects with any condition, which in the opinion of the investigator makes the subject unsuitable for inclusion. Inability or unwillingness to provide informed consent and to abide by the requirements of the study. Baseline TSST-1 Ab titer > 1:2000"
Interventions	**Vaccine type:** recombinant toxic shock syndrome toxin (rTSST)-1 variant vaccine<br>**Arms extracted:** $Al(OH)_3$ 1mg; $Al(OH)_3$ 600 mcg; $Al(OH)_3$ 200 mcg<br>**Aluminium concentration:** 1 mg, 600 mcg, 200 mcg<br>**Aluminium type:** aluminium hydroxide<br>**Aluminium manufacturer:** Biomedizinische Forschungs GmbH

<table>
<tr><td></td><td colspan="2">Vaccination schedule: participants were vaccinated intramuscularly on days 0 and 42</td></tr>
<tr><td>Outcomes</td><td colspan="2">Safety: "the primary study endpoint was safety and tolerability of the rTSST-1v vaccine in the safety population through day 70".
Immunogenicity: "the secondary outcome measure was immunogenicity on day 14 in the per-protocol population confirmed by the presence of antibodies against rTSST-1v measured by ELISA IgG and antibody functionality assessed by thymidine incorporation assay and PCR to test for TSST-1-dependent T-cell activation and interleukin-2 gene expression in vitro"</td></tr>
<tr><td>Are there other groups in the trial not analysed in this review?</td><td colspan="2">Yes</td></tr>
<tr><td>Notes</td><td colspan="2">Contact with authors: the author (martha.eibl@meduniwien.ac.at) was contacted on February 15 2022 and enquired on the followings for each of the aluminium arms (results is the publication are pooled for all the arms):
All-cause mortality
Proportion of participants with one or more serious adverse events
Proportion of participants with one or more non-serious adverse events
Serological response
No additional data provided.
Funding: Biomedizinische Forschungs GmbH</td></tr>
<tr><td colspan="3">Risk of bias assessment</td></tr>
<tr><td>Bias</td><td>Authors' judgement</td><td>Support for judgement</td></tr>
<tr><td>Random sequence generation</td><td>Low</td><td>Participants were randomly assigned by block randomisation (block sizes of three and 12). Randomisation was done by a pharmacist at the trial site using opaque, sealed envelopes containing consecutive participant numbers with the particular group allocation and vaccine volume</td></tr>
<tr><td>Allocation concealment</td><td>Low</td><td>Trial investigators and participants were masked to group allocation. The trial drugs were likewise prepared by a pharmacist not otherwise involved in the trial and delivered in ready-to-use syringes to the investigator</td></tr>
<tr><td>Blinding of participants and personnel</td><td>Low</td><td>Trial investigators and participants were masked to group allocation. The trial drugs were likewise prepared by a pharmacist not otherwise involved in the trial and</td></tr>
</table>

		delivered in ready-to-use syringes to the investigator
Blinding of outcome assessment<br>*Safety*	Low	Trial investigators and participants were masked to group allocation. The trial drugs were likewise prepared by a pharmacist not otherwise involved in the trial and delivered in ready-to-use syringes to the investigator. Safety monitoring was the investigator's responsibility
Blinding of outcome assessment<br>*Immunogenicity*	Low	Analysis of serological endpoints was done by employees of the funder in a blinded fashion under GLP conformity
Incomplete outcome data<br>*Safety*	Unclear	Not reported
Incomplete outcome data<br>*Immunogenicity*	Unclear	Not reported
Selective reporting	Low	Pre-published protocol. NCT02340338
Other bias	Low	The trial appeared to be free of other issues that could put it at risk of bias

**Theeten 2005**	
Methods	Randomised trial with balanced allocation (1:1:1) in three parallel groups
Participants	**Sex:** both<br>**Age:** adolescents<br>**Ethnicity:** mixed<br>**Diagnosis:** healthy<br>**Inclusion criteria:** "healthy adolescents. Subjects were to have received, to the best of their knowledge, primary and booster immunisation according to the Belgian vaccination schedule (DTPw vaccine at 3, 4, 5 months and 13 months of age, DT vaccine at 6 years of age)".<br>**Exclusion criteria:** "subjects had to be free of obvious health problems as established by medical history and clinical examination (including axillary body temperature) before entering into the trial. Specific exclusion criteria for enrolment were: history of neurologic disease, immunosuppression, previous or intercurrent diphtheria, tetanus or pertussis disease, vaccination against these diseases within the previous five years, previous vaccination with a meningococcal conjugate vaccine (because these contain diphtheria or tetanus toxoid as carrier, which could influence the booster response of the study vaccine). Other exclusion criteria included receipt of blood products in the previous 3 months, administration of any other vaccine from 30 days before to 30 days after administration of the study vaccine, known hypersensitivity to a component of the study vaccine or history of any serious adverse reaction following DTPw vaccination"

Interventions	**Vaccine type:** dTpa vaccine<br>**Arms extracted:** vaccine + 500 mcg aluminium; vaccine + 300 mcg aluminium; vaccine + 133 mcg vaccine<br>**Aluminium concentration:** 500 mcg, 300 mcg, 133 mcg<br>**Aluminium type:** aluminium hydroxide<br>**Aluminium manufacturer:** GSK<br>**Vaccination schedule:** one dose intramuscularly into the non-dominant deltoid muscle
Outcomes	**Safety:** "subjects self-monitored adverse events using a diary card to record local redness, swelling and pain, and systemic symptoms of fatigue, fever (temperature ≥ 37.5 ◦C by oral or axillary route), headache, malaise and vomiting, occurring on the day of vaccination and during 14 subsequent days, with indication of intensity. The investigator assessed the relationship of any general symptoms to vaccination. The follow-up period for unsolicited symptoms, serious adverse events and the use of concomitant medication was 30 days. Subjects were asked to contact the study personnel and return to the study centre for assessment in case of large swelling reactions (diameter >100 mm, or, diffuse swelling, or, noticeable increase in limb circumference) at injection site".<br>**Immunogenicity:** "antibodies against diphtheria and tetanus toxoids were measured using a modified sandwich ELISA assay and expressed in International Units per millilitre (IU/ml), with respect to a reference serum. Pertussis antibodies (anti-PT, anti FHA and anti-PRN) were measured using an ELISA assay and expressed in ELISA units per millilitre (El.U/ml), with an assay cut-off of 5 El.U/ml.The immunogenicity of the three vaccine formulations was assessed by investigating booster response rates to all vaccine antigens and geometric mean antibody concentrations elicited by each of the formulations"
Are there other groups in the trial not analysed in this review?	No
Notes	**Contact with authors:** the author (heidi.theeten@uantwerpen.be) was contacted on February 15, 2022 and enquired on the followings for each of the aluminium arms (results is the publication are pooled for all the arms):<br>• Trial funding<br>• All-cause mortality<br>• Proportion of participants with one or more serious adverse events (there are 3 mentioned in the publication but not assigned per trial arm) at maximum follow-up<br>• Proportion of participants with one or more non-serious adverse events at maximum follow-up<br>No additional data provided.<br>**Funding:** not reported

Risk of bias assessment

Bias	Authors' judgement	Support for judgement
Random sequence generation	Unclear	Not described
Allocation concealment	Unclear	Not described
Blinding of participants and personnel	Unclear	Not described

Blinding of outcome assessment *Safety*	Low	Due to the different visual characteristics of the liquid formulations the trial was observer-blind: the vaccine was administered by a different person from the observer designated to perform the safety follow-up
Blinding of outcome assessment *Immunogenicity*	Low	All serological assays were performed in a blinded fashion
Incomplete outcome data *Safety*	Low	Drop outs < 5%
Incomplete outcome data *Immunogenicity*	Low	Drop outs < 5%
Selective reporting	High	No pre-published protocol. There were serious adverse events but those were not allocated per trial arm
Other bias	Low	The trial appeared to be free of other issues that could put it at risk of bias
**Villa 2006**		
Methods	Phase II, randomised, multi-center, double-blind, placebo-controlled trial	
Participants	**Sex:** females<br>**Age:** mixed<br>**Ethnicity:** mixed<br>**Diagnosis:** healthy<br>**Inclusion criteria:** "to enrich the trial population for HPV-naıve women, only non-pregnant, healthy women who reported no prior abnormal Pap smears of low-grade squamous intraepithelial lesion or worse, and reported a lifetime history of four or fewer male sex partners were enrolled. Women who were anti- HPV seropositive (i.e., had developed immune responses to an HPV infection) and women who were HPV DNA positive (i.e., had evidence of ongoing HPV infection) were enrolled".<br>**Exclusion criteria:** "among virgins, enrolment was limited to those women who were ≥18 years of age and seeking contraception. This trial did not exclude subjects with prior or ongoing HPV infection of any type"	
Interventions	**Vaccine type:** placebo<br>**Arms extracted:** placebo 450 mcg aluminium; placebo 225 mcg aluminium<br>**Aluminium concentration:** 450 mcg; 225 mcg<br>**Aluminium type:** amorphous aluminium hydroxyphosphate sulfate adjuvant (AAHS)<br>**Aluminium manufacturer:** Merck Research Laboratories, West Point, PA<br>**Vaccination schedule:** day 1, Month 2, and Month 6, given as a 0.5mL intramuscular injection	
Outcomes	**Safety:** "temperatures were recorded orally for 5 days following each injection. All adverse experiences were collected daily by	

	the participant on aVaccination Report Card for 14 days following each vaccination.<br>**Immunogenicity:** Serum antibodies to HPV 6, 11, 16 and 18 were measured using a competitive radioimmunoassay (cRIA) or a competitive Luminex immunoassay (cLIA) in both vaccine and placebo groups. Serum samples were obtained from all subjects at day 1 and at Months 2, 3, 6, 7, 12, 18, 24, 30, and 36"	
Are there other groups in the trial not analysed in this review?	Yes	
Notes	**Contact with authors:** the author (alfred_saah@merck.com) was contacted on February 15, 2022 and enquired on the followings for each of the aluminium arms (results is the publication are pooled for all the arms):<br>• GMT and confidence intervals for each trial arms at maximum follow<br>No additional data provided.<br>**Funding:** Merck Research Laboratories, a Division of Merck & Company Inc.	
**Risk of bias assessment**		
**Bias**	**Authors' judgement**	**Support for judgement**
Random sequence generation	Unclear	Not described
Allocation concealment	Unclear	Not described
Blinding of participants and personnel	Low	Subjects, investigators (and their staff), HPV vaccine clinical, data management, statistics, regulatory, and quality assurance personnel at the Sponsor, Pathology Panel members, and laboratory personnel conducting the PCR and serology assays were not informed of individual vaccination allocations until the end of the trial
Blinding of outcome assessment<br>*Safety*	Low	With the exception of a designated unblinded statistician, in-house blinding was maintained until the completion of the trial. The designated unblinded statistician was appointed to perform the safety and immunogenicity interim analysis on data through the Month 7 time point. Subjects, investigators (and their staff), HPV vaccine clinical, data management, statistics, regulatory, and quality assurance personnel at the Sponsor, Pathology Panel members, and laboratory personnel conducting the PCR

		and serology assays were not informed of individual vaccination allocations until the end of the trial
Blinding of outcome assessment *Immunogenicity*	Low	With the exception of a designated unblinded statistician, in-house blinding was maintained until the completion of the trial. The designated unblinded statistician was appointed to perform the safety and immunogenicity interim analysis on data through the Month 7 time point. Subjects, investigators (and their staff),HPVvaccine clinical, data management, statistics, regulatory, and quality assurance personnel at the Sponsor, Pathology Panel members, and laboratory personnel conducting the PCR and serology assays were not informed of individual vaccination allocations until the end of the trial
Incomplete outcome data *Safety*	Low	Drop outs < 5%
Incomplete outcome data *Immunogenicity*	Low	Drop outs < 5%
Selective reporting	Unclear	No pre-published protocol
Other bias	Unclear	The trial appeared to be free of other issues that could put it at risk of bias

ADDITIONAL INFORMATION AND ANALYSES ON THE OUTCOMES

Comparison 1 – Higher vs lower aluminium concentration

Individual serious adverse events

Comparison 1. Individual serious adverse events reported by the included trials

Type of serious adverse event	Trial	Trial Group	Events / analysed
**Chlamydial cervicitis**	August 2017	400 mcg aluminium phosphate (2 doses)	1/89
**Pneumonia**	August 2017	800 mcg aluminium phosphate (1 dose)	1/93
**Sepsis**	August 2017	800 mcg aluminium phosphate (1 dose)	1/93
**Borderline mucinous tumour of ovary**	August 2017	400 mcg aluminium phosphate (2 doses)	1/89
**Ovarian germ cell teratoma benign**	August 2017	800 mcg aluminium phosphate (1 dose)	1/93
**Squamous cell carcinoma of the cervix**	August 2017	800 mcg aluminium phosphate (2 doses)	1/91
**Brain injury**	August 2017	800 mcg aluminium phosphate (1 dose)	1/93
**Abortion spontaneous**	August 2017	400 mcg aluminium phosphate (1 dose)	1/89
	August 2017	200 mcg aluminium phosphate (1 dose)	1/90
**Ectopic pregnancy**	August 2017	400 mcg aluminium phosphate (1 dose)	1/89
**Anxiety**	August 2017	800 mcg aluminium phosphate (1 dose)	1/93
**Borderline personality disorder**	August 2017	800 mcg aluminium phosphate (1 dose)	1/93
**Major depression**	August 2017	800 mcg aluminium phosphate (1 dose)	1/93
**Suicidal ideation**	August 2017	800 mcg aluminium phosphate (1 dose)	1/93
**Suicide attempt**	August 2017	800 mcg aluminium phosphate (1 dose)	1/93
**Calculus ureteric**	August 2017	800 mcg aluminium phosphate (2 doses)	1/91
**Dysfunctional uterine bleeding**	August 2017	800 mcg aluminium phosphate (2 doses)	1/91
**Pneumonia aspiration**	August 2017	800 mcg aluminium phosphate (1 dose)	1/93
**Gastroenteritis viral**	Rupp 2019	250 mcg aluminium phosphate	0/50
	Rupp 2019	125 mcg aluminium phosphate	1/50

Individual adverse events

Injection site pain

Study or Subgroup	Higher concentrations Events	Higher concentrations Total	Lower concentrations Events	Lower concentrations Total	Weight	Risk ratio M-H, Random, 95% CI
**5.1.1 Aluminium phosphate (2 doses)**						
August 2017 (1)	47	85	16	43	2.6%	1.49 [0.96 , 2.29]
August 2017 (2)	48	86	30	43	6.0%	0.80 [0.61 , 1.05]
**Subtotal (95% CI)**		**171**		**86**	**8.6%**	**1.07 [0.57 , 2.01]**
Total events:	95		46			
Heterogeneity: Tau² = 0.18; Chi² = 6.16, df = 1 (P = 0.01); I² = 84%						
Test for overall effect: Z = 0.20 (P = 0.84)						
**5.1.2 Aluminium phosphate (1 dose)**						
August 2017 (3)	7	85	5	43	0.4%	0.71 [0.24 , 2.10]
August 2017 (4)	6	85	5	43	0.4%	0.61 [0.20 , 1.88]
Rupp 2019 (5)	42	50	47	50	16.2%	0.89 [0.78 , 1.03]
**Subtotal (95% CI)**		**220**		**136**	**17.1%**	**0.89 [0.77 , 1.02]**
Total events:	55		57			
Heterogeneity: Tau² = 0.00; Chi² = 1.12, df = 2 (P = 0.57); I² = 0%						
Test for overall effect: Z = 1.74 (P = 0.08)						
**5.1.3 Aluminium hydroxide**						
Theeten 2005 (6)	202	224	97	107	28.8%	0.99 [0.92 , 1.07]
Theeten 2005 (6)	187	209	97	107	28.4%	0.99 [0.91 , 1.07]
**Subtotal (95% CI)**		**433**		**214**	**57.2%**	**0.99 [0.94 , 1.05]**
Total events:	389		194			
Heterogeneity: Tau² = 0.00; Chi² = 0.02, df = 1 (P = 0.89); I² = 0%						
Test for overall effect: Z = 0.33 (P = 0.74)						
**5.1.4 Amorphous aluminium hydroxyphosphate sulfate**						
Villa 2006 (7)	111	140	98	135	17.1%	1.09 [0.96 , 1.25]
**Subtotal (95% CI)**		**140**		**135**	**17.1%**	**1.09 [0.96 , 1.25]**
Total events:	111		98			
Heterogeneity: Not applicable						
Test for overall effect: Z = 1.29 (P = 0.20)						
**Total (95% CI)**		**964**		**571**	**100.0%**	**0.99 [0.92 , 1.06]**
Total events:	650		395			
Heterogeneity: Tau² = 0.00; Chi² = 11.19, df = 7 (P = 0.13); I² = 37%						
Test for overall effect: Z = 0.41 (P = 0.68)						
Test for subgroup differences: Chi² = 4.67, df = 3 (P = 0.20), I² = 35.7%						

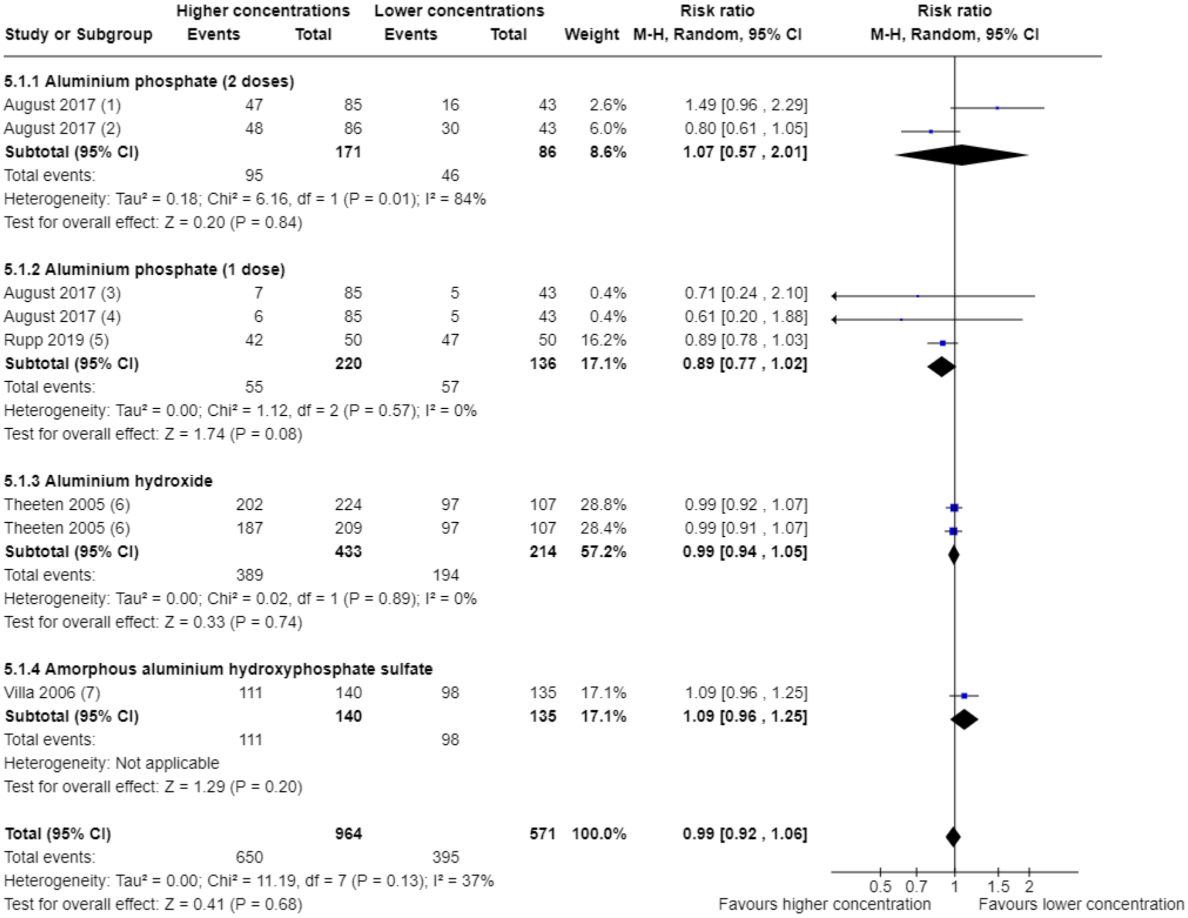

Footnotes
(1) 2-Dose; 60 µg RSV + 0.8 mg aluminium phosphate vs 2-Dose; 60µg RSV + 0.2 mg aluminium phosphate
(2) 2-Dose; 60 µg RSV + 0.4 mg aluminium phosphate vs 2-Dose; 60µg RSV + 0.2 mg aluminium phosphate
(3) 1-Dose; 60 µg RSV + 0.8 mg aluminium phosphate vs 1-Dose; 60 µg RSV + 0.2 mg aluminium phosphate
(4) 1-Dose; 60 µg RSV + 0.4 mg aluminium phosphate vs 1-Dose; 60 µg RSV + 0.2 mg aluminium phosphate
(5) 250 mcg aluminium phosphate vs 125 mcg aluminium phosphate
(6) 0.3 mg aluminium hydroxide vs 0.133 mg aluminium hydroxide
(7) 450 mcg amorphous aluminium hydroxyphosphate sulfate vs 225 mcg amorphous aluminium hydroxyphosphate sulfate

Injection site redness

Study or Subgroup	Higher concentrations Events	Higher concentrations Total	Lower concentrations Events	Lower concentrations Total	Weight	Risk ratio M-H, Random, 95% CI
**5.2.1 Aluminium phosphate (2 doses)**						
August 2017 (1)	12	85	1	43	3.3%	6.07 [0.82 , 45.16]
August 2017 (2)	8	86	1	43	3.2%	4.00 [0.52 , 30.96]
**Subtotal (95% CI)**		**171**		**86**	**6.5%**	**4.95 [1.18 , 20.73]**
Total events:	20		2			
Heterogeneity: Tau² = 0.00; Chi² = 0.08, df = 1 (P = 0.77); I² = 0%						
Test for overall effect: Z = 2.19 (P = 0.03)						
**5.2.2 Aluminium phosphate (1 dose)**						
August 2017 (3)	1	85	1	43	1.8%	0.51 [0.03 , 7.89]
August 2017 (4)	1	85	0	43	1.4%	1.53 [0.06 , 36.91]
**Subtotal (95% CI)**		**170**		**86**	**3.2%**	**0.81 [0.10 , 6.50]**
Total events:	2		1			
Heterogeneity: Tau² = 0.00; Chi² = 0.27, df = 1 (P = 0.60); I² = 0%						
Test for overall effect: Z = 0.20 (P = 0.85)						
**5.2.3 Aluminium hydroxide**						
Theeten 2005 (5)	56	224	31	107	44.6%	0.86 [0.59 , 1.25]
Theeten 2005 (6)	67	209	30	107	45.8%	1.14 [0.80 , 1.64]
**Subtotal (95% CI)**		**433**		**214**	**90.3%**	**1.00 [0.76 , 1.31]**
Total events:	123		61			
Heterogeneity: Tau² = 0.00; Chi² = 1.12, df = 1 (P = 0.29); I² = 11%						
Test for overall effect: Z = 0.02 (P = 0.98)						
**Total (95% CI)**		**774**		**386**	**100.0%**	**1.10 [0.76 , 1.59]**
Total events:	145		64			
Heterogeneity: Tau² = 0.04; Chi² = 6.46, df = 5 (P = 0.26); I² = 23%						
Test for overall effect: Z = 0.49 (P = 0.62)						
Test for subgroup differences: Chi² = 4.69, df = 2 (P = 0.10), I² = 57.4%						

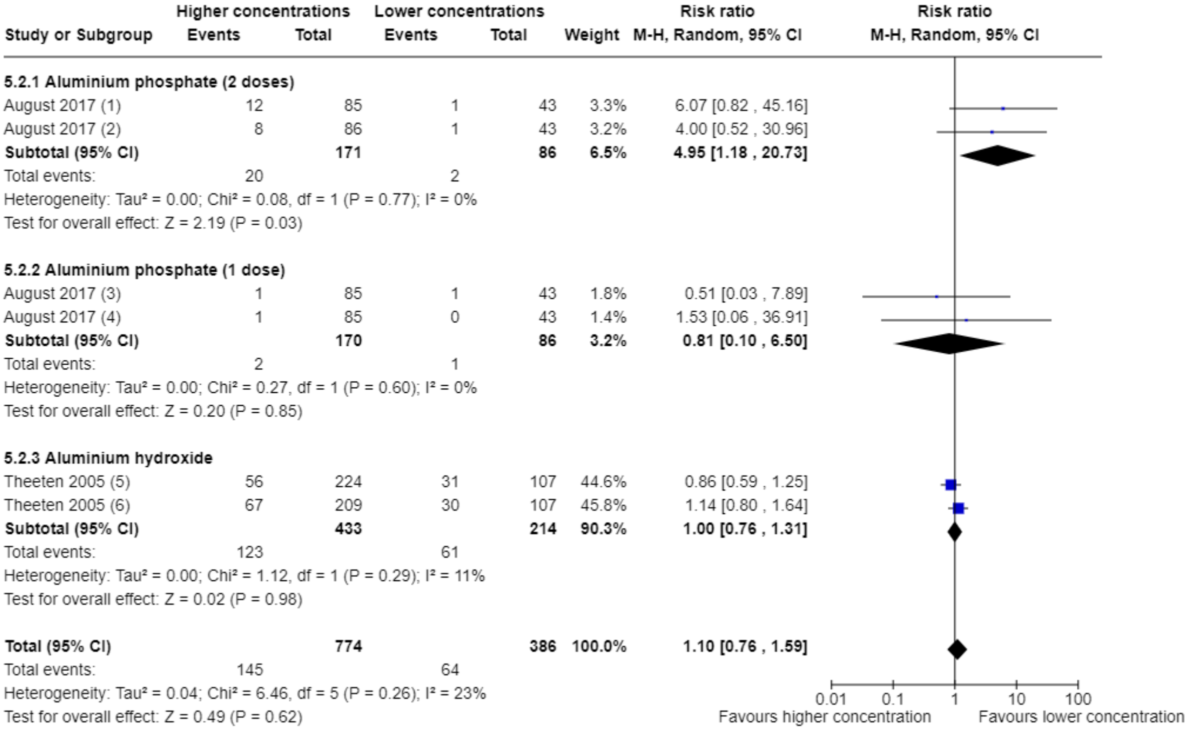

Footnotes

(1) 2-Dose; 60 µg RSV + 0.8 mg aluminium phosphate vs 2-Dose; 60 µg RSV + 0.2 mg aluminium phosphate
(2) 2-Dose; 60 µg RSV + 0.4 mg aluminium phosphate vs 2-Dose; 60 µg RSV + 0.2 mg aluminium phosphate
(3) 1-Dose; 60 µg RSV + 0.8 mg aluminium phosphate vs 1-Dose; 60 µg RSV + 0.2 mg aluminium phosphate
(4) 1-Dose; 60 µg RSV + 0.4 mg aluminium phosphate vs 1-Dose; 60 µg RSV + 0.2 mg aluminium phosphate
(5) 0.5 mg aluminium hydroxide vs 0.133 mg aluminium hydroxide
(6) 0.3 mg aluminium hydroxide vs 0.133 mg aluminium hydroxide

Injection site swelling

Study or Subgroup	Higher concentrations Events	Higher concentrations Total	Lower concentrations Events	Lower concentrations Total	Weight	Risk ratio M-H, Random, 95% CI
**5.3.1 Aluminium phosphate (2 doses)**						
August 2017 (1)	9	86	2	43	1.8%	2.25 [0.51 , 9.96]
August 2017 (2)	14	85	2	43	1.9%	3.54 [0.84 , 14.88]
**Subtotal (95% CI)**		**171**		**86**	**3.6%**	**2.85 [1.01 , 8.00]**
Total events:	23		4			
Heterogeneity: Tau² = 0.00; Chi² = 0.19, df = 1 (P = 0.67); I² = 0%						
Test for overall effect: Z = 1.98 (P = 0.05)						
**5.3.2 Aluminium phosphate (1 dose)**						
August 2017 (3)	2	85	0	43	0.4%	2.56 [0.13 , 52.13]
August 2017 (4)	0	85	1	43	0.4%	0.17 [0.01 , 4.10]
Rupp 2019 (5)	22	50	19	50	17.4%	1.16 [0.72 , 1.86]
**Subtotal (95% CI)**		**220**		**136**	**18.2%**	**1.13 [0.71 , 1.80]**
Total events:	24		20			
Heterogeneity: Tau² = 0.00; Chi² = 1.66, df = 2 (P = 0.44); I² = 0%						
Test for overall effect: Z = 0.53 (P = 0.60)						
**5.3.3 Aluminium hydroxide**						
Theeten 2005 (6)	67	224	33	107	32.2%	0.97 [0.69 , 1.37]
Theeten 2005 (7)	67	209	32	107	31.5%	1.07 [0.75 , 1.52]
**Subtotal (95% CI)**		**433**		**214**	**63.7%**	**1.02 [0.80 , 1.30]**
Total events:	134		65			
Heterogeneity: Tau² = 0.00; Chi² = 0.16, df = 1 (P = 0.69); I² = 0%						
Test for overall effect: Z = 0.15 (P = 0.88)						
**5.3.4 Amorphous aluminium hydroxyphosphate sulfate**						
Villa 2006 (8)	29	140	20	135	14.5%	1.40 [0.83 , 2.35]
**Subtotal (95% CI)**		**140**		**135**	**14.5%**	**1.40 [0.83 , 2.35]**
Total events:	29		20			
Heterogeneity: Not applicable						
Test for overall effect: Z = 1.27 (P = 0.20)						
**Total (95% CI)**		**964**		**571**	**100.0%**	**1.13 [0.93 , 1.37]**
Total events:	210		109			
Heterogeneity: Tau² = 0.00; Chi² = 6.49, df = 7 (P = 0.48); I² = 0%						
Test for overall effect: Z = 1.21 (P = 0.23)						
Test for subgroup differences: Chi² = 4.39, df = 3 (P = 0.22), I² = 31.7%						

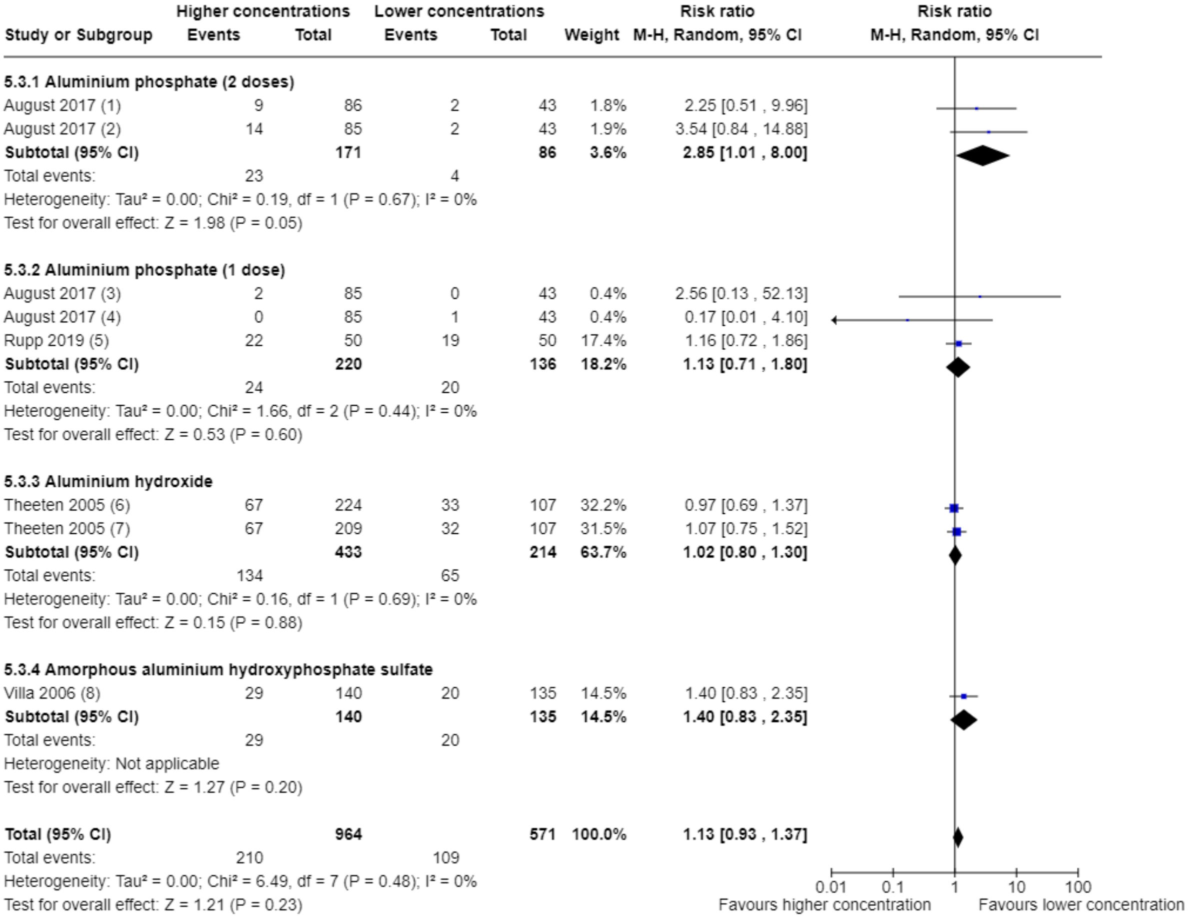

Footnotes
(1) 2-Dose; 60 µg RSV + 0.4 mg aluminium phosphate vs 2-Dose; 60 µg RSV + 0.2 mg aluminium phosphate
(2) 2-Dose; 60 µg RSV + 0.8 mg aluminium phosphate vs 2-Dose; 60 µg RSV + 0.2 mg aluminium phosphate
(3) 1-Dose; 60 µg RSV + 0.4 mg aluminium phosphate vs 1-Dose; 60 µg RSV + 0.2 mg aluminium phosphate
(4) 1-Dose; 60 µg RSV + 0.8 mg aluminium phosphate vs 1-Dose; 60 µg RSV + 0.2 mg aluminium phosphate
(5) 250 mcg aluminium phosphate vs 125 mcg aluminium phosphate
(6) 0.5 mg aluminium hydroxide vs 0.133 mg aluminium hydroxide
(7) 0.3 mg aluminium hydroxide vs 0.133 mg aluminium hydroxide
(8) 450 mcg amorphous aluminium hydroxyphosphate sulfate vs 225 mcg amorphous aluminium hydroxyphosphate sulfate

Headache

Study or Subgroup	Higher concentrations Events	Higher concentrations Total	Lower concentrations Events	Lower concentrations Total	Weight	Risk ratio M-H, Random, 95% CI
**5.4.1 Aluminium phosphate (2 doses)**						
August 2017 (1)	23	85	7	43	4.0%	1.66 [0.78 , 3.56]
August 2017 (2)	17	86	7	43	3.6%	1.21 [0.55 , 2.70]
**Subtotal (95% CI)**		**171**		**86**	**7.5%**	**1.43 [0.82 , 2.49]**
Total events:	40		14			
Heterogeneity: Tau² = 0.00; Chi² = 0.31, df = 1 (P = 0.58); I² = 0%						
Test for overall effect: Z = 1.27 (P = 0.20)						
**5.4.2 Aluminium phosphate (1 dose)**						
August 2017 (3)	11	85	8	43	3.3%	0.70 [0.30 , 1.60]
August 2017 (4)	7	85	7	43	2.4%	0.51 [0.19 , 1.35]
**Subtotal (95% CI)**		**170**		**86**	**5.7%**	**0.61 [0.32 , 1.15]**
Total events:	18		15			
Heterogeneity: Tau² = 0.00; Chi² = 0.24, df = 1 (P = 0.63); I² = 0%						
Test for overall effect: Z = 1.53 (P = 0.13)						
**5.4.3 Aluminium hydroxide**						
Theeten 2005 (5)	92	209	44	107	30.8%	1.07 [0.81 , 1.41]
Theeten 2005 (6)	101	224	44	107	31.8%	1.10 [0.84 , 1.43]
**Subtotal (95% CI)**		**433**		**214**	**62.6%**	**1.08 [0.89 , 1.31]**
Total events:	193		88			
Heterogeneity: Tau² = 0.00; Chi² = 0.02, df = 1 (P = 0.90); I² = 0%						
Test for overall effect: Z = 0.82 (P = 0.41)						
**5.4.4 Amorphous aluminium hydroxyphosphate sulfate**						
Villa 2006 (7)	54	140	48	135	24.2%	1.08 [0.80 , 1.48]
**Subtotal (95% CI)**		**140**		**135**	**24.2%**	**1.08 [0.80 , 1.48]**
Total events:	54		48			
Heterogeneity: Not applicable						
Test for overall effect: Z = 0.52 (P = 0.61)						
**Total (95% CI)**		**914**		**521**	**100.0%**	**1.07 [0.92 , 1.25]**
Total events:	305		165			
Heterogeneity: Tau² = 0.00; Chi² = 4.68, df = 6 (P = 0.59); I² = 0%						
Test for overall effect: Z = 0.89 (P = 0.37)						
Test for subgroup differences: Chi² = 4.12, df = 3 (P = 0.25), I² = 27.2%						

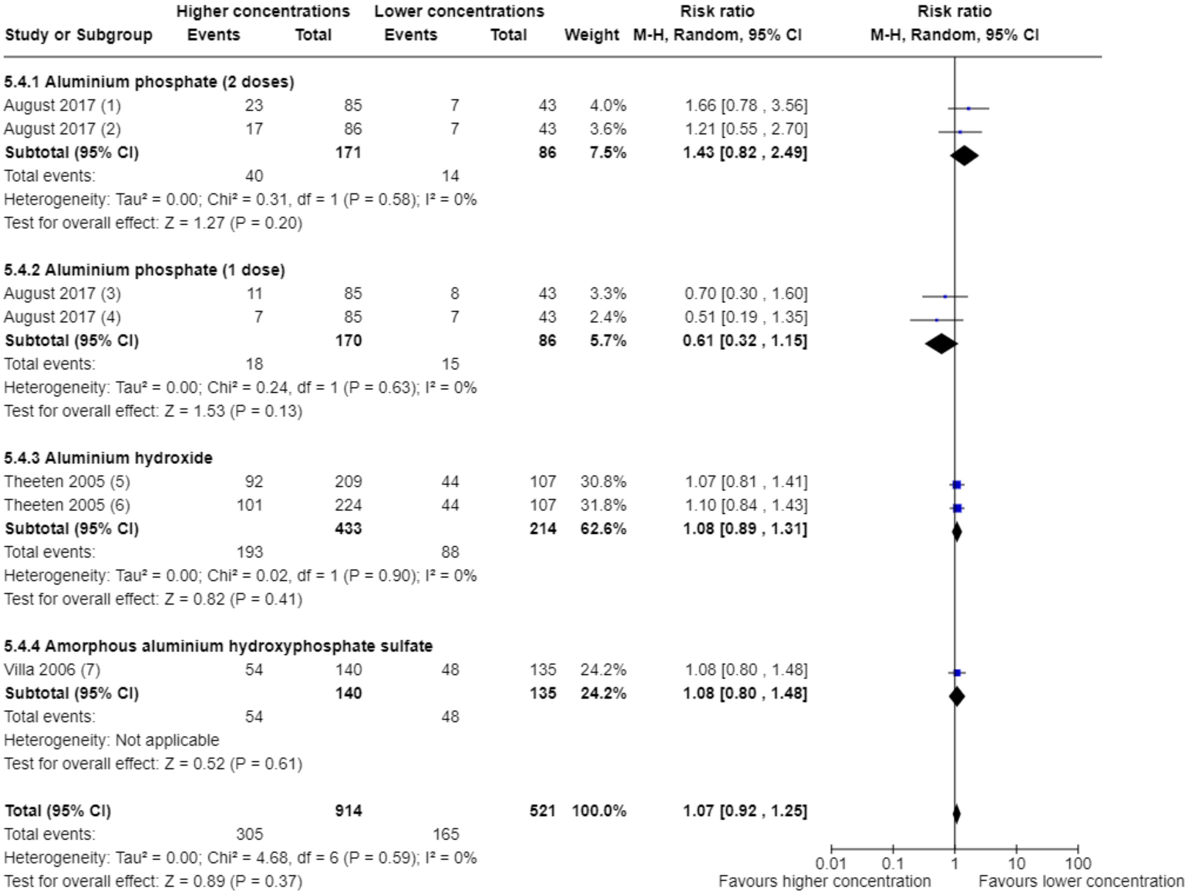

Footnotes

(1) 2-Dose; 60 µg RSV + 0.8 mg aluminium phosphate vs 2-Dose; 60 µg RSV + 0.2 mg aluminium phosphate
(2) 2-Dose; 60 µg RSV + 0.4 mg aluminium phosphate vs 2-Dose; 60 µg RSV + 0.2 mg aluminium phosphate
(3) 1-Dose; 60 µg RSV + 0.8mg aluminium phosphate vs 1-Dose; 60 µg RSV + 0.2 mg aluminium phosphate
(4) 1-Dose; 60 µg RSV + 0.4 mg aluminium phosphate vs 1-Dose; 60 µg RSV + 0.2 mg aluminium phosphate
(5) 0.3 mg aluminium hydroxide vs 0.133 mg aluminium hydroxide
(6) 0.5 mg aluminium hydroxide vs 0.133 mg aluminium hydroxide
(7) 450 mcg amorphous aluminium hydroxyphosphate sulfate vs 225 mcg amorphous aluminium hydroxyphosphate sulfate

Fatigue

Study or Subgroup	Higher concentrations Events	Higher concentrations Total	Lower concentrations Events	Lower concentrations Total	Weight	Risk ratio M-H, Random, 95% CI
**5.5.1 Aluminium phosphate (2 doses)**						
August 2017 (1)	13	85	6	43	3.8%	1.10 [0.45 , 2.68]
August 2017 (2)	14	86	1	43	0.8%	7.00 [0.95 , 51.49]
**Subtotal (95% CI)**		**171**		**86**	**4.6%**	**2.28 [0.34 , 15.21]**
Total events:	27		7			
Heterogeneity: Tau² = 1.34; Chi² = 3.16, df = 1 (P = 0.08); I² = 68%						
Test for overall effect: Z = 0.85 (P = 0.40)						
**5.5.2 Aluminium phosphate (1 dose)**						
August 2017 (3)	9	85	5	43	2.9%	0.91 [0.33 , 2.55]
August 2017 (4)	5	85	5	43	2.2%	0.51 [0.15 , 1.65]
**Subtotal (95% CI)**		**170**		**86**	**5.1%**	**0.71 [0.33 , 1.54]**
Total events:	14		10			
Heterogeneity: Tau² = 0.00; Chi² = 0.54, df = 1 (P = 0.46); I² = 0%						
Test for overall effect: Z = 0.87 (P = 0.38)						
**5.5.3 Aluminium hydroxide**						
Theeten 2005 (5)	99	224	45	107	43.0%	1.05 [0.80 , 1.37]
Theeten 2005 (6)	99	209	45	107	43.8%	1.13 [0.86 , 1.47]
**Subtotal (95% CI)**		**433**		**214**	**86.8%**	**1.09 [0.90 , 1.31]**
Total events:	198		90			
Heterogeneity: Tau² = 0.00; Chi² = 0.13, df = 1 (P = 0.72); I² = 0%						
Test for overall effect: Z = 0.88 (P = 0.38)						
**5.5.4 Amorphous aluminium hydroxyphosphate sulfate**						
Villa 2006 (7)	8	140	9	135	3.6%	0.86 [0.34 , 2.16]
**Subtotal (95% CI)**		**140**		**135**	**3.6%**	**0.86 [0.34 , 2.16]**
Total events:	8		9			
Heterogeneity: Not applicable						
Test for overall effect: Z = 0.33 (P = 0.74)						
**Total (95% CI)**		**914**		**521**	**100.0%**	**1.07 [0.90 , 1.28]**
Total events:	247		116			
Heterogeneity: Tau² = 0.00; Chi² = 5.47, df = 6 (P = 0.48); I² = 0%						
Test for overall effect: Z = 0.77 (P = 0.44)						
Test for subgroup differences: Chi² = 1.95, df = 3 (P = 0.58), I² = 0%						

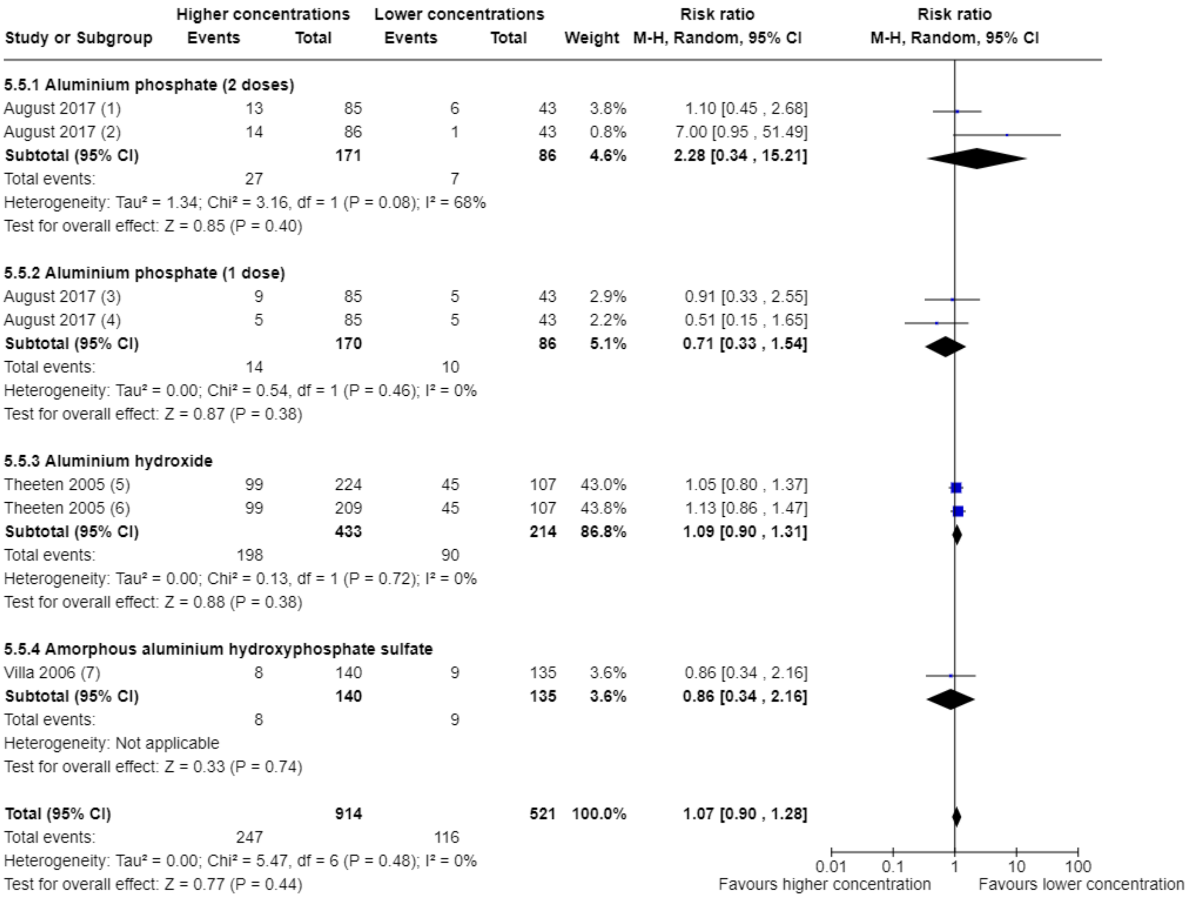

Footnotes
(1) 2-Dose; 60 µg RSV + 0.8 mg aluminium phosphate vs 2-Dose; 60 µg RSV + 0.2 mg aluminium phosphate
(2) 2-Dose; 60 µg RSV + 0.4 mg aluminium phosphate vs 2-Dose; 60 µg RSV + 0.2 mg aluminium phosphate
(3) 1-Dose; 60 µg RSV + 0.8 mg aluminium phosphate vs 1-Dose; 60 µg RSV + 0.2 mg aluminium phosphate
(4) 1-Dose; 60 µg RSV + 0.4 mg aluminium phosphate vs 1-Dose; 60 µg RSV + 0.2 mg aluminium phosphate
(5) 0.5 mg aluminium hydroxide vs 0.133 mg aluminium hydroxide
(6) 0.3 mg aluminium hydroxide vs 0.133 mg aluminium hydroxide
(7) 450 mcg amorphous aluminium hydroxyphosphate sulfate vs 225 mcg amorphous aluminium hydroxyphosphate sulfate

Gastro-intestinal

Study or Subgroup	Higher concentrations Events	Higher concentrations Total	Lower concentrations Events	Lower concentrations Total	Weight	Risk ratio M-H, Random, 95% CI
**5.6.1 Aluminium hydroxide**						
Theeten 2005 (1)	58	224	22	107	36.8%	1.26 [0.82 , 1.94]
Theeten 2005 (2)	30	209	21	107	28.8%	0.73 [0.44 , 1.21]
**Subtotal (95% CI)**		**433**		**214**	**65.6%**	**0.98 [0.57 , 1.66]**
Total events:	88		43			
Heterogeneity: Tau² = 0.09; Chi² = 2.55, df = 1 (P = 0.11); I² = 61%						
Test for overall effect: Z = 0.09 (P = 0.93)						
**5.6.2 Amorphous aluminium hydroxyphosphate sulfate**						
Villa 2006 (3)	30	140	29	135	34.4%	1.00 [0.63 , 1.57]
**Subtotal (95% CI)**		**140**		**135**	**34.4%**	**1.00 [0.63 , 1.57]**
Total events:	30		29			
Heterogeneity: Not applicable						
Test for overall effect: Z = 0.01 (P = 0.99)						
**Total (95% CI)**		**573**		**349**	**100.0%**	**0.99 [0.74 , 1.34]**
Total events:	118		72			
Heterogeneity: Tau² = 0.02; Chi² = 2.55, df = 2 (P = 0.28); I² = 22%						
Test for overall effect: Z = 0.04 (P = 0.97)						
Test for subgroup differences: Chi² = 0.00, df = 1 (P = 0.95), I² = 0%						

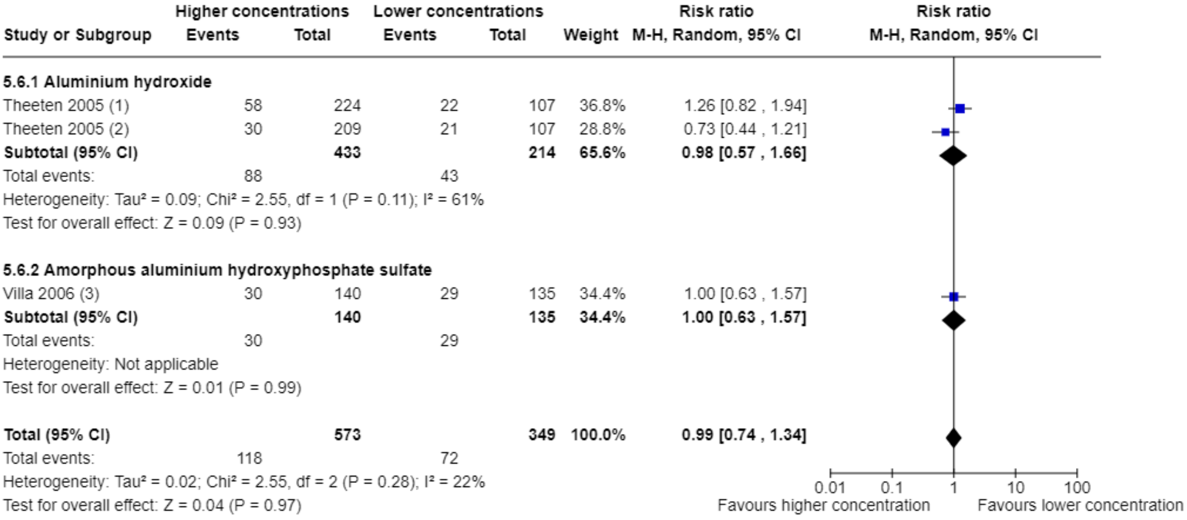

Footnotes
(1) 0.5 mg aluminium hydroxide vs 0.133 mg aluminium hydroxide
(2) 0.3 mg aluminium hydroxide vs 0.133 mg aluminium hydroxide
(3) 450 mcg amorphous aluminium hydroxyphosphate sulfate vs 225 mcg amorphous aluminium hydroxyphosphate sulfate

Fever

Study or Subgroup	Higher concentrations Events	Higher concentrations Total	Lower concentrations Events	Lower concentrations Total	Weight	Risk ratio M-H, Random, 95% CI
**5.7.1 Aluminium phosphate (2 doses)**						
August 2017 (1)	0	85	1	43	1.0%	0.17 [0.01 , 4.10]
August 2017 (2)	0	86	0	43		Not estimable
**Subtotal (95% CI)**		**171**		**86**	**1.0%**	**0.17 [0.01 , 4.10]**
Total events:	0		1			
Heterogeneity: Not applicable						
Test for overall effect: Z = 1.09 (P = 0.28)						
**5.7.2 Aluminium phosphate (1 dose)**						
August 2017 (3)	0	85	1	43	1.0%	0.17 [0.01 , 4.10]
August 2017 (4)	0	85	0	43		Not estimable
Rupp 2019 (5)	11	50	13	50	20.1%	0.85 [0.42 , 1.71]
**Subtotal (95% CI)**		**220**		**136**	**21.1%**	**0.79 [0.40 , 1.56]**
Total events:	11		14			
Heterogeneity: Tau² = 0.00; Chi² = 0.94, df = 1 (P = 0.33); I² = 0%						
Test for overall effect: Z = 0.69 (P = 0.49)						
**5.7.3 Aluminium hydroxide**						
Theeten 2005 (6)	41	224	14	107	31.3%	1.40 [0.80 , 2.45]
Theeten 2005 (7)	28	209	13	107	26.1%	1.10 [0.60 , 2.04]
**Subtotal (95% CI)**		**433**		**214**	**57.4%**	**1.26 [0.83 , 1.90]**
Total events:	69		27			
Heterogeneity: Tau² = 0.00; Chi² = 0.31, df = 1 (P = 0.58); I² = 0%						
Test for overall effect: Z = 1.08 (P = 0.28)						
**5.7.4 Amorphous aluminium hydroxyphosphate sulfate**						
Villa 2006 (8)	16	140	13	135	20.6%	1.19 [0.59 , 2.37]
**Subtotal (95% CI)**		**140**		**135**	**20.6%**	**1.19 [0.59 , 2.37]**
Total events:	16		13			
Heterogeneity: Not applicable						
Test for overall effect: Z = 0.48 (P = 0.63)						
**Total (95% CI)**		**964**		**571**	**100.0%**	**1.10 [0.81 , 1.51]**
Total events:	96		55			
Heterogeneity: Tau² = 0.00; Chi² = 3.93, df = 5 (P = 0.56); I² = 0%						
Test for overall effect: Z = 0.61 (P = 0.54)						
Test for subgroup differences: Chi² = 2.69, df = 3 (P = 0.44), I² = 0%						

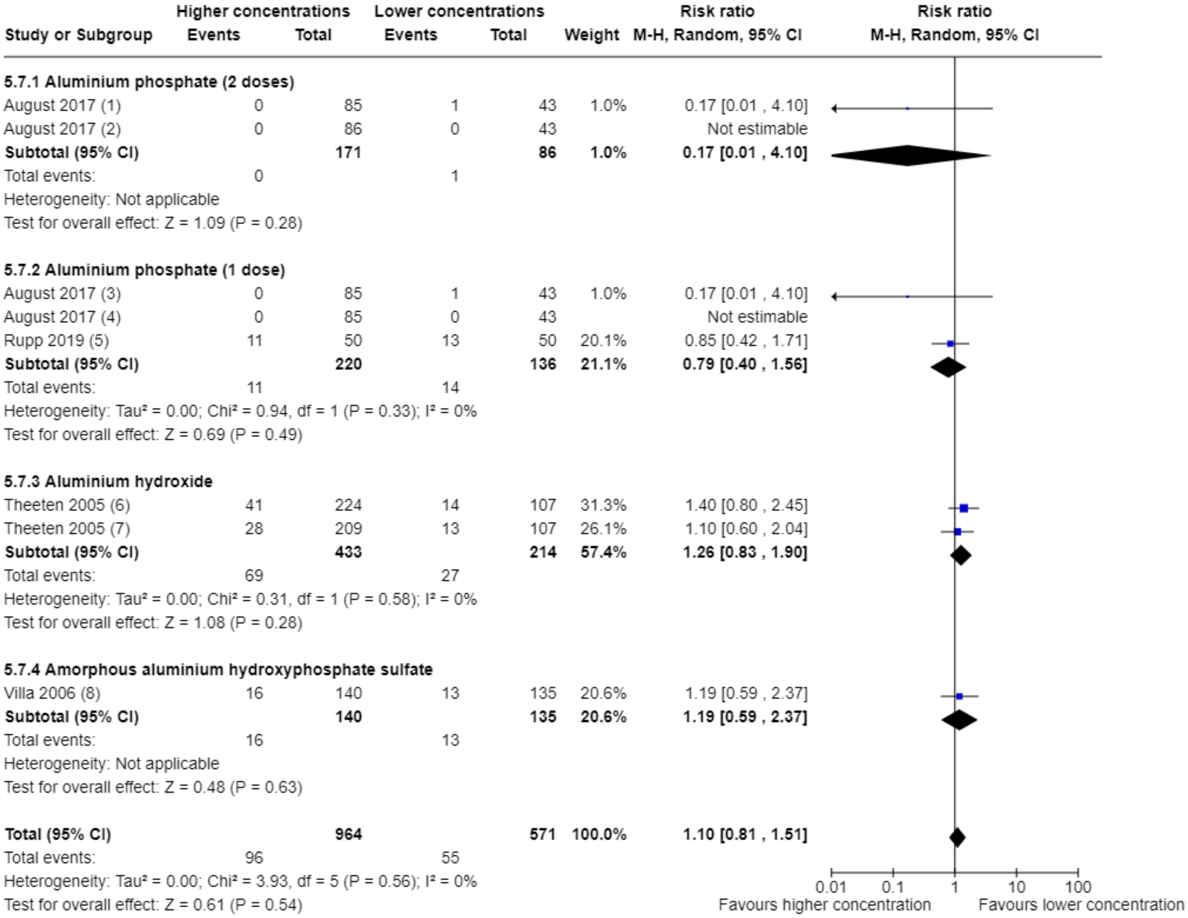

Footnotes

(1) 2-Dose; 60 µg RSV + 0.8 mg aluminium phosphate vs 2-Dose; 60 µg RSV + 0.2 mg aluminium phosphate
(2) 2-Dose; 60 µg RSV + 0.4 mg aluminium phosphate vs 2-Dose; 60 µg RSV + 0.2 mg aluminium phosphate
(3) 1-Dose; 60 µg RSV + 0.8 mg aluminium phosphate vs 1-Dose; 60 µg RSV + 0.2 mg aluminium phosphate
(4) 1-Dose; 60 µg RSV + 0.4 mg aluminium phosphate vs 1-Dose; 60 µg RSV + 0.2 mg aluminium phosphate
(5) aluminium phosphate 250 mcg Vs aluminium phosphate 125 mcg
(6) 0.5 mg aluminium hydroxide vs 0.133 mg aluminium hydroxide
(7) 0.3 mg aluminium hydroxide vs 0.133 mg aluminium hydroxide
(8) 450 mcg amorphous aluminium hydroxyphosphate sulfate vs 225 mcg amorphous aluminium hydroxyphosphate sulfate

Injection site bruising

Study or Subgroup	Higher concentrations Events	Higher concentrations Total	Lower concentrations Events	Lower concentrations Total	Weight	Risk ratio M-H, Random, 95% CI	Risk ratio M-H, Random, 95% CI
**5.8.1 Aluminium phosphate (2 doses)**							
August 2017 (1)	8	86	2	43	19.0%	2.00 [0.44 , 9.01]	
August 2017 (2)	9	85	3	43	27.4%	1.52 [0.43 , 5.32]	
**Subtotal (95% CI)**		**171**		**86**	**46.3%**	**1.70 [0.65 , 4.45]**	
Total events:	17		5				
Heterogeneity: Tau² = 0.00; Chi² = 0.08, df = 1 (P = 0.78); I² = 0%							
Test for overall effect: Z = 1.08 (P = 0.28)							
**5.8.2 Aluminium phosphate (1 dose)**							
August 2017 (3)	1	85	0	43	4.3%	1.53 [0.06 , 36.91]	
August 2017 (4)	1	85	1	43	5.7%	0.51 [0.03 , 7.89]	
Rupp 2019 (5)	3	50	4	50	20.6%	0.75 [0.18 , 3.18]	
**Subtotal (95% CI)**		**220**		**136**	**30.6%**	**0.77 [0.24 , 2.52]**	
Total events:	5		5				
Heterogeneity: Tau² = 0.00; Chi² = 0.27, df = 2 (P = 0.87); I² = 0%							
Test for overall effect: Z = 0.43 (P = 0.67)							
**5.8.3 Amorphous aluminium hydroxyphosphate sulfate**							
Villa 2006 (6)	3	140	6	135	23.1%	0.48 [0.12 , 1.89]	
**Subtotal (95% CI)**		**140**		**135**	**23.1%**	**0.48 [0.12 , 1.89]**	
Total events:	3		6				
Heterogeneity: Not applicable							
Test for overall effect: Z = 1.05 (P = 0.30)							
**Total (95% CI)**		**531**		**357**	**100.0%**	**1.00 [0.52 , 1.92]**	
Total events:	25		16				
Heterogeneity: Tau² = 0.00; Chi² = 2.80, df = 5 (P = 0.73); I² = 0%							
Test for overall effect: Z = 0.01 (P = 0.99)							
Test for subgroup differences: Chi² = 2.45, df = 2 (P = 0.29), I² = 18.2%							

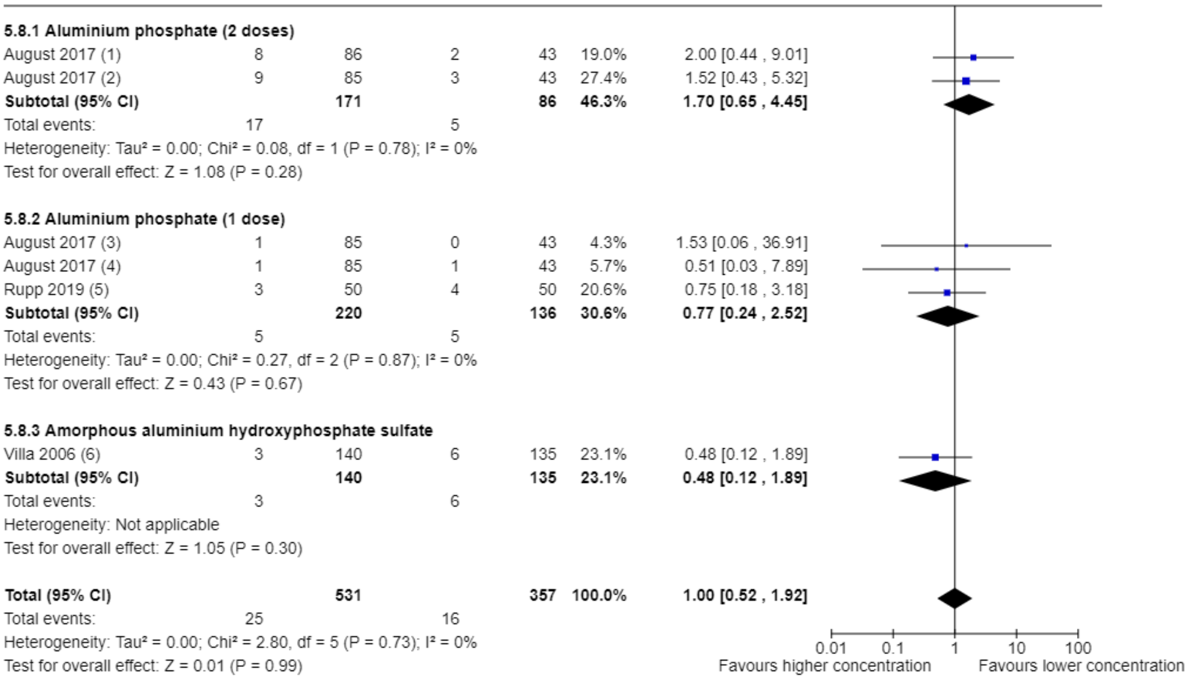

Footnotes

(1) 2-Dose; 60 µg RSV + 0.4 mg aluminium phosphate vs 2-Dose; 60 µg RSV + 0.2 mg aluminium phosphate
(2) 2-Dose; 60 µg RSV + 0.8 mg aluminium phosphate vs 2-Dose; 60 µg RSV + 0.2 mg aluminium phosphate
(3) 1-Dose; 60 µg RSV + 0.4 mg aluminium phosphate vs 1-Dose; 60 µg RSV + 0.2 mg aluminium phosphate
(4) 1-Dose; 60 µg RSV + 0.8 mg aluminium phosphate vs 1-Dose; 60 µg RSV + 0.2 mg aluminium phosphate
(5) 250 mcg aluminium phosphate vs 125 mcg aluminium phosphate
(6) 450 mcg amorphous aluminium hydroxyphosphate sulfate vs 225 mcg amorphous aluminium hydroxyphosphate sulfate

Diarrhea

Study or Subgroup	Higher concentrations Events	Total	Lower concentrations Events	Total	Weight	Risk ratio M-H, Random, 95% CI
**5.9.1 Aluminium phosphate (2 doses)**						
August 2017 (1)	2	85	2	43	14.5%	0.51 [0.07 , 3.47]
August 2017 (2)	5	86	1	43	12.0%	2.50 [0.30 , 20.74]
**Subtotal (95% CI)**		**171**		**86**	**26.5%**	**1.06 [0.22 , 5.12]**
Total events:	7		3			
Heterogeneity: Tau² = 0.24; Chi² = 1.22, df = 1 (P = 0.27); I² = 18%						
Test for overall effect: Z = 0.07 (P = 0.94)						
**5.9.2 Aluminium phosphate (1 dose)**						
August 2017 (3)	5	85	2	43	21.1%	1.26 [0.26 , 6.25]
August 2017 (4)	2	85	1	43	9.6%	1.01 [0.09 , 10.85]
Rupp 2019 (5)	6	50	5	50	42.9%	1.20 [0.39 , 3.68]
**Subtotal (95% CI)**		**220**		**136**	**73.5%**	**1.19 [0.51 , 2.80]**
Total events:	13		8			
Heterogeneity: Tau² = 0.00; Chi² = 0.02, df = 2 (P = 0.99); I² = 0%						
Test for overall effect: Z = 0.40 (P = 0.69)						
**Total (95% CI)**		**391**		**222**	**100.0%**	**1.15 [0.55 , 2.39]**
Total events:	20		11			
Heterogeneity: Tau² = 0.00; Chi² = 1.25, df = 4 (P = 0.87); I² = 0%						
Test for overall effect: Z = 0.37 (P = 0.71)						
Test for subgroup differences: Chi² = 0.02, df = 1 (P = 0.90), I² = 0%						

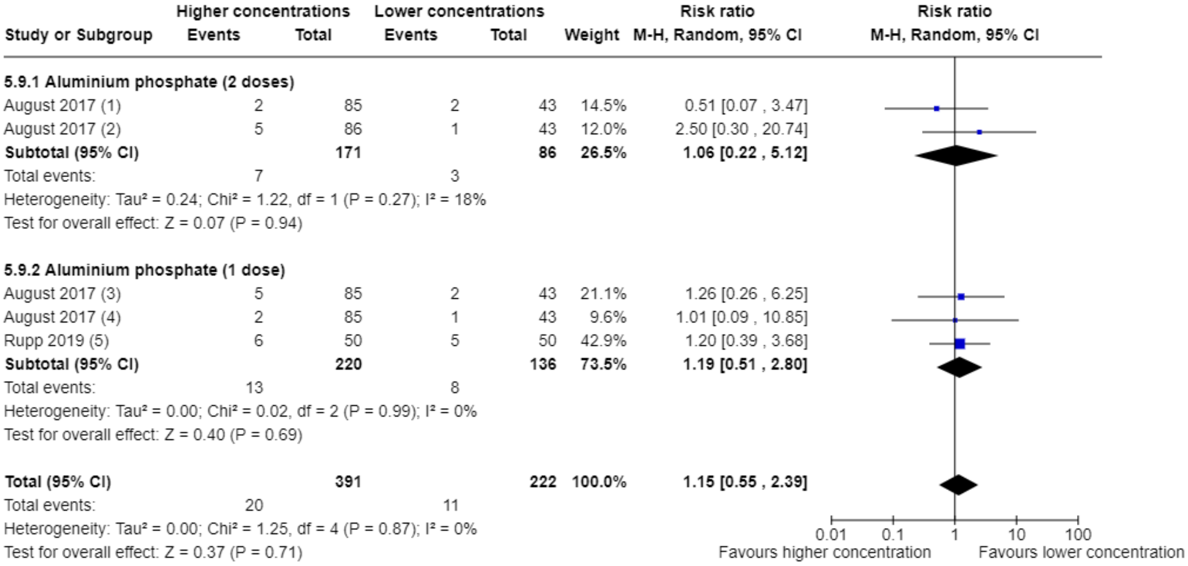

Footnotes
(1) 2-Dose; 60 µg RSV + 0.8 mg aluminium phosphate vs 2-Dose; 60 µg RSV + 0.2 mg aluminium phosphate
(2) 2-Dose; 60 µg RSV + 0.4 mg aluminium phosphate vs 2-Dose; 60 µg RSV + 0.2 mg aluminium phosphate
(3) 1-Dose; 60 µg RSV + 0.8 mg aluminium phosphate vs 1-Dose; 60 µg RSV + 0.2 mg aluminium phosphate
(4) 1-Dose; 60 µg RSV + 0.4 mg aluminium phosphate vs 1-Dose; 60 µg RSV + 0.2 mg aluminium phosphate
(5) 250 mcg aluminium phosphate vs 125 mcg aluminium phosphate

Vomiting

Study or Subgroup	Higher concentrations Events	Total	Lower concentrations Events	Total	Weight	Risk ratio M-H, Random, 95% CI
**5.10.1 Aluminium phosphate (2 doses)**						
August 2017 (1)	3	85	1	43	27.6%	1.52 [0.16 , 14.16]
August 2017 (2)	1	86	1	43	18.2%	0.50 [0.03 , 7.80]
**Subtotal (95% CI)**		**171**		**86**	**45.8%**	**0.98 [0.17 , 5.52]**
Total events:	4		2			
Heterogeneity: Tau² = 0.00; Chi² = 0.38, df = 1 (P = 0.54); I² = 0%						
Test for overall effect: Z = 0.03 (P = 0.98)						
**5.10.2 Aluminium phosphate (1 dose)**						
August 2017 (3)	0	85	0	43		Not estimable
August 2017 (4)	0	85	0	43		Not estimable
Rupp 2019 (5)	2	50	5	50	54.2%	0.40 [0.08 , 1.97]
**Subtotal (95% CI)**		**220**		**136**	**54.2%**	**0.40 [0.08 , 1.97]**
Total events:	2		5			
Heterogeneity: Not applicable						
Test for overall effect: Z = 1.13 (P = 0.26)						
**Total (95% CI)**		**391**		**222**	**100.0%**	**0.60 [0.19 , 1.94]**
Total events:	6		7			
Heterogeneity: Tau² = 0.00; Chi² = 0.93, df = 2 (P = 0.63); I² = 0%						
Test for overall effect: Z = 0.85 (P = 0.40)						
Test for subgroup differences: Chi² = 0.55, df = 1 (P = 0.46), I² = 0%						

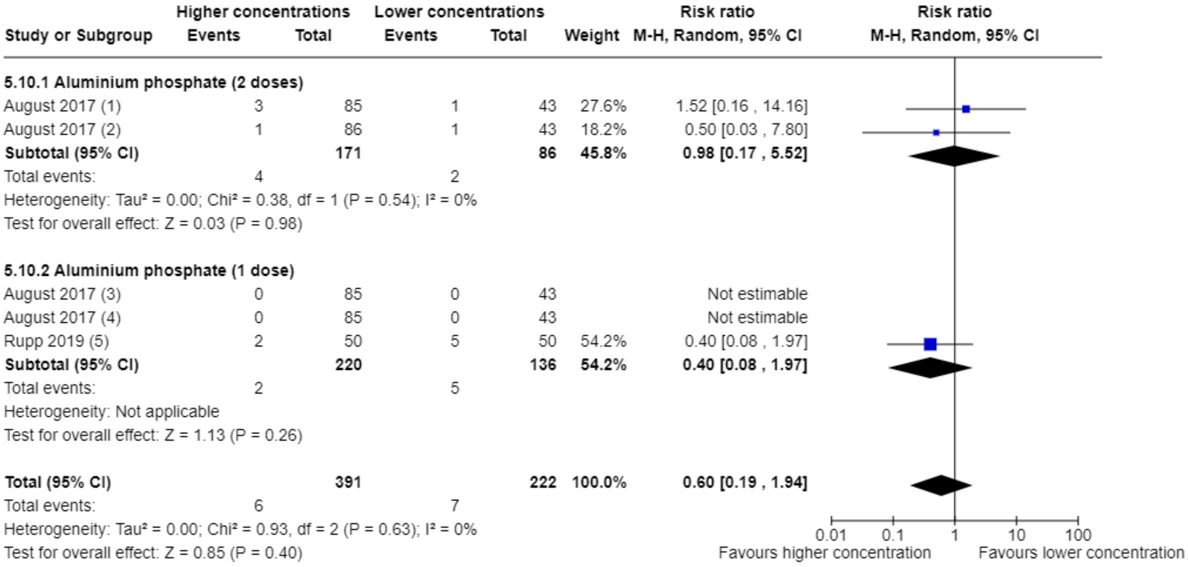

Footnotes
(1) 2-Dose; 60 µg RSV + 0.8 mg aluminium phosphate vs 2-Dose; 60 µg RSV + 0.2 mg aluminium phosphate
(2) 2-Dose; 60 µg RSV + 0.4 mg aluminium phosphate vs 2-Dose; 60 µg RSV + 0.2 mg aluminium phosphate
(3) 1-Dose; 60µg RSV+ 0.8 mg aluminium phosphate vs 1-Dose; 60 µg RSV + 0.2 mg aluminium phosphate
(4) 1-Dose; 60 µg RSV + 0.4 mg aluminium phosphate vs 1-Dose; 60 µg RSV + 0.2 mg aluminium phosphate
(5) 250 mcg aluminium phosphate vs 125 mcg aluminium phosphate

Injection site pruritus

Study or Subgroup	Higher concentrations Events	Higher concentrations Total	Lower concentrations Events	Lower concentrations Total	Weight	Risk ratio M-H, Random, 95% CI
**5.11.1 Aluminium phosphate (2 doses)**						
August 2017 (1)	4	89	0	46	15.0%	4.70 [0.26 , 85.45]
August 2017 (2)	2	91	1	46	22.4%	1.01 [0.09 , 10.86]
**Subtotal (95% CI)**		**180**		**92**	**37.4%**	**1.87 [0.30 , 11.76]**
Total events:	6		1			
Heterogeneity: Tau² = 0.00; Chi² = 0.68, df = 1 (P = 0.41); I² = 0%						
Test for overall effect: Z = 0.67 (P = 0.50)						
**5.11.2 Aluminium phosphate (1 dose)**						
August 2017 (3)	3	93	1	45	25.3%	1.45 [0.16 , 13.57]
August 2017 (4)	1	89	0	45	12.5%	1.53 [0.06 , 36.90]
**Subtotal (95% CI)**		**182**		**90**	**37.7%**	**1.48 [0.24 , 9.20]**
Total events:	4		1			
Heterogeneity: Tau² = 0.00; Chi² = 0.00, df = 1 (P = 0.98); I² = 0%						
Test for overall effect: Z = 0.42 (P = 0.68)						
**5.11.3 Amorphous aluminium hydroxyphosphate sulfate**						
Villa 2006 (5)	3	140	1	135	24.9%	2.89 [0.30 , 27.47]
**Subtotal (95% CI)**		**140**		**135**	**24.9%**	**2.89 [0.30 , 27.47]**
Total events:	3		1			
Heterogeneity: Not applicable						
Test for overall effect: Z = 0.92 (P = 0.35)						
**Total (95% CI)**		**502**		**317**	**100.0%**	**1.91 [0.62 , 5.87]**
Total events:	13		3			
Heterogeneity: Tau² = 0.00; Chi² = 0.87, df = 4 (P = 0.93); I² = 0%						
Test for overall effect: Z = 1.13 (P = 0.26)						
Test for subgroup differences: Chi² = 0.21, df = 2 (P = 0.90), I² = 0%						

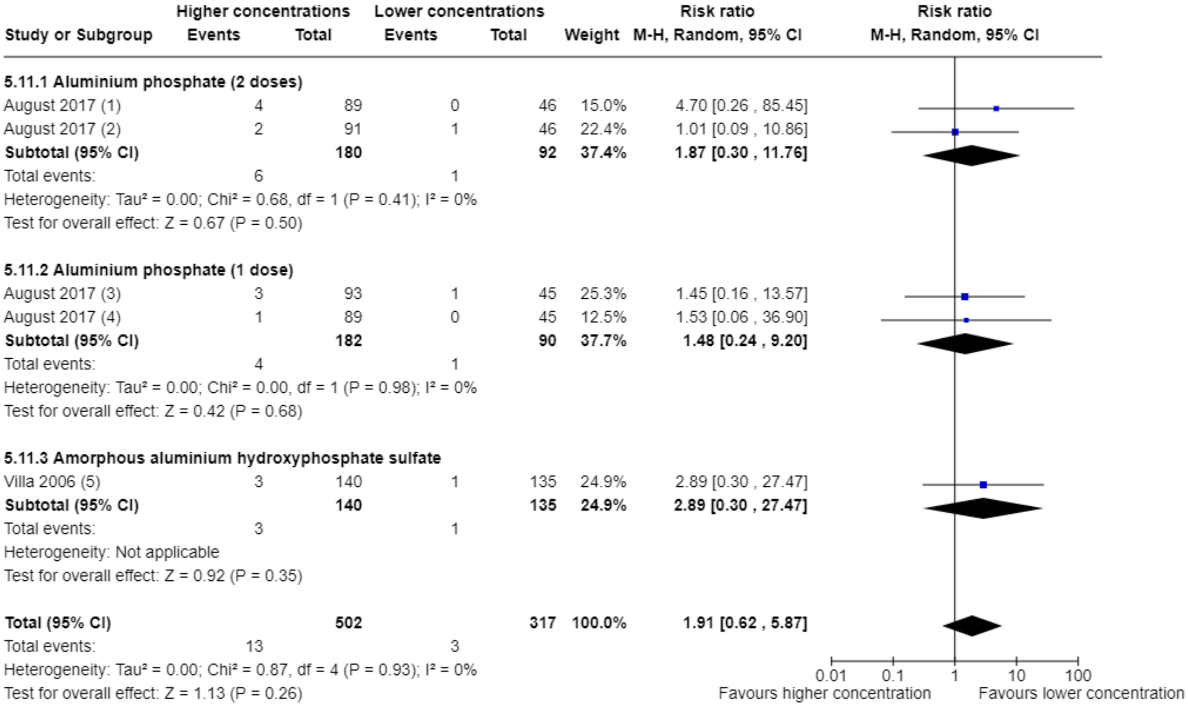

Footnotes
(1) 2-Dose; 60 µg RSV + 0.4 mg aluminium phosphate vs 2-Dose; 60 µg RSV + 0.2 mg aluminium phosphate
(2) 2-Dose; 60 µg RSV + 0.8 mg aluminium phosphate vs 2-Dose; 60 µg RSV + 0.2 mg aluminium phosphate
(3) 1-Dose; 60 µg RSV + 0.8 mg aluminium phosphate vs 1-Dose; 60 µg RSV + 0.2 mg aluminium phosphate
(4) 1-Dose; 60 µg RSV + 0.4 mg aluminium phosphate vs 1-Dose; 60 µg RSV + 0.2 mg aluminium phosphate
(5) 450 mcg amorphous aluminium hydroxyphosphate sulfate vs 225 mcg amorphous aluminium hydroxyphosphate sulfate

Nasopharyngitis

Study or Subgroup	Higher concentrations Events	Higher concentrations Total	Lower concentrations Events	Lower concentrations Total	Weight	Risk ratio M-H, Random, 95% CI
**5.12.1 Aluminium phosphate (2 doses)**						
August 2017 (1)	3	91	1	46	21.4%	1.52 [0.16 , 14.18]
August 2017 (2)	2	89	1	46	19.0%	1.03 [0.10 , 11.10]
**Subtotal (95% CI)**		**180**		**92**	**40.4%**	**1.27 [0.25 , 6.45]**
Total events:	5		2			
Heterogeneity: Tau² = 0.00; Chi² = 0.05, df = 1 (P = 0.82); I² = 0%						
Test for overall effect: Z = 0.28 (P = 0.78)						
**5.12.2 Aluminium phosphate (1 dose)**						
August 2017 (3)	1	93	2	45	19.0%	0.24 [0.02 , 2.60]
August 2017 (4)	2	89	1	45	19.0%	1.01 [0.09 , 10.86]
Rupp 2019 (5)	3	50	1	50	21.6%	3.00 [0.32 , 27.87]
**Subtotal (95% CI)**		**232**		**140**	**59.6%**	**0.94 [0.22 , 3.98]**
Total events:	6		4			
Heterogeneity: Tau² = 0.21; Chi² = 2.30, df = 2 (P = 0.32); I² = 13%						
Test for overall effect: Z = 0.08 (P = 0.94)						
**Total (95% CI)**		**412**		**232**	**100.0%**	**1.07 [0.38 , 3.00]**
Total events:	11		6			
Heterogeneity: Tau² = 0.00; Chi² = 2.43, df = 4 (P = 0.66); I² = 0%						
Test for overall effect: Z = 0.12 (P = 0.90)						
Test for subgroup differences: Chi² = 0.07, df = 1 (P = 0.79), I² = 0%						

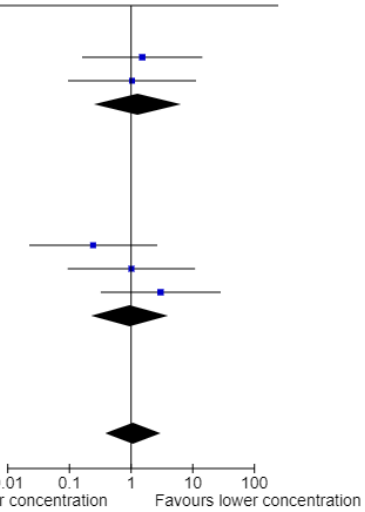

Footnotes
(1) 2-Dose; 60 µg RSV + 0.8 mg aluminium phosphate vs 2-Dose; 60 µg RSV + 0.2 mg aluminium phosphate
(2) 2-Dose; 60 µg RSV + 0.4 mg aluminium phosphate vs 2-Dose; 60 µg RSV + 0.2 mg aluminium phosphate
(3) 1-Dose; 60 µg RSV + 0.8 mg aluminium phosphate vs 1-Dose; 60 µg RSV + 0.2 mg aluminium phosphate
(4) 1-Dose; 60 µg RSV + 0.4 mg aluminium phosphate vs 1-Dose; 60 µg RSV + 0.2 mg aluminium phosphate
(5) 250 mcg aluminium phosphate vs 125 mcg aluminium phosphate

Sinusitis

Study or Subgroup	Higher concentrations Events	Higher concentrations Total	Lower concentrations Events	Lower concentrations Total	Weight	Risk ratio M-H, Random, 95% CI
**5.13.1 Aluminium phosphate (2 doses)**						
August 2017 (1)	1	89	0	46	18.7%	1.57 [0.07 , 37.72]
August 2017 (2)	4	91	1	46	40.6%	2.02 [0.23 , 17.58]
**Subtotal (95% CI)**		**180**		**92**	**59.3%**	**1.87 [0.31 , 11.15]**
Total events:	5		1			
Heterogeneity: Tau² = 0.00; Chi² = 0.02, df = 1 (P = 0.90); I² = 0%						
Test for overall effect: Z = 0.68 (P = 0.49)						
**5.13.2 Aluminium phosphate (1 dose)**						
August 2017 (3)	3	93	0	45	21.9%	3.43 [0.18 , 64.93]
August 2017 (4)	0	89	0	45		Not estimable
Rupp 2019 (5)	0	50	1	50	18.8%	0.33 [0.01 , 7.99]
**Subtotal (95% CI)**		**232**		**140**	**40.7%**	**1.16 [0.12 , 11.38]**
Total events:	3		1			
Heterogeneity: Tau² = 0.29; Chi² = 1.12, df = 1 (P = 0.29); I² = 11%						
Test for overall effect: Z = 0.13 (P = 0.90)						
**Total (95% CI)**		**412**		**232**	**100.0%**	**1.54 [0.39 , 6.11]**
Total events:	8		2			
Heterogeneity: Tau² = 0.00; Chi² = 1.24, df = 3 (P = 0.74); I² = 0%						
Test for overall effect: Z = 0.62 (P = 0.54)						
Test for subgroup differences: Chi² = 0.10, df = 1 (P = 0.75), I² = 0%						

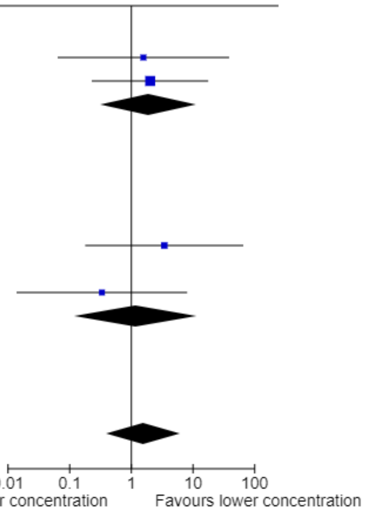

Footnotes
(1) 2-Dose; 60 µg RSV + 0.4 mg aluminium phosphate vs 2-Dose; 60 µg RSV + 0.2 mg aluminium phosphate
(2) 2-Dose; 60 µg RSV + 0.8 mg aluminium phosphate vs 2-Dose; 60 µg RSV + 0.2 mg aluminium phosphate
(3) 1-Dose; 60 µg RSV + 0.8 mg aluminium phosphate vs 1-Dose; 60 µg RSV + 0.2 mg aluminium phosphate
(4) 1-Dose; 60 µg RSV + 0.4 mg aluminium phosphate vs 1-Dose; 60 µg RSV + 0.2 mg aluminium phosphate
(5) 250 mcg aluminium phosphate vs 125 mcg aluminium phosphate

Upper respiratory tract infection

Study or Subgroup	Higher concentrations Events	Higher concentrations Total	Lower concentrations Events	Lower concentrations Total	Weight	Risk ratio M-H, Random, 95% CI
**5.14.1 Aluminium phosphate (2 doses)**						
August 2017 (1)	14	91	6	46	26.2%	1.18 [0.49 , 2.87]
August 2017 (2)	10	89	6	46	23.0%	0.86 [0.33 , 2.22]
**Subtotal (95% CI)**		**180**		**92**	**49.2%**	**1.02 [0.53 , 1.95]**
Total events:	24		12			
Heterogeneity: Tau² = 0.00; Chi² = 0.22, df = 1 (P = 0.64); I² = 0%						
Test for overall effect: Z = 0.05 (P = 0.96)						
**5.14.2 Aluminium phosphate (1 dose)**						
August 2017 (3)	10	93	5	45	20.1%	0.97 [0.35 , 2.66]
August 2017 (4)	13	89	5	45	22.1%	1.31 [0.50 , 3.46]
Rupp 2019 (5)	3	50	3	50	8.6%	1.00 [0.21 , 4.72]
**Subtotal (95% CI)**		**232**		**140**	**50.8%**	**1.11 [0.59 , 2.10]**
Total events:	26		13			
Heterogeneity: Tau² = 0.00; Chi² = 0.21, df = 2 (P = 0.90); I² = 0%						
Test for overall effect: Z = 0.33 (P = 0.74)						
**Total (95% CI)**		**412**		**232**	**100.0%**	**1.06 [0.68 , 1.68]**
Total events:	50		25			
Heterogeneity: Tau² = 0.00; Chi² = 0.47, df = 4 (P = 0.98); I² = 0%						
Test for overall effect: Z = 0.27 (P = 0.79)						
Test for subgroup differences: Chi² = 0.04, df = 1 (P = 0.85), I² = 0%						

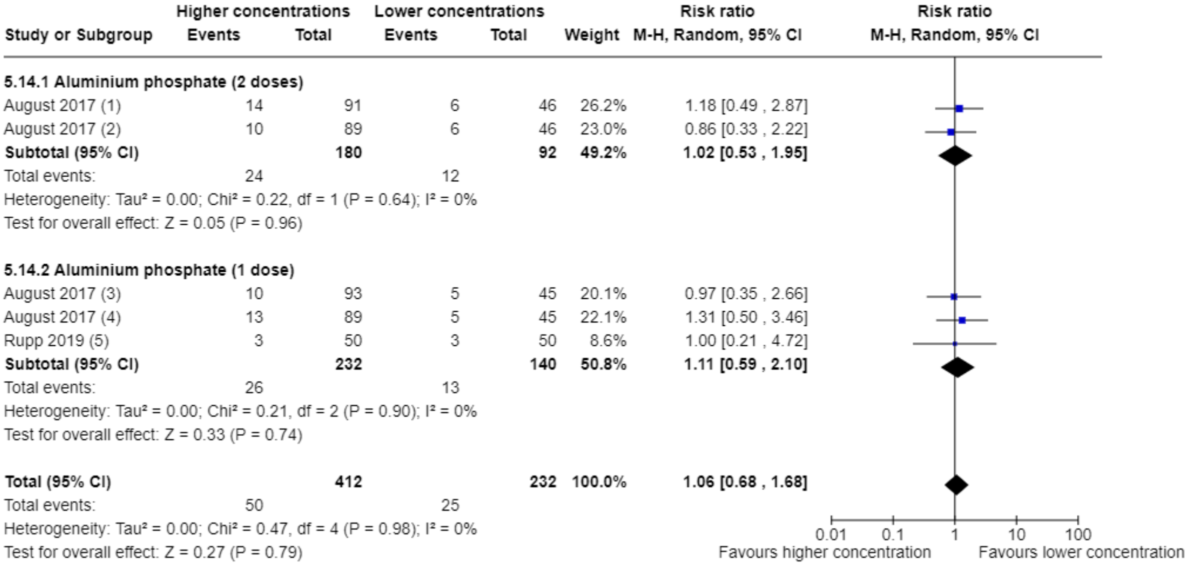

Footnotes
(1) 2-Dose; 60 µg RSV + 0.8 mg aluminium phosphate vs 2-Dose; 60 µg RSV + 0.2 mg aluminium phosphate
(2) 2-Dose; 60 µg RSV + 0.4 mg aluminium phosphate vs 2-Dose; 60 µg RSV + 0.2 mg aluminium phosphate
(3) 1-Dose; 60 µg RSV + 0.8 mg aluminium phosphate vs 1-Dose; 60 µg RSV + 0.2 mg aluminium phosphate
(4) 1-Dose; 60 µg RSV + 0.4 mg aluminium phosphate vs 1-Dose; 60 µg RSV + 0.2 mg aluminium phosphate
(5) 250 mcg aluminium phosphate vs 125 mcg aluminium phosphate

Viral infection

Study or Subgroup	Higher concentrations Events	Higher concentrations Total	Lower concentrations Events	Lower concentrations Total	Weight	Risk ratio M-H, Random, 95% CI
**5.15.1 Aluminium phosphate (2 doses)**						
August 2017 (1)	5	89	0	46	19.3%	5.74 [0.32 , 101.67]
August 2017 (2)	1	91	1	46	21.0%	0.51 [0.03 , 7.90]
**Subtotal (95% CI)**		**180**		**92**	**40.3%**	**1.65 [0.14 , 19.37]**
Total events:	6		1			
Heterogeneity: Tau² = 1.11; Chi² = 1.54, df = 1 (P = 0.21); I² = 35%						
Test for overall effect: Z = 0.40 (P = 0.69)						
**5.15.2 Aluminium phosphate (1 dose)**						
August 2017 (3)	2	93	1	45	28.2%	0.97 [0.09 , 10.39]
August 2017 (4)	1	89	0	45	15.7%	1.53 [0.06 , 36.90]
Rupp 2019 (5)	1	50	0	50	15.8%	3.00 [0.13 , 71.92]
**Subtotal (95% CI)**		**232**		**140**	**59.7%**	**1.47 [0.29 , 7.53]**
Total events:	4		1			
Heterogeneity: Tau² = 0.00; Chi² = 0.32, df = 2 (P = 0.85); I² = 0%						
Test for overall effect: Z = 0.46 (P = 0.64)						
**Total (95% CI)**		**412**		**232**	**100.0%**	**1.53 [0.43 , 5.39]**
Total events:	10		2			
Heterogeneity: Tau² = 0.00; Chi² = 1.84, df = 4 (P = 0.77); I² = 0%						
Test for overall effect: Z = 0.66 (P = 0.51)						
Test for subgroup differences: Chi² = 0.01, df = 1 (P = 0.94), I² = 0%						

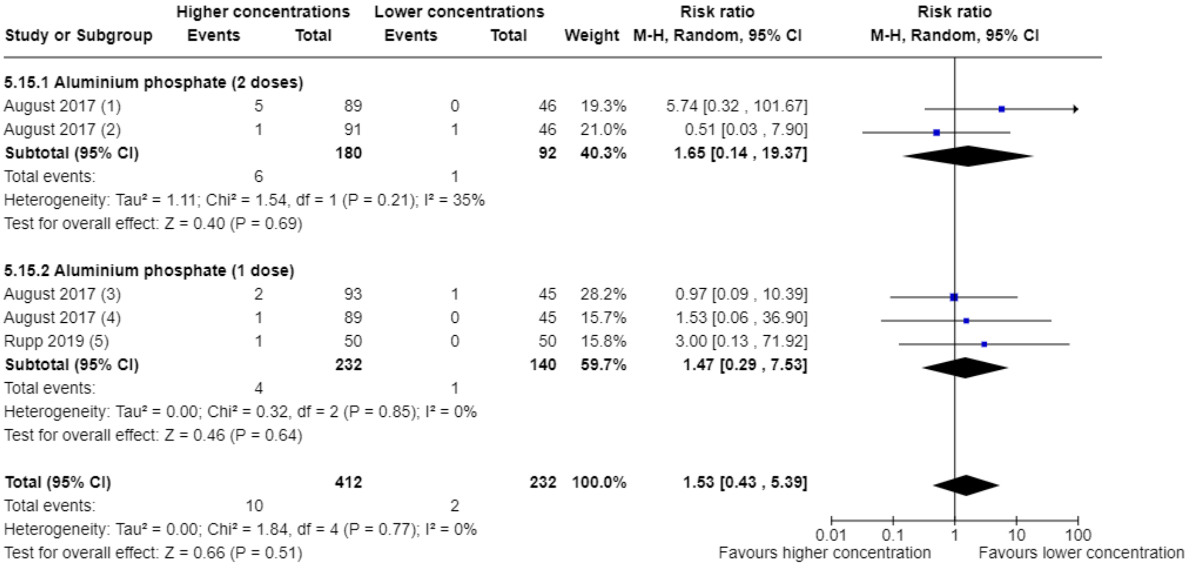

Footnotes
(1) 2-Dose; 60 µg RSV + 0.4 mg aluminium phosphate vs 2-Dose; 60 µg RSV + 0.2 mg aluminium phosphate
(2) 2-Dose; 60 µg RSV + 0.8 mg aluminium phosphate vs 2-Dose; 60 µg RSV + 0.2 mg aluminium phosphate
(3) 1-Dose; 60 µg RSV + 0.8 mg aluminium phosphate vs 1-Dose; 60 µg RSV + 0.2 mg aluminium phosphate
(4) 1-Dose; 60 µg RSV + 0.4 mg aluminium phosphate vs 1-Dose; 60 µg RSV + 0.2 mg aluminium phosphate
(5) 250 mcg aluminium phosphate vs 125 mcg aluminium phosphate

Viral upper respiratory tract infection

Study or Subgroup	Higher concentrations Events	Higher concentrations Total	Lower concentrations Events	Lower concentrations Total	Weight	Risk ratio M-H, Random, 95% CI
**5.16.1 Aluminium phosphate (2 doses)**						
August 2017 (1)	2	91	1	46	35.9%	1.01 [0.09 , 10.86]
August 2017 (2)	1	89	0	46	20.0%	1.57 [0.07 , 37.72]
**Subtotal (95% CI)**		**180**		**92**	**55.9%**	**1.18 [0.18 , 7.93]**
Total events:	3		1			
Heterogeneity: Tau² = 0.00; Chi² = 0.05, df = 1 (P = 0.83); I² = 0%						
Test for overall effect: Z = 0.17 (P = 0.86)						
**5.16.2 Aluminium phosphate (1 dose)**						
August 2017 (3)	4	93	0	45	24.1%	4.40 [0.24 , 80.07]
August 2017 (4)	0	89	0	45		Not estimable
Rupp 2019 (5)	1	50	0	50	20.0%	3.00 [0.13 , 71.92]
**Subtotal (95% CI)**		**232**		**140**	**44.1%**	**3.70 [0.43 , 31.50]**
Total events:	5		0			
Heterogeneity: Tau² = 0.00; Chi² = 0.03, df = 1 (P = 0.86); I² = 0%						
Test for overall effect: Z = 1.20 (P = 0.23)						
**Total (95% CI)**		**412**		**232**	**100.0%**	**1.96 [0.47 , 8.11]**
Total events:	8		1			
Heterogeneity: Tau² = 0.00; Chi² = 0.71, df = 3 (P = 0.87); I² = 0%						
Test for overall effect: Z = 0.92 (P = 0.36)						
Test for subgroup differences: Chi² = 0.61, df = 1 (P = 0.44), I² = 0%						

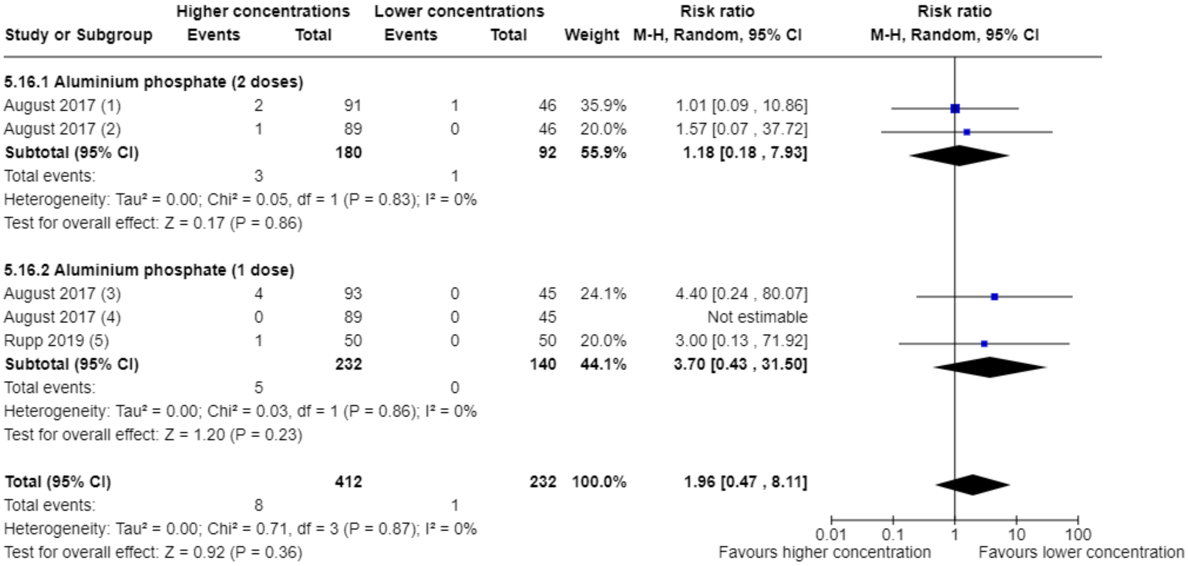

Footnotes
(1) 2-Dose; 60 µg RSV + 0.8 mg aluminium phosphate vs 2-Dose; 60 µg RSV + 0.2 mg aluminium phosphate
(2) 2-Dose; 60 µg RSV + 0.4 mg aluminium phosphate vs 2-Dose; 60 µg RSV + 0.2 mg aluminium phosphate
(3) 1-Dose; 60 µg RSV + 0.8 mg aluminium phosphate vs 1-Dose; 60 µg RSV + 0.2 mg aluminium phosphate
(4) 1-Dose; 60 µg RSV + 0.4 mg aluminium phosphate vs 1-Dose; 60 µg RSV + 0.2 mg aluminium phosphate
(5) 250 mcg aluminium phosphate vs 125 mcg aluminium phosphate

Decreased appetite

Study or Subgroup	Higher concentrations Events	Higher concentrations Total	Lower concentrations Events	Lower concentrations Total	Weight	Risk ratio M-H, Random, 95% CI
**5.17.1 Aluminium phosphate (2 doses)**						
August 2017 (1)	1	91	0	46	1.5%	1.53 [0.06 , 36.90]
August 2017 (2)	0	89	0	46		Not estimable
**Subtotal (95% CI)**		**180**		**92**	**1.5%**	**1.53 [0.06 , 36.90]**
Total events:	1		0			
Heterogeneity: Not applicable						
Test for overall effect: Z = 0.26 (P = 0.79)						
**5.17.2 Aluminium phosphate (1 dose)**						
August 2017 (3)	2	93	0	45	1.7%	2.45 [0.12 , 49.93]
August 2017 (4)	0	89	0	45		Not estimable
Rupp 2019 (5)	26	50	23	50	96.7%	1.13 [0.76 , 1.69]
**Subtotal (95% CI)**		**232**		**140**	**98.5%**	**1.15 [0.77 , 1.71]**
Total events:	28		23			
Heterogeneity: Tau² = 0.00; Chi² = 0.26, df = 1 (P = 0.61); I² = 0%						
Test for overall effect: Z = 0.67 (P = 0.50)						
**Total (95% CI)**		**412**		**232**	**100.0%**	**1.15 [0.78 , 1.71]**
Total events:	29		23			
Heterogeneity: Tau² = 0.00; Chi² = 0.29, df = 2 (P = 0.86); I² = 0%						
Test for overall effect: Z = 0.70 (P = 0.49)						
Test for subgroup differences: Chi² = 0.03, df = 1 (P = 0.86), I² = 0%						

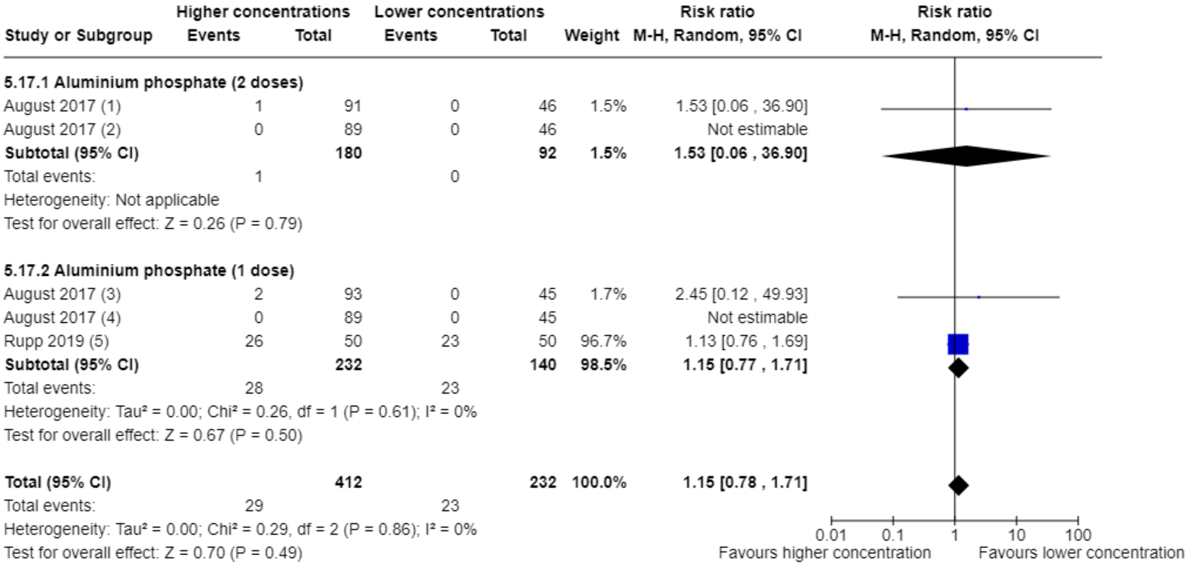

Footnotes
(1) 2-Dose; 60 µg RSV + 0.8 mg aluminium phosphate vs 2-Dose; 60 µg RSV + 0.2 mg aluminium phosphate
(2) 2-Dose; 60 µg RSV + 0.4 mg aluminium phosphate vs 2-Dose; 60 µg RSV + 0.2 mg aluminium phosphate
(3) 1-Dose; 60 µg RSV + 0.8 mg aluminium phosphate vs 1-Dose; 60 µg RSV + 0.2 mg aluminium phosphate
(4) 1-Dose; 60 µg RSV + 0.4 mg aluminium phosphate vs 1-Dose; 60 µg RSV + 0.2 mg aluminium phosphate
(5) 250 mcg aluminium phosphate vs 125 mcg aluminium phosphate

Dizziness

Study or Subgroup	Higher concentrations Events	Higher concentrations Total	Lower concentrations Events	Lower concentrations Total	Weight	Risk ratio M-H, Random, 95% CI
**5.18.1 Aluminium phosphate (2 doses)**						
August 2017 (1)	1	91	0	46	15.6%	1.53 [0.06 , 36.90]
August 2017 (2)	1	89	0	46	15.6%	1.57 [0.07 , 37.72]
**Subtotal (95% CI)**		**180**		**92**	**31.3%**	**1.55 [0.16 , 14.69]**
Total events:	2		0			
Heterogeneity: Tau² = 0.00; Chi² = 0.00, df = 1 (P = 0.99); I² = 0%						
Test for overall effect: Z = 0.38 (P = 0.70)						
**5.18.2 Aluminium phosphate (1 dose)**						
August 2017 (3)	1	93	0	45	15.6%	1.47 [0.06 , 35.34]
August 2017 (4)	3	89	0	45	18.3%	3.58 [0.19 , 67.80]
**Subtotal (95% CI)**		**182**		**90**	**33.9%**	**2.37 [0.27 , 20.57]**
Total events:	4		0			
Heterogeneity: Tau² = 0.00; Chi² = 0.17, df = 1 (P = 0.68); I² = 0%						
Test for overall effect: Z = 0.78 (P = 0.43)						
**5.18.3 Amorphous aluminium hydroxyphosphate sulfate**						
Villa 2006 (5)	5	140	1	135	34.8%	4.82 [0.57 , 40.73]
**Subtotal (95% CI)**		**140**		**135**	**34.8%**	**4.82 [0.57 , 40.73]**
Total events:	5		1			
Heterogeneity: Not applicable						
Test for overall effect: Z = 1.44 (P = 0.15)						
**Total (95% CI)**		**502**		**317**	**100.0%**	**2.66 [0.76 , 9.35]**
Total events:	11		1			
Heterogeneity: Tau² = 0.00; Chi² = 0.70, df = 4 (P = 0.95); I² = 0%						
Test for overall effect: Z = 1.52 (P = 0.13)						
Test for subgroup differences: Chi² = 0.53, df = 2 (P = 0.77), I² = 0%						

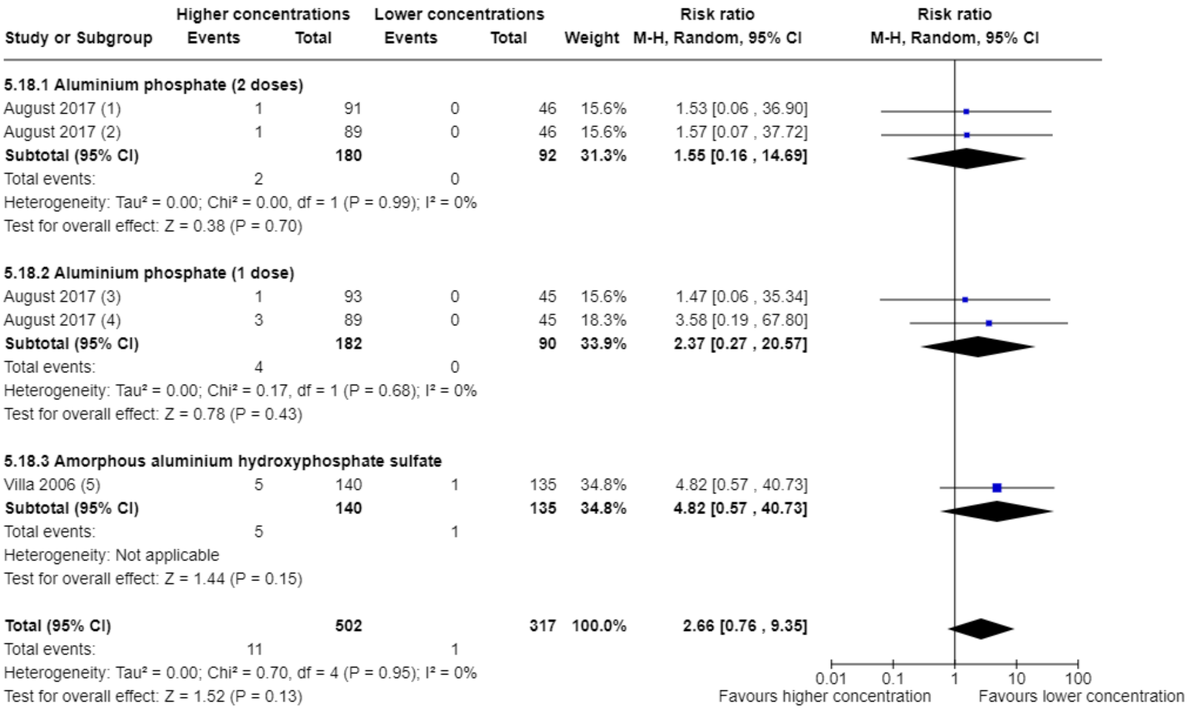

Footnotes

(1) 2-Dose; 60 µg RSV + 0.8 mg aluminium phosphate vs 2-Dose; 60 µg RSV + 0.2 mg aluminium phosphate
(2) 2-Dose; 60 µg RSV + 0.4 mg aluminium phosphate vs 2-Dose; 60 µg RSV + 0.2 mg aluminium phosphate
(3) 1-Dose; 60 µg RSV + 0.8 mg aluminium phosphate vs 1-Dose; 60 µg RSV + 0.2 mg aluminium phosphate
(4) 1-Dose; 60 µg RSV + 0.4 mg aluminium phosphate vs 1-Dose; 60 µg RSV + 0.2 mg aluminium phosphate
(5) 450 mcg amorphous aluminium hydroxyphosphate sulfate vs 225 mcg amorphous aluminium hydroxyphosphate sulfate

Insomnia

Study or Subgroup	Higher concentrations Events	Higher concentrations Total	Lower concentrations Events	Lower concentrations Total	Weight	Risk ratio M-H, Random, 95% CI
**5.19.1 Aluminium phosphate (2 doses)**						
August 2017 (1)	0	91	1	46	18.7%	0.17 [0.01 , 4.10]
August 2017 (2)	1	89	1	46	25.1%	0.52 [0.03 , 8.08]
**Subtotal (95% CI)**		**180**		**92**	**43.8%**	**0.32 [0.04 , 2.57]**
Total events:	1		2			
Heterogeneity: Tau² = 0.00; Chi² = 0.27, df = 1 (P = 0.60); I² = 0%						
Test for overall effect: Z = 1.07 (P = 0.29)						
**5.19.2 Aluminium phosphate (1 dose)**						
August 2017 (3)	0	93	1	45	18.7%	0.16 [0.01 , 3.93]
August 2017 (4)	1	89	0	45	18.7%	1.53 [0.06 , 36.90]
Rupp 2019 (5)	0	50	1	50	18.8%	0.33 [0.01 , 7.99]
**Subtotal (95% CI)**		**232**		**140**	**56.2%**	**0.44 [0.07 , 2.74]**
Total events:	1		2			
Heterogeneity: Tau² = 0.00; Chi² = 0.99, df = 2 (P = 0.61); I² = 0%						
Test for overall effect: Z = 0.88 (P = 0.38)						
**Total (95% CI)**		**412**		**232**	**100.0%**	**0.38 [0.10 , 1.51]**
Total events:	2		4			
Heterogeneity: Tau² = 0.00; Chi² = 1.31, df = 4 (P = 0.86); I² = 0%						
Test for overall effect: Z = 1.37 (P = 0.17)						
Test for subgroup differences: Chi² = 0.05, df = 1 (P = 0.83), I² = 0%						

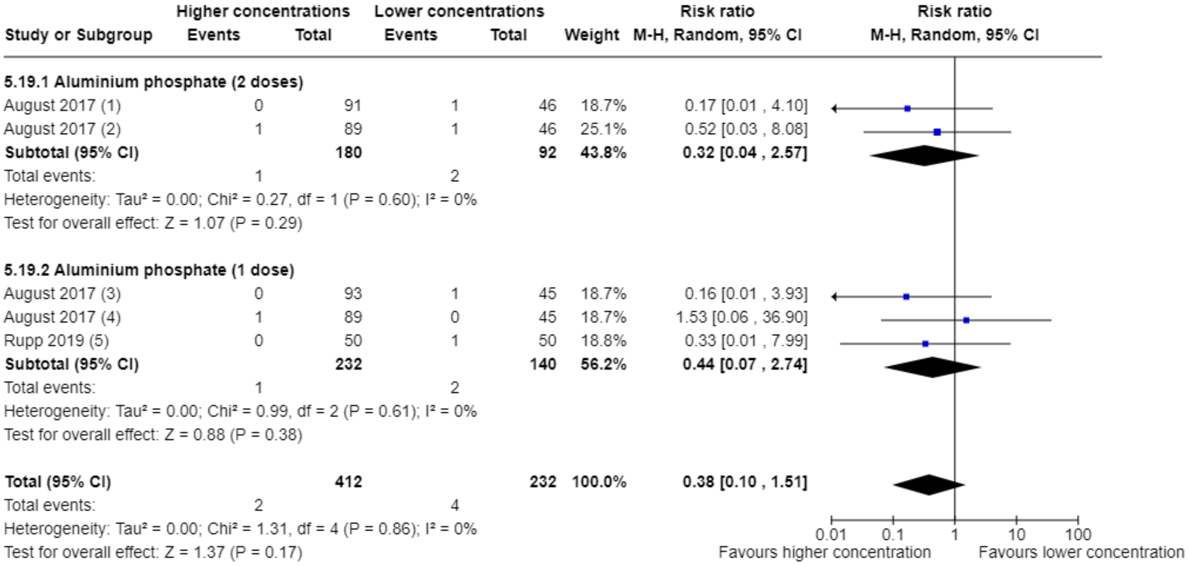

Footnotes
(1) 2-Dose; 60 µg RSV + 0.8 mg aluminium phosphate vs 2-Dose; 60 µg RSV + 0.2 mg aluminium phosphate
(2) 2-Dose; 60 µg RSV + 0.4 mg aluminium phosphate vs 2-Dose; 60 µg RSV + 0.2 mg aluminium phosphate
(3) 1-Dose; 60 µg RSV + 0.8 mg aluminium phosphate vs 1-Dose; 60 µg RSV + 0.2 mg aluminium phosphate
(4) 1-Dose; 60 µg RSV + 0.4 mg aluminium phosphate vs 1-Dose; 60 µg RSV + 0.2 mg aluminium phosphate
(5) 250 mcg aluminium phosphate vs 125 mcg aluminium phosphate

Cough

Study or Subgroup	Higher concentrations Events	Higher concentrations Total	Lower concentrations Events	Lower concentrations Total	Weight	Risk ratio M-H, Random, 95% CI
**5.20.1 Aluminium phosphate (2 doses)**						
August 2017 (1)	6	91	3	46	26.2%	1.01 [0.26 , 3.86]
August 2017 (2)	3	89	2	46	15.3%	0.78 [0.13 , 4.48]
**Subtotal (95% CI)**		**180**		**92**	**41.5%**	**0.92 [0.32 , 2.66]**
Total events:	9		5			
Heterogeneity: Tau² = 0.00; Chi² = 0.06, df = 1 (P = 0.81); I² = 0%						
Test for overall effect: Z = 0.16 (P = 0.87)						
**5.20.2 Aluminium phosphate (1 dose)**						
August 2017 (3)	7	93	3	45	27.6%	1.13 [0.31 , 4.16]
August 2017 (4)	1	89	2	45	8.3%	0.25 [0.02 , 2.71]
Rupp 2019 (5)	3	50	4	50	22.5%	0.75 [0.18 , 3.18]
**Subtotal (95% CI)**		**232**		**140**	**58.5%**	**0.78 [0.32 , 1.91]**
Total events:	11		9			
Heterogeneity: Tau² = 0.00; Chi² = 1.18, df = 2 (P = 0.55); I² = 0%						
Test for overall effect: Z = 0.55 (P = 0.59)						
**Total (95% CI)**		**412**		**232**	**100.0%**	**0.83 [0.42 , 1.65]**
Total events:	20		14			
Heterogeneity: Tau² = 0.00; Chi² = 1.29, df = 4 (P = 0.86); I² = 0%						
Test for overall effect: Z = 0.52 (P = 0.60)						
Test for subgroup differences: Chi² = 0.05, df = 1 (P = 0.82), I² = 0%						

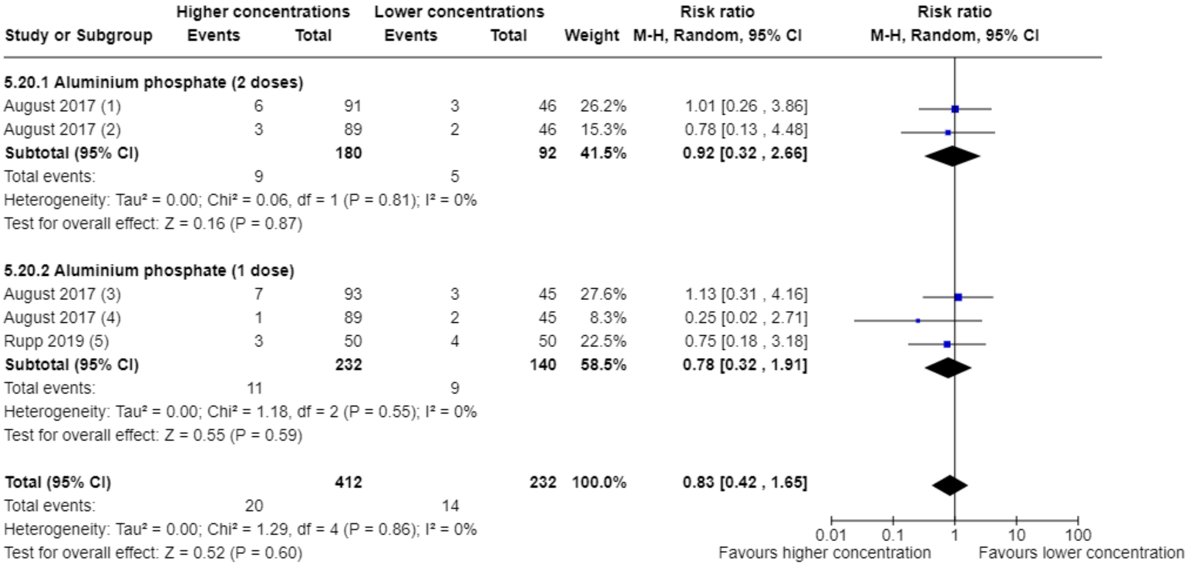

Footnotes
(1) 2-Dose; 60 µg RSV + 0.8 mg aluminium phosphate vs 2-Dose; 60 µg RSV + 0.2 mg aluminium phosphate
(2) 2-Dose; 60 µg RSV + 0.4 mg aluminium phosphate vs 2-Dose; 60 µg RSV + 0.2 mg aluminium phosphate
(3) 1-Dose; 60 µg RSV + 0.8 mg aluminium phosphate vs 1-Dose; 60 µg RSV + 0.2 mg aluminium phosphate
(4) 1-Dose; 60 µg RSV + 0.4 mg aluminium phosphate vs 1-Dose; 60 µg RSV + 0.2 mg aluminium phosphate
(5) 250 mcg aluminium phosphate vs 125 mcg aluminium phosphate

Nasal congestion

Study or Subgroup	Higher concentrations Events	Higher concentrations Total	Lower concentrations Events	Lower concentrations Total	Weight	Risk ratio M-H, Random, 95% CI
**5.21.1 Aluminium phosphate (2 doses)**						
August 2017 (1)	5	91	3	46	27.3%	0.84 [0.21 , 3.37]
August 2017 (2)	3	89	3	46	21.5%	0.52 [0.11 , 2.46]
**Subtotal (95% CI)**		**180**		**92**	**48.8%**	**0.68 [0.24 , 1.91]**
Total events:	8		6			
Heterogeneity: Tau² = 0.00; Chi² = 0.21, df = 1 (P = 0.65); I² = 0%						
Test for overall effect: Z = 0.73 (P = 0.46)						
**5.21.2 Aluminium phosphate (1 dose)**						
August 2017 (3)	4	93	3	45	24.8%	0.65 [0.15 , 2.76]
August 2017 (4)	2	89	3	45	17.1%	0.34 [0.06 , 1.95]
Rupp 2019 (5)	1	50	2	50	9.3%	0.50 [0.05 , 5.34]
**Subtotal (95% CI)**		**232**		**140**	**51.2%**	**0.50 [0.18 , 1.36]**
Total events:	7		8			
Heterogeneity: Tau² = 0.00; Chi² = 0.31, df = 2 (P = 0.86); I² = 0%						
Test for overall effect: Z = 1.36 (P = 0.17)						
**Total (95% CI)**		**412**		**232**	**100.0%**	**0.58 [0.28 , 1.19]**
Total events:	15		14			
Heterogeneity: Tau² = 0.00; Chi² = 0.70, df = 4 (P = 0.95); I² = 0%						
Test for overall effect: Z = 1.48 (P = 0.14)						
Test for subgroup differences: Chi² = 0.18, df = 1 (P = 0.67), I² = 0%						

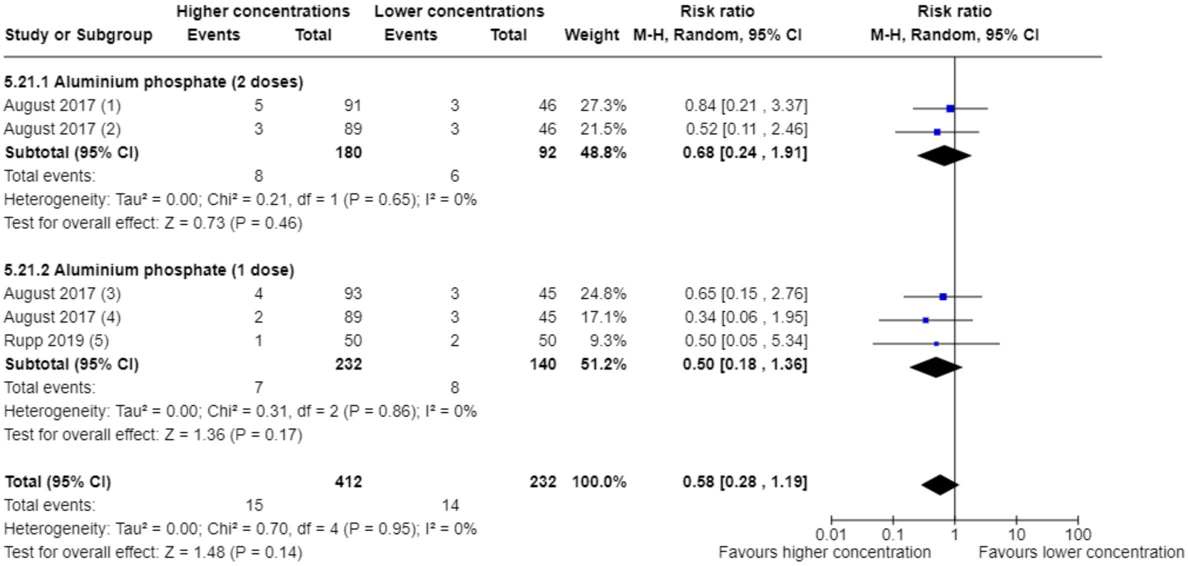

Footnotes
(1) 2-Dose; 60 µg RSV + 0.8 mg aluminium phosphate vs 2-Dose; 60 µg RSV + 0.2 mg aluminium phosphate
(2) 2-Dose; 60 µg RSV + 0.4 mg aluminium phosphate vs 2-Dose; 60 µg RSV + 0.2 mg aluminium phosphate
(3) 1-Dose; 60 µg RSV + 0.8 mg aluminium phosphate vs 1-Dose; 60 µg RSV + 0.2 mg aluminium phosphate
(4) 1-Dose; 60 µg RSV + 0.4 mg aluminium phosphate vs 1-Dose; 60 µg RSV + 0.2 mg aluminium phosphate
(5) 250 mcg aluminium phosphate vs 125 mcg aluminium phosphate

Oropharyngeal pain

Study or Subgroup	Higher concentrations Events	Higher concentrations Total	Lower concentrations Events	Lower concentrations Total	Weight	Risk ratio M-H, Random, 95% CI
**5.22.1 Aluminium phosphate (2 doses)**						
August 2017 (1)	8	89	1	46	16.3%	4.13 [0.53 , 32.06]
August 2017 (2)	5	91	2	46	26.7%	1.26 [0.25 , 6.27]
**Subtotal (95% CI)**		**180**		**92**	**42.9%**	**1.98 [0.56 , 6.99]**
Total events:	13		3			
Heterogeneity: Tau² = 0.00; Chi² = 0.83, df = 1 (P = 0.36); I² = 0%						
Test for overall effect: Z = 1.06 (P = 0.29)						
**5.22.2 Aluminium phosphate (1 dose)**						
August 2017 (3)	6	93	2	45	28.1%	1.45 [0.30 , 6.91]
August 2017 (4)	3	89	2	45	22.2%	0.76 [0.13 , 4.38]
Rupp 2019 (5)	1	50	0	50	6.8%	3.00 [0.13 , 71.92]
**Subtotal (95% CI)**		**232**		**140**	**57.1%**	**1.23 [0.41 , 3.67]**
Total events:	10		4			
Heterogeneity: Tau² = 0.00; Chi² = 0.64, df = 2 (P = 0.73); I² = 0%						
Test for overall effect: Z = 0.37 (P = 0.71)						
**Total (95% CI)**		**412**		**232**	**100.0%**	**1.51 [0.66 , 3.45]**
Total events:	23		7			
Heterogeneity: Tau² = 0.00; Chi² = 1.80, df = 4 (P = 0.77); I² = 0%						
Test for overall effect: Z = 0.97 (P = 0.33)						
Test for subgroup differences: Chi² = 0.31, df = 1 (P = 0.57), I² = 0%						

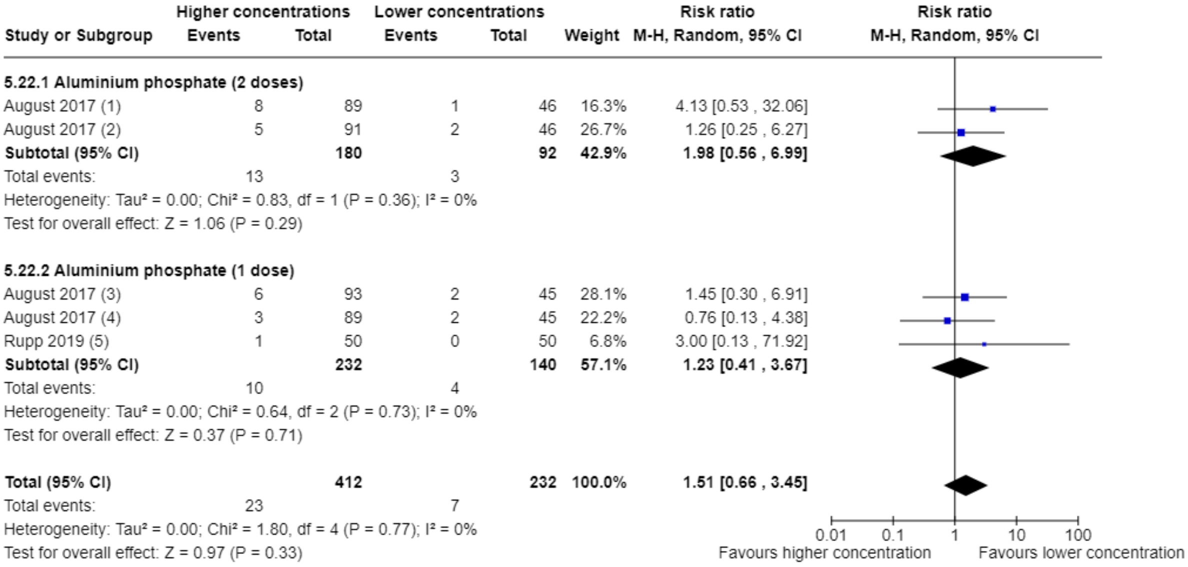

Footnotes
(1) 2-Dose; 60 µg RSV + 0.4 mg aluminium phosphate vs 2-Dose; 60 µg RSV + 0.2 mg aluminium phosphate
(2) 2-Dose; 60 µg RSV + 0.8 mg aluminium phosphate vs 2-Dose; 60 µg RSV + 0.2 mg aluminium phosphate
(3) 1-Dose; 60 µg RSV + 0.8 mg aluminium phosphate vs 1-Dose; 60 µg RSV + 0.2 mg aluminium phosphate
(4) 1-Dose; 60 µg RSV + 0.4 mg aluminium phosphate vs 1-Dose; 60 µg RSV + 0.2 mg aluminium phosphate
(5) 250 mcg aluminium phosphate vs 125 mcg aluminium phosphate

Rhinorrhea

Study or Subgroup	Higher concentrations Events	Higher concentrations Total	Lower concentrations Events	Lower concentrations Total	Weight	Risk ratio M-H, Random, 95% CI
**5.23.1 Aluminium phosphate (2 doses)**						
August 2017 (1)	0	89	2	46	9.4%	0.10 [0.01 , 2.13]
August 2017 (2)	5	91	3	46	44.7%	0.84 [0.21 , 3.37]
**Subtotal (95% CI)**		**180**		**92**	**54.1%**	**0.46 [0.07 , 3.03]**
Total events:	5		5			
Heterogeneity: Tau² = 0.81; Chi² = 1.57, df = 1 (P = 0.21); I² = 36%						
Test for overall effect: Z = 0.81 (P = 0.42)						
**5.23.2 Aluminium phosphate (1 dose)**						
August 2017 (3)	3	93	1	45	17.2%	1.45 [0.16 , 13.57]
August 2017 (4)	1	89	1	45	11.4%	0.51 [0.03 , 7.90]
Rupp 2019 (5)	1	50	3	50	17.3%	0.33 [0.04 , 3.10]
**Subtotal (95% CI)**		**232**		**140**	**45.9%**	**0.64 [0.16 , 2.52]**
Total events:	5		5			
Heterogeneity: Tau² = 0.00; Chi² = 0.87, df = 2 (P = 0.65); I² = 0%						
Test for overall effect: Z = 0.64 (P = 0.53)						
**Total (95% CI)**		**412**		**232**	**100.0%**	**0.61 [0.24 , 1.54]**
Total events:	10		10			
Heterogeneity: Tau² = 0.00; Chi² = 2.43, df = 4 (P = 0.66); I² = 0%						
Test for overall effect: Z = 1.04 (P = 0.30)						
Test for subgroup differences: Chi² = 0.08, df = 1 (P = 0.78), I² = 0%						

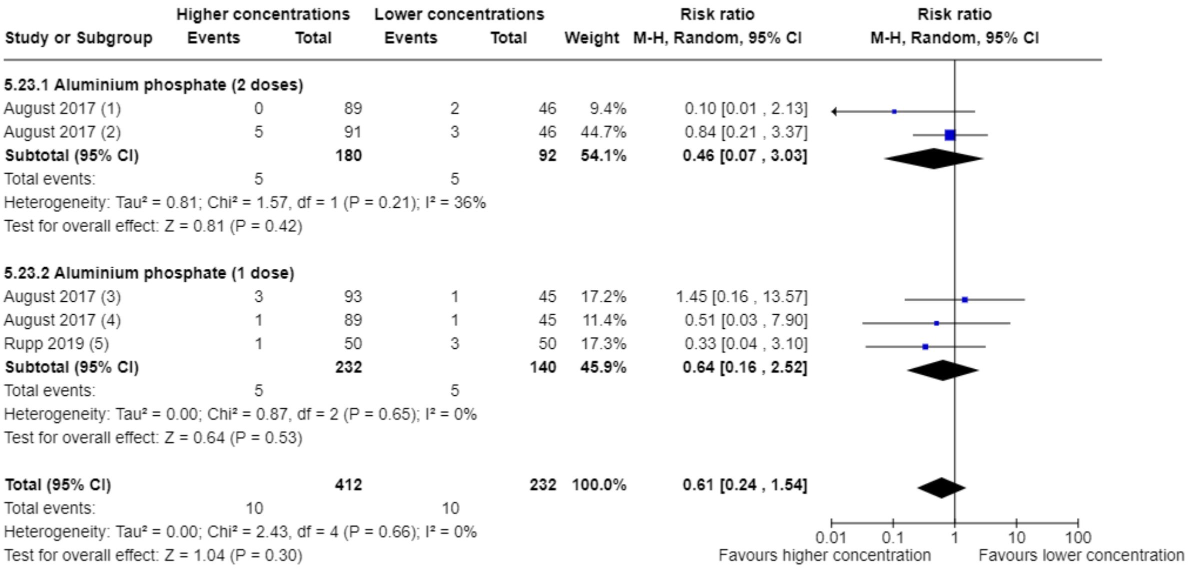

Footnotes
(1) 2-Dose; 60 µg RSV + 0.4 mg aluminium phosphate vs 2-Dose; 60 µg RSV + 0.2 mg aluminium phosphate
(2) 2-Dose; 60 µg RSV + 0.8 mg aluminium phosphate vs 2-Dose; 60 µg RSV + 0.2 mg aluminium phosphate
(3) 1-Dose; 60 µg RSV + 0.8 mg aluminium phosphate vs 1-Dose; 60 µg RSV + 0.2 mg aluminium phosphate
(4) 1-Dose; 60 µg RSV + 0.4 mg aluminium phosphate vs 1-Dose; 60 µg RSV + 0.2 mg aluminium phosphate
(5) 250 mcg aluminium phosphate vs 125 mcg aluminium phosphate

Rash

Study or Subgroup	Higher concentrations Events	Higher concentrations Total	Lower concentrations Events	Lower concentrations Total	Weight	Risk ratio M-H, Random, 95% CI
**5.24.1 Aluminium phosphate**						
Rupp 2019 (1)	0	50	3	50	44.6%	0.14 [0.01 , 2.70]
**Subtotal (95% CI)**		**50**		**50**	**44.6%**	**0.14 [0.01 , 2.70]**
Total events:	0		3			
Heterogeneity: Not applicable						
Test for overall effect: Z = 1.30 (P = 0.19)						
**5.24.2 Amorphous aluminium hydroxyphosphate sulfate**						
Villa 2006 (2)	2	140	1	135	55.4%	1.93 [0.18 , 21.02]
**Subtotal (95% CI)**		**140**		**135**	**55.4%**	**1.93 [0.18 , 21.02]**
Total events:	2		1			
Heterogeneity: Not applicable						
Test for overall effect: Z = 0.54 (P = 0.59)						
**Total (95% CI)**		**190**		**185**	**100.0%**	**0.60 [0.05 , 7.95]**
Total events:	2		4			
Heterogeneity: Tau² = 1.63; Chi² = 1.87, df = 1 (P = 0.17); I² = 47%						
Test for overall effect: Z = 0.38 (P = 0.70)						
Test for subgroup differences: Chi² = 1.82, df = 1 (P = 0.18), I² = 44.9%						

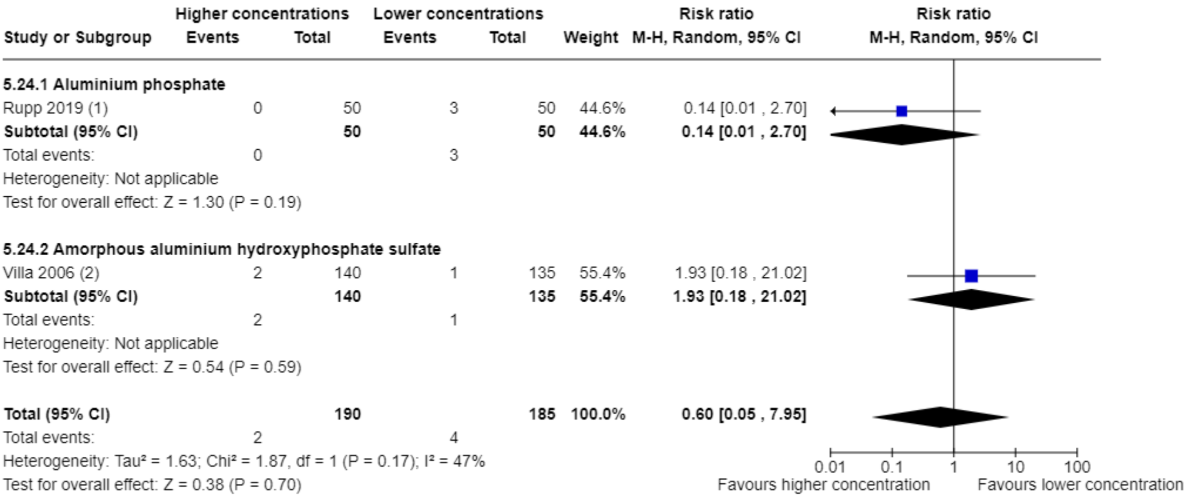

Footnotes
(1) 250 mcg aluminium phosphate vs 125 mcg aluminium phosphate
(2) 450 mcg amorphous aluminium hydroxyphosphate sulfate vs 225 mcg amorphous aluminium hydroxyphosphate sulfate

Injection site erythema

Study or Subgroup	Higher concentrations Events	Higher concentrations Total	Lower concentrations Events	Lower concentrations Total	Weight	Risk ratio M-H, Random, 95% CI
**5.25.1 Aluminium phosphate**						
Rupp 2019 (1)	28	50	22	50	58.2%	1.27 [0.86 , 1.89]
**Subtotal (95% CI)**		**50**		**50**	**58.2%**	**1.27 [0.86 , 1.89]**
Total events:	28		22			
Heterogeneity: Not applicable						
Test for overall effect: Z = 1.19 (P = 0.23)						
**5.25.2 Amorphous aluminium hydroxyphosphate sulfate**						
Villa 2006 (2)	30	140	26	135	41.8%	1.11 [0.70 , 1.78]
**Subtotal (95% CI)**		**140**		**135**	**41.8%**	**1.11 [0.70 , 1.78]**
Total events:	30		26			
Heterogeneity: Not applicable						
Test for overall effect: Z = 0.45 (P = 0.66)						
**Total (95% CI)**		**190**		**185**	**100.0%**	**1.20 [0.89 , 1.63]**
Total events:	58		48			
Heterogeneity: Tau² = 0.00; Chi² = 0.19, df = 1 (P = 0.66); I² = 0%						
Test for overall effect: Z = 1.20 (P = 0.23)						
Test for subgroup differences: Chi² = 0.18, df = 1 (P = 0.67), I² = 0%						

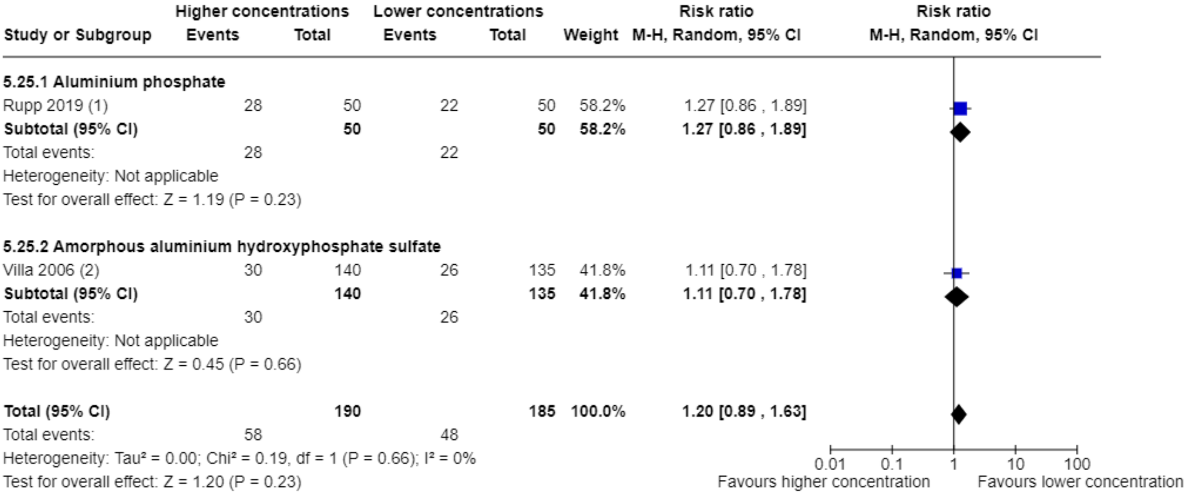

Footnotes
(1) 250 mcg aluminium phosphate vs 125 mcg aluminium phosphate
(2) 450 mcg amorphous aluminium hydroxyphosphate sulfate vs 225 mcg morphous aluminium hydroxyphosphate sulfate

81 different types of adverse events not considered serious were reported by one trial

Adverse event	Trial
Mucsle pain	August 2017
Nausea	August 2017
Joint pain	August 2017
Chills	August 2017
Anaemia	August 2017
Abdominal pain	August 2017
Abdominal pain upper	August 2017
Influenzae-like illness	August 2017
Gastroenteritis	August 2017
Gastroenteritis viral	August 2017
Influenzae	August 2017
Pharyngitis	August 2017
Pharyngitis streptococal	August 2017
Tonsilitis	August 2017
Urinary tract infection	August 2017
Ligament strain	August 2017
Alanine aminitransferase increased	August 2017
Blood pressure diastolic increased	August 2017
Haemoglobin decreased	August 2017
Respiratory rate increased	August 2017
Arthralgia	August 2017
Back pain	August 2017
Myalgia	August 2017
Neck pain	August 2017
Dysmenorrhea	August 2017
Throat irritation	August 2017
Injection site reaction	Villa 2006
Plagiocephaly	Rupp 2019
Cerumen impaction	Rupp 2019
Ear pain	Rupp 2019
Dacryostenosis acquired	Rupp 2019
Eye discharge	Rupp 2019
Lacrimation increased	Rupp 2019
Constipation	Rupp 2019
Flatulence	Rupp 2019
Gastrooesophageal reflux disease	Rupp 2019
Infantile spitting up	Rupp 2019
Stomatitis	Rupp 2019
Teething	Rupp 2019
Injection site induration	Rupp 2019
Injection site macule	Rupp 2019

Injection site nodule	Rupp 2019
Pain	Rupp 2019
Bronchiolitis	Rupp 2019
Candida infection	Rupp 2019
Croup infectious	Rupp 2019
Hand-foot-and-mouth disease	Rupp 2019
Impetigo	Rupp 2019
Injection site abscess	Rupp 2019
Lower respiratory tract infection	Rupp 2019
Metapneumovirus infection	Rupp 2019
Oral candidiasis	Rupp 2019
Otitis media	Rupp 2019
Otitis media acute	Rupp 2019
Otitis media chronic	Rupp 2019
Pharyngitis streptococcal	Rupp 2019
Respiratory syncytial virus bronchiolitis	Rupp 2019
Rhinitis	Rupp 2019
Viral rash	Rupp 2019
Accidental overdose	Rupp 2019
Arthropod bite	Rupp 2019
Body temperature increased	Rupp 2019
Stool pH decreased	Rupp 2019
Abnormal weight gain	Rupp 2019
Cow's milk intolerance	Rupp 2019
Pain in extremity	Rupp 2019
Haemangioma	Rupp 2019
Somnolence	Rupp 2019
Abnormal behaviour	Rupp 2019
Irritability	Rupp 2019
Respiratory disorder	Rupp 2019
Sneezing	Rupp 2019
Dermatitis atopic	Rupp 2019
Dermatitis contact	Rupp 2019
Dermatitis diaper	Rupp 2019
Eczema	Rupp 2019
Macule	Rupp 2019
Rash generalised	Rupp 2019
Seborrhoea	Rupp 2019
Seborrhoeic dermatitis	Rupp 2019
Urticaria	Rupp 2019

Serological response

Study or Subgroup	Higher concentration Mean	SD	Total	Lower concentration Mean	SD	Total	Weight	Mean difference IV, Random, 95% CI
**1.7.1 Geometric mean concentration**								
Goubau 1992 (1)	-9	1.6	37	-8.7	2	37	55.1%	-0.30 [-1.13 , 0.53]
Rupp 2019 (2)	-0.5	1.6	29	-0.5	2	31	44.9%	0.00 [-0.91 , 0.91]
**Subtotal (95% CI)**			**66**			**68**	**100.0%**	**-0.17 [-0.78 , 0.45]**
Heterogeneity: Tau² = 0.00; Chi² = 0.23, df = 1 (P = 0.63); I² = 0%								
Test for overall effect: Z = 0.53 (P = 0.60)								
**1.7.2 Geometric mean titer**								
Theeten 2005 (3)	-8.38	1.65	217	-8.52	1.9	105	53.4%	0.14 [-0.28 , 0.56]
Theeten 2005 (4)	-8.45	1.97	201	-8.52	1.9	105	46.6%	0.07 [-0.38 , 0.52]
**Subtotal (95% CI)**			**418**			**210**	**100.0%**	**0.11 [-0.20 , 0.42]**
Heterogeneity: Tau² = 0.00; Chi² = 0.05, df = 1 (P = 0.83); I² = 0%								
Test for overall effect: Z = 0.68 (P = 0.50)								
Test for subgroup differences: Chi² = 0.61, df = 1 (P = 0.44), I² = 0%								

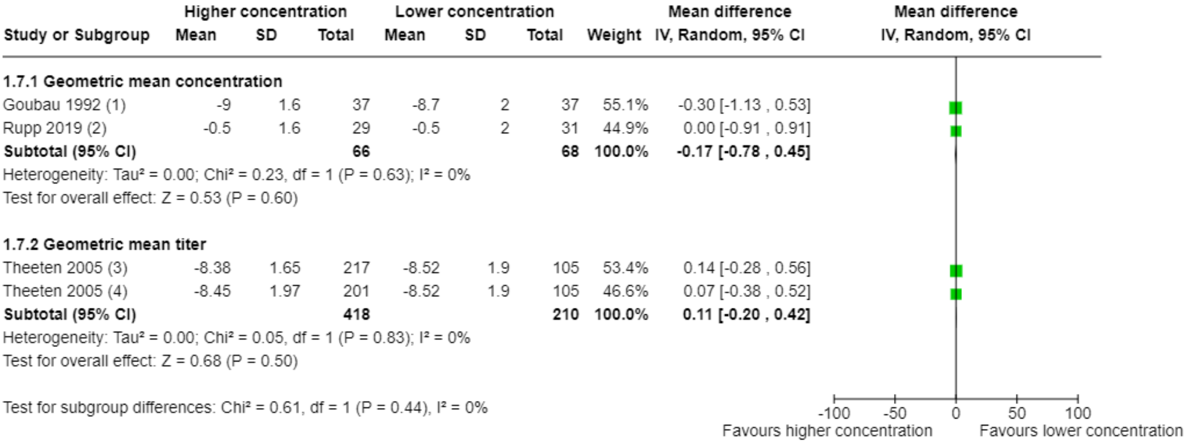

Footnotes
(1) Aluminium hydroxide 1000 mcg (1 dose) vs aluminium hydroxide 500 mcg (1 dose)
(2) Aluminium phosphate 250 mcg (1 dose) vs aluminium phosphate 125 mcg (1 dose)
(3) Aluminium hydroxide 500 mcg (1 dose) vs aluminium hydroxide 133 mcg (1 dose)
(4) Aluminium hydroxide 300 mcg (1 dose) vs aluminium hydroxide 133 mcg (1 dose)

For the serological response, we used the geometric mean concentration or titer (GMC or GMT) extracted from the first assay reported in the journal publication by the trialists. If a trial tested multiple antigens, we included data from the first antigen of the first assay as presented in the publication.

For the meta-analysis of serological response, we transformed the GMT in the following way: we calculated the natural Log for both the GMT (or GMC) and for the CI. If standard deviations (SDs) were not reported, we obtained them from the CI: we first calculated a t-value from the number of participants (n) analysed for that outcome (t-value = TINV(1-0.95; n-1), and then we calculated the SD as follows: (SQRT(n))*(LogCIhigh-LogCIlow) / t value). In the meta-analysis, we plotted the GMT (or GMC) multiplied by -1 and the SD calculated as described above. When imputing the SD due to lack of reporting of CI by the trialists, an average SD was calculated from the other trials that was supposed to be used in the meta-analysis and presented in sensitivity analyses.

Comparison 2 – Higher versus lower number of doses of aluminium adjuvant

All-cause mortality

Study or Subgroup	Higher no. of doses Events	Higher no. of doses Total	Lower no. of doses Events	Lower no. of doses Total	Weight	Risk ratio M-H, Random, 95% CI
**2.1.1 2 doses versus 1 dose**						
August 2017 (1)	0	92	0	90		Not estimable
August 2017 (2)	0	89	0	89		Not estimable
August 2017 (3)	0	91	0	93		Not estimable
**Subtotal (95% CI)**		**272**		**272**		**Not estimable**
Total events:	0		0			
Heterogeneity: Not applicable						
Test for overall effect: Not applicable						
**2.1.2 4 doses versus 3 doses**						
de Kleijn 2000 (4)	0	31	0	30		Not estimable
de Kleijn 2000 (5)	0	32	0	35		Not estimable
**Subtotal (95% CI)**		**63**		**65**		**Not estimable**
Total events:	0		0			
Heterogeneity: Not applicable						
Test for overall effect: Not applicable						
**Total (95% CI)**		**335**		**337**		**Not estimable**
Total events:	0		0			
Heterogeneity: Not applicable						
Test for overall effect: Not applicable						
Test for subgroup differences: Not applicable						

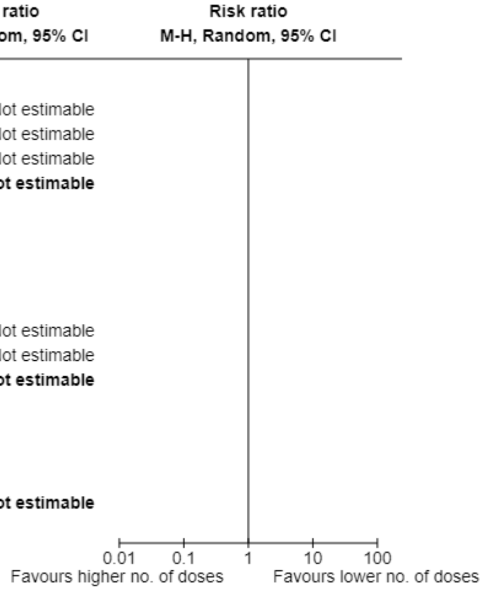

Footnotes
(1) Aluminium phosphate 200 mcg (2 doses) vs aluminium phosphate 200 mcg (1 dose)
(2) Aluminium phosphate 400 mcg (2 doses) vs aluminium phosphate 400 mcg (1 dose)
(3) Aluminium phosphate 800 mcg (2 doses) vs aluminium phosphate 800 mcg (1 dose)
(4) Aluminium hydroxide 860 mcg 4 (doses) vs aluminium hydroxide 860 mcg (3 doses)
(5) Aluminium phosphate 1340 mcg (4 doses) versus aluminium phosphate 1340 mcg (3 doses)

Serious adverse events

Study or Subgroup	Higher no. of doses Events	Higher no. of doses Total	Lower no. of doses Events	Lower no. of doses Total	Weight	Risk ratio M-H, Random, 95% CI
**2.2.1 2 doses versus 1 dose**						
August 2017 (1)	0	92	1	90	13.6%	0.33 [0.01 , 7.90]
August 2017 (2)	2	89	2	89	36.9%	1.00 [0.14 , 6.94]
August 2017 (3)	2	91	4	93	49.5%	0.51 [0.10 , 2.72]
**Subtotal (95% CI)**		**272**		**272**	**100.0%**	**0.62 [0.19 , 2.00]**
Total events:	4		7			
Heterogeneity: Tau² = 0.00; Chi² = 0.44, df = 2 (P = 0.80); I² = 0%						
Test for overall effect: Z = 0.81 (P = 0.42)						
**2.2.2 4 doses versus 3 doses**						
de Kleijn 2000 (4)	0	35	0	32		Not estimable
de Kleijn 2000 (5)	0	34	0	30		Not estimable
**Subtotal (95% CI)**		**69**		**62**		**Not estimable**
Total events:	0		0			
Heterogeneity: Not applicable						
Test for overall effect: Not applicable						
**Total (95% CI)**		**341**		**334**	**100.0%**	**0.62 [0.19 , 2.00]**
Total events:	4		7			
Heterogeneity: Tau² = 0.00; Chi² = 0.44, df = 2 (P = 0.80); I² = 0%						
Test for overall effect: Z = 0.81 (P = 0.42)						
Test for subgroup differences: Not applicable						

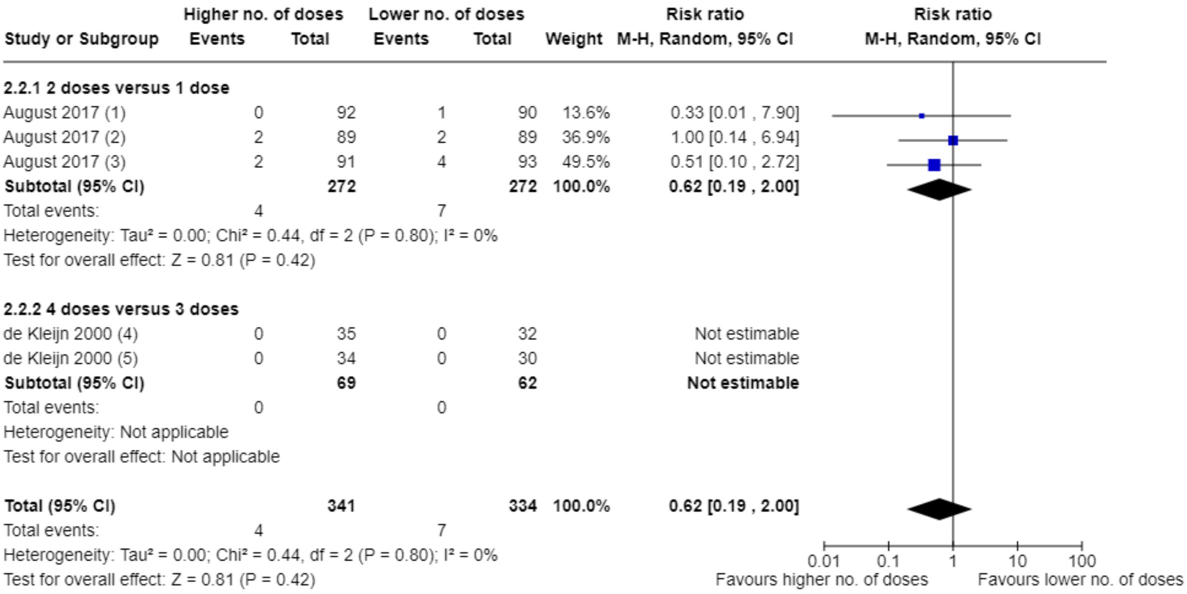

Footnotes

(1) Aluminium phosphate 200 mcg (2 doses) vs aluminium phosphate 200 mcg (1 dose)
(2) Aluminium phosphate 400 mcg (2 doses) vs aluminium phosphate 400 mcg (1 dose)
(3) Aluminium phosphate 800 mcg (2 doses) vs aluminium phosphate 800 mcg (1 dose)
(4) Aluminium phosphate 1340 mcg (4 doses) vs aluminium phosphate 1340 mcg (3 doses)
(5) Aluminium hydroxide 860 mcg 4 (doses) vs aluminium hydroxide 860 mcg (3 doses)

Individual serious adverse events

Comparison 2. Individual serious adverse events reported by the included trials

Type of serious adverse event	Trial	Trial Group	Events / analysed
**Chlamydial cervicitis**	August 2017	400 mcg aluminium phosphate (2 doses)	1/89
**Pneumonia**	August 2017	800 mcg aluminium phosphate (1 dose)	1/93
**Sepsis**	August 2017	800 mcg aluminium phosphate (1 dose)	1/93
**Borderline mucinous tumour of ovary**	August 2017	400 mcg aluminium phosphate (2 doses)	1/89
**Ovarian germ cell teratoma benign**	August 2017	800 mcg aluminium phosphate (1 dose)	1/93
**Squamous cell carcinoma of the cervix**	August 2017	800 mcg aluminium phosphate (2 doses)	1/91
**Brain injury**	August 2017	800 mcg aluminium phosphate (1 dose)	1/93
**Abortion spontaneous**	August 2017	400 mcg aluminium phosphate (1 dose)	1/89
	August 2017	200 mcg aluminium phosphate (1 dose)	1/90
**Ectopic pregnancy**	August 2017	400 mcg aluminium phosphate (1 dose)	1/89
**Anxiety**	August 2017	800 mcg aluminium phosphate (1 dose)	1/93
**Borderline personality disorder**	August 2017	800 mcg aluminium phosphate (1 dose)	1/93
**Major depression**	August 2017	800 mcg aluminium phosphate (1 dose)	1/93
**Suicidal ideation**	August 2017	800 mcg aluminium phosphate (1 dose)	1/93
**Suicide attempt**	August 2017	800 mcg aluminium phosphate (1 dose)	1/93
**Calculus ureteric**	August 2017	800 mcg aluminium phosphate (2 doses)	1/91
**Dysfunctional uterine bleeding**	August 2017	800 mcg aluminium phosphate (2 doses)	1/91
**Pneumonia aspiration**	August 2017	800 mcg aluminium phosphate (1 dose)	1/93

Adverse events

Study or Subgroup	Higher no. of doses Events	Higher no. of doses Total	Lower no. of doses Events	Lower no. of doses Total	Weight	Risk ratio M-H, Random, 95% CI
**2.3.1 2 doses versus 1 dose**						
August 2017 (1)	76	92	81	90	32.1%	0.92 [0.82 , 1.03]
August 2017 (2)	81	89	72	89	31.3%	1.13 [1.00 , 1.27]
August 2017 (3)	83	91	83	93	36.6%	1.02 [0.93 , 1.12]
**Subtotal (95% CI)**		**272**		**272**	**100.0%**	**1.02 [0.91 , 1.13]**
Total events:	240		236			
Heterogeneity: Tau² = 0.01; Chi² = 5.70, df = 2 (P = 0.06); I² = 65%						
Test for overall effect: Z = 0.32 (P = 0.75)						
**Total (95% CI)**		**272**		**272**	**100.0%**	**1.02 [0.91 , 1.13]**
Total events:	240		236			
Heterogeneity: Tau² = 0.01; Chi² = 5.70, df = 2 (P = 0.06); I² = 65%						
Test for overall effect: Z = 0.32 (P = 0.75)						
Test for subgroup differences: Not applicable						

Risk ratio M-H, Random, 95% CI

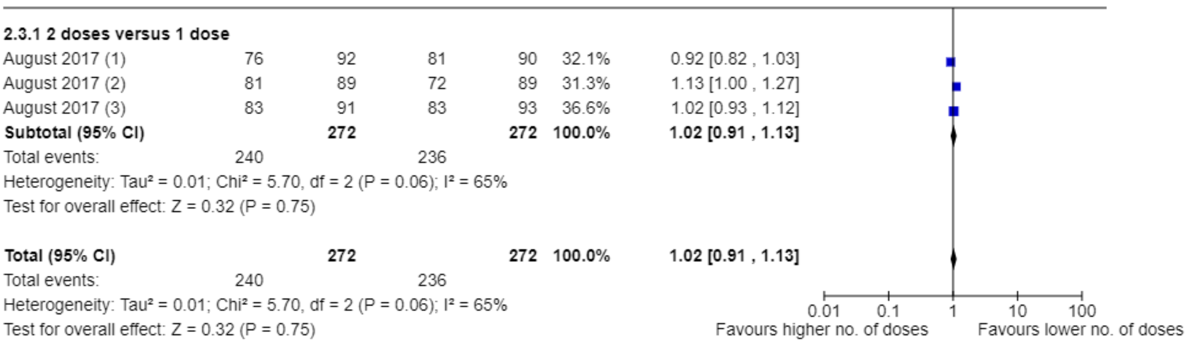

Footnotes

(1) Aluminium phosphate 200 mcg (2 doses) vs aluminium phosphate 200 mcg (1 dose)

(2) Aluminium phosphate 400 mcg (2 doses) vs aluminium phosphate 400 mcg (1 dose)

(3) Aluminium phosphate 800 mcg (2 doses) vs aluminium phosphate 800 mcg (1 dose)

Adverse events – Trial Sequential Analysis

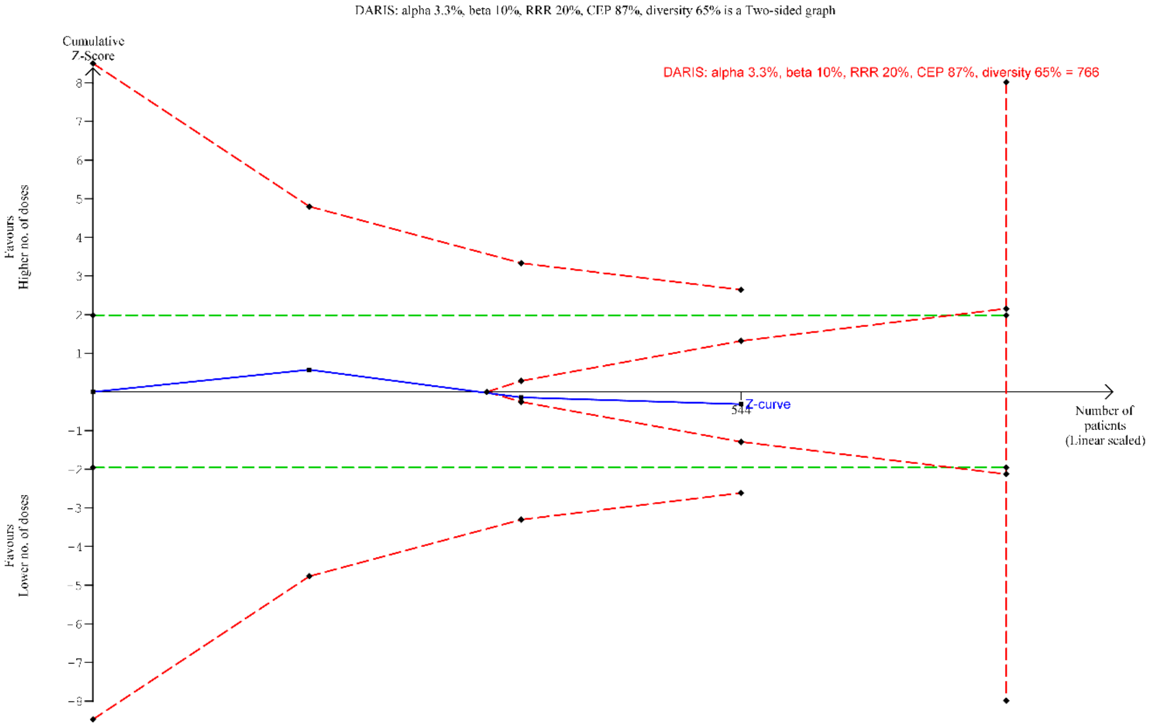

Individual adverse events

26 different types of adverse events not considered serious were reported by one trial.

Adverse event	Trial
Muscle pain	August 2017
Nausea	August 2017
Joint pain	August 2017
Chills	August 2017
Anaemia	August 2017
Abdominal pain	August 2017
Abdominal pain upper	August 2017
Influenzae-like illness	August 2017
Gastroenteritis	August 2017
Gastroenteritis viral	August 2017
Influenzae	August 2017
Pharyngitis	August 2017
Pharyngitis streptococal	August 2017
Tonsilitis	August 2017
Urinary tract infection	August 2017
Ligament strain	August 2017
Alanine aminotransferase increased	August 2017
Blood pressure diastolic increased	August 2017
Haemoglobin decreased	August 2017
Respiratory rate increased	August 2017
Arthralgia	August 2017
Back pain	August 2017
Myalgia	August 2017
Neck pain	August 2017
Dysmenorrhea	August 2017
Throat irritation	August 2017

Serological response

Study or Subgroup	Higher no. of doses Mean	SD	Total	Lower no. of doses Mean	SD	Total	Weight	Mean difference IV, Fixed, 95% CI
**2.6.1 Geometric mean titer**								
de Kleijn 2000 (1)	-3.84	1.79	32	-3.22	1.79	28	51.2%	-0.62 [-1.53 , 0.29]
de Kleijn 2000 (2)	-4.25	1.79	29	-3.24	1.79	28	48.8%	-1.01 [-1.94 , -0.08]
**Subtotal (95% CI)**			**61**			**56**	**100.0%**	**-0.81 [-1.46 , -0.16]**
Heterogeneity: Chi² = 0.35, df = 1 (P = 0.56); I² = 0%								
Test for overall effect: Z = 2.45 (P = 0.01)								
**Total (95% CI)**			**61**			**56**	**100.0%**	**-0.81 [-1.46 , -0.16]**
Heterogeneity: Chi² = 0.35, df = 1 (P = 0.56); I² = 0%								
Test for overall effect: Z = 2.45 (P = 0.01)								
Test for subgroup differences: Not applicable								

Mean difference IV, Fixed, 95% CI

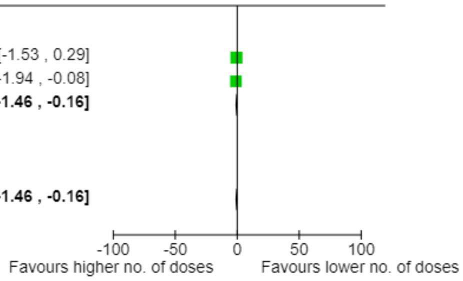

Footnotes

(1) Aluminium hydroxide 860 mcg 4 (doses) vs aluminium hydroxide 860 mcg (3 doses)

(2) Aluminium phosphate 1340 mcg (4 doses) vs aluminium phosphate 1340 mcg (3 doses)

Comparison 3 – Aluminium phosphate versus aluminium hydroxide

All-cause mortality

Study or Subgroup	Aluminium phosphate Events	Aluminium phosphate Total	Aluminium hydroxide Events	Aluminium hydroxide Total	Weight	Risk ratio M-H, Random, 95% CI
de Kleijn 2000 (1)	0	35	0	30		Not estimable
de Kleijn 2000 (2)	0	32	0	31		Not estimable
Leroux-Roels 2013 (3)	0	30	0	30		Not estimable
**Total (95% CI)**		**97**		**91**		**Not estimable**
Total events:	0		0			

Heterogeneity: Not applicable
Test for overall effect: Not applicable
Test for subgroup differences: Not applicable

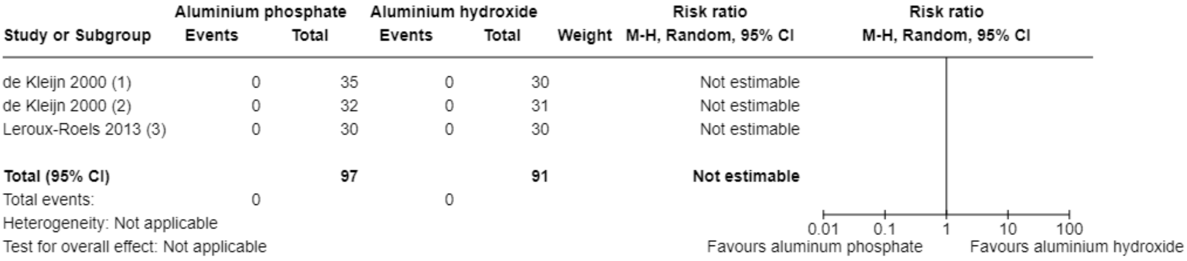

Footnotes
(1) Aluminium phosphate 1340 mcg (3 doses) vs aluminium hydroxide 860 mcg (3 doses)
(2) Aluminium phosphate 1340 mcg (4 doses) vs aluminium hydroxide 860 mcg (4 doses)
(3) Aluminium phosphate 500 mcg (1 dose) vs aluminium hydroxide 500 mcg (1 dose)

Serious adverse events

Study or Subgroup	Aluminium phosphate Events	Aluminium phosphate Total	Aluminium hydroxide Events	Aluminium hydroxide Total	Weight	Risk ratio M-H, Random, 95% CI
de Kleijn 2000 (1)	0	32	0	31		Not estimable
de Kleijn 2000 (2)	0	35	0	30		Not estimable
Leroux-Roels 2013 (3)	1	30	0	30	100.0%	3.00 [0.13 , 70.83]
**Total (95% CI)**		**97**		**91**	**100.0%**	**3.00 [0.13 , 70.83]**
Total events:	1		0			

Heterogeneity: Not applicable
Test for overall effect: Z = 0.68 (P = 0.50)
Test for subgroup differences: Not applicable

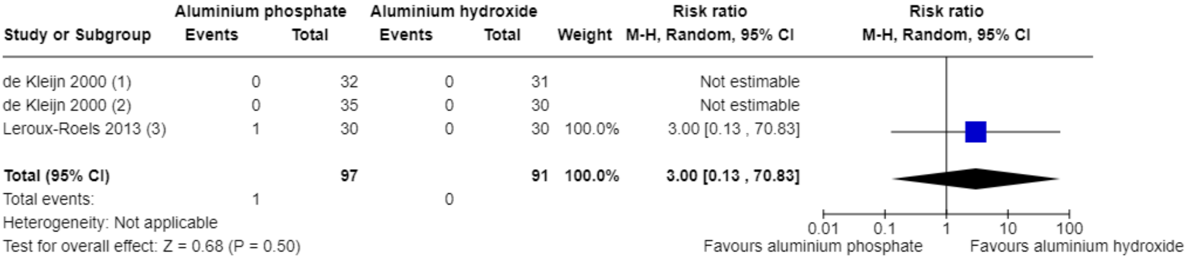

Footnotes
(1) Aluminium phosphate 1340 mcg (4 doses) vs aluminium hydroxide 860 mcg (4 doses)
(2) Aluminium phosphate 1340 mcg (3 doses) vs aluminium hydroxide 860 mcg (3 doses)
(3) Aluminium phosphate 500 mcg (1 dose) vs aluminium hydroxide 500 mcg (1 dose)

Adverse events

Study or Subgroup	aluminium phosphate Events	aluminium phosphate Total	aluminium hydroxide Events	aluminium hydroxide Total	Weight	Risk ratio M-H, Fixed, 95% CI
Leroux-Roels 2013 (1)	17	30	15	30	100.0%	1.13 [0.70 , 1.82]
**Total (95% CI)**		**30**		**30**	**100.0%**	**1.13 [0.70 , 1.82]**
Total events:	17		15			
Heterogeneity: Not applicable						
Test for overall effect: Z = 0.52 (P = 0.61)						
Test for subgroup differences: Not applicable						

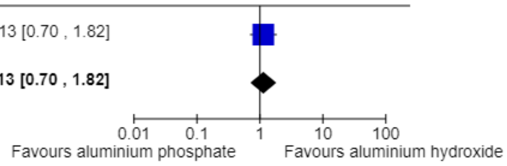

Footnotes
(1) Aluminium phosphate 500 mcg (1 dose) vs aluminium hydroxide 500 mcg (1 dose)

Adverse events – Trial Sequential Analysis

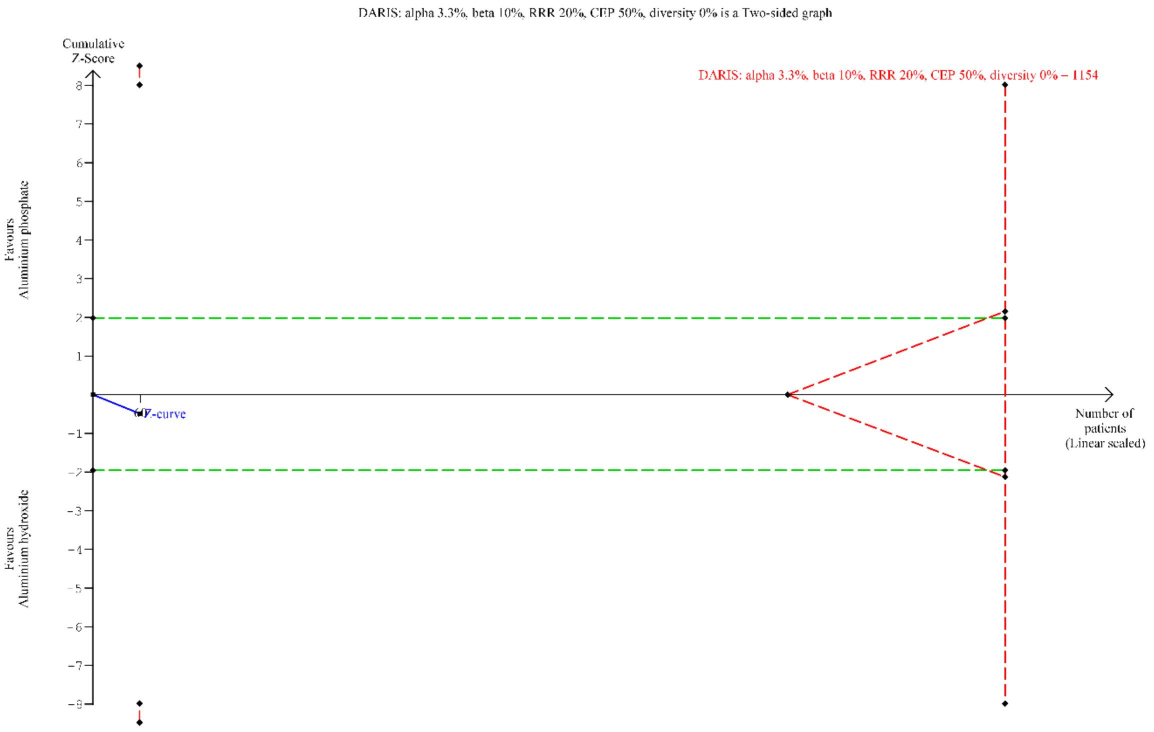

Individual adverse events

Three different types of adverse events not considered serious were reported by one trial.

Adverse event	Trial
Muscle stiffness	Leroux-Roels 2013
Soreness	Leroux-Roels 2013
Malaise	Leroux-Roels 2013

Serological response

Study or Subgroup	Aluminium phosphate Mean	SD	Total	Aluminium hydroxide Mean	SD	Total	Weight	Mean difference IV, Fixed, 95% CI
**3.6.1 Geometric mean titer**								
de Kleijn 2000 (1)	-4.25	1.79	29	-3.84	1.79	32	33.8%	-0.41 [-1.31 , 0.49]
de Kleijn 2000 (2)	-3.24	1.79	28	-3.22	1.79	28	31.1%	-0.02 [-0.96 , 0.92]
Leroux-Roels 2013 (3)	-7.49	2.02	30	-7.21	1.41	30	35.2%	-0.28 [-1.16 , 0.60]
**Subtotal (95% CI)**			**87**			**90**	**100.0%**	**-0.24 [-0.77 , 0.28]**
Heterogeneity: Chi² = 0.36, df = 2 (P = 0.84); I² = 0%								
Test for overall effect: Z = 0.91 (P = 0.36)								
**Total (95% CI)**			**87**			**90**	**100.0%**	**-0.24 [-0.77 , 0.28]**
Heterogeneity: Chi² = 0.36, df = 2 (P = 0.84); I² = 0%								
Test for overall effect: Z = 0.91 (P = 0.36)								
Test for subgroup differences: Not applicable								

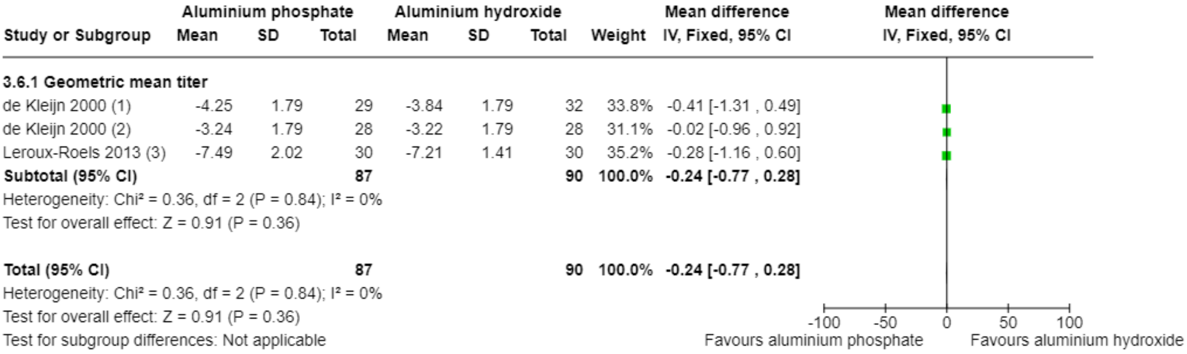

Footnotes

(1) Aluminium phosphate 1340 mcg (4 doses) vs aluminium hydroxide 860 mcg (4 doses)
(2) Aluminium phosphate 1340 mcg (3 doses) vs aluminium hydroxide 860 mcg (3 doses)
(3) Aluminium phosphate 500 mcg (1 dose) vs aluminium hydroxide 500 mcg (1 dose)

DIFFERENCES BETWEEN PROTOCOL AND REVIEW

1. We did not specifically search for observational studies (quasi-randomised studies; cohort studies; and patient series), and we abandoned our plan to provide a narrative account of such data.

2. We planned to include data from EMA, the FDA, other regulatory authorities, and pharmaceutical companies' trial registries. However, due to difficulties encountered during our process and work, we did not receive any data. First, the regulatory authorities and pharmaceutical companies' trial registries did not allow sharing data on products that did not achieve licenses for the markets. Second, our requests for published or unpublished clinical study reports assessing any aluminium containing vaccines (licensed for use in Europe and US or not yet/any more licensed) was incompatible with the requirements of the EMA and the FDA to indicate the name(s) of any licensed vaccine(s) for which we were seeking clinical study reports and, for each named product, to specify each clinical study for which we were seeking the clinical study report.

3. We planned to include particle size (nanosize or microsize as described by trialists or manufacturers) as a covariate in meta-regression to assess whether particle size influences the effect of aluminium adjuvant administration on outcomes. However, due to lack of relevant data, we could not perform this analysis.

REFERENCES

Abbasi J. 2022. COVID-19 Vaccine Focused on T-Cell Response Promising in Early Trial. JAMA. 327(2):115.

Actrn. 2020. A Phase 1/2 randomized, placebo-controlled, multi-centre study to evaluate the safety and immunogenicity of a novel Receptor Binding Domain (RBD) COVID-19 Vaccine in Healthy Adults. http://wwwwhoint/trialsearch/Trial2aspx?TrialID=ACTRN12620001308987.

Ajjan N, Triau R. 1975. Anti-measles vaccine and tuberculin-type allergy. Pediatrie. 30(1):29-44.

Amato Neto V, de Oliveira e Silva YK, Finger H, Bianchi A, do Amaral JP, Cardozo BS, de Lima Filho MT, Netto NV. 1973. Analysis of the adverse effects occuring after the administration of BCG by oral route in 5,579 healthy children. Revista paulista de medicina. 82(3):135-138.

Anonymous. 1969. BCG vaccination against mycobacterium ulcerans infection (Buruli ulcer). First results of a trial in Uganda. Lancet (London, England). 1(7586):111-115.

Anonymous. 1973. Comparison of several BCG vaccines and methods of administration. South African medical journal. Suid-Afrikaanse Tydskrif Vir Geneeskunde. 47(46):2207-2218.

Anonymous. 2001a. Options for managing influenza are increasing. Drugs and therapy perspectives. 17(11):12-15.

Anonymous. 2001b. Phase III hepatitis C program begins. AIDS reader. 11(2):85.

Anonymous. 2001c. Phase III trial in hepatitis B prevention. AIDS reader. 11(2):85.

Anonymous. 2009. Glatiramer: a second look. With longer follow-up: still no proven benefit in multiple sclerosis. Prescrire international. 18(104):252.

Anonymous. 2017a. Corrigendum to: polyethylenimine-based micro/nanoparticles as vaccine adjuvants [Corrigendum] (Int J Nano, (2017), 12, (5443-5460)). International journal of nanomedicine. 12(pp 7239).

Anonymous. 2017b. Shingrix - An adjuvanted, ecombinant herpes zoster vaccine. Medical letter on drugs and therapeutics. 59(1535):195-197.

Arif S, Gomez-Tourino I, Kamra Y, Pujol-Autonell I, Hanton E, Tree T, Melandri D, Hull C, Wherrett DK, Beam C et al. 2020. GAD-alum immunotherapy in type 1 diabetes expands bifunctional Th1/Th2 autoreactive CD4 T cells. Diabetologia. 63(6):1186-1198.

Aslam S, Ahmad S, Noor F, Ashfaq Usman A, Shahid F, Rehman A, Tahir ul Qamar M, Alatawi Eid A, Alshabrmi Fahad M, Allemailem Khaled S. 2021. Designing a Multi-Epitope Vaccine against Chlamydia trachomatis by Employing Integrated Core Proteomics, Immuno-Informatics and In Silico Approaches. Biology-basel. 10(10):997.

Authier F, Gherardi R. 2006. Safety and immunogenicity of H5N1 vaccine. Lancet. 368(9554):2209-2210.

Azurin JC, Cruz A, Pesigan TP, Alvero M, Camena T, Suplido R, Ledesma L, Gomez CZ. 1967. A controlled field trial of the effectiveness of cholera and cholera El Tor vaccines in the Philippines. Bulletin of the World Health Organization. 37(5):703-727.

Baldo V, Menegon T, Buoro S, Scalici C, Vesco A, Perale S, et al. 1999. Vaccination against influenza in the elderly. Experience with adjuvant vaccines]. [Italian. Annali di Igiene: Medicina Preventiva e di Comunita`. 11(5):369-374.

Bankhead C. 2007. Preliminary breast cancer vaccine study shows promise. Journal of the National Cancer Institute. 99(11):836.

Barouch DH, Tomaka FL, Wegmann F, Stieh DJ, Alter G, Robb ML, Michael NL, Peter L, Nkolola JP, Borducchi EN et al. 2018. Evaluation of a mosaic HIV-1 vaccine in a multicentre, randomised, double-blind, placebo-controlled, phase 1/2a clinical trial (APPROACH) and in rhesus monkeys (NHP 13-19). Lancet. 392(10143):232-243.

Baxendale W, Lutticken D. 1982. THE RESULTS OF FIELD TRIALS WITH AN INACTIVATED GUMBORO VACCINE. Moreau, y And w Hennessen (ed) Developments in biological standardization, vol 51 Immunization of adult birds with inactivated oil adjuvant vaccines; symposium, lyon, france, june 25-27, 1981 IX+332P S Karger: basel, switzerland; new york, ny.P211-220.

Bechelli LM, Garbajosa PG, Gyi MM, Uemura K, Engler V, Dominguez VM, Sundaresan T, Quagliato R, Matejka M. 1971. BCG vaccination of children against leprosy. Preliminary findings of the WHO-controlled trial in Burma up to January 1970. International journal of leprosy and other mycobacterial diseases. 39(2):609.
Brown AE, Steinherz PG, Miller DR, Armstrong D, Kellick G, Gross PA, et al. 1982. Immunization against influenza in children with cancer: results of a three-dose trial. Journal of infectious diseases. 145(1):126.
Brown J, Baisley K, Kavishe B, Changalucha J, Andreasen A, Mayaud P, Gumodoka B, Kapiga S, Hayes R, Watson-Jones D. 2014. Impact of malaria and helminth infections on immunogenicity of the human papillomavirus-16/18 AS04-adjuvanted vaccine in Tanzania. Vaccine. 32(5):611-617.
Brugh M, Stone HD, Lupton HW. 1983. Comparison of inactivated Newcastle disease viral vaccines containing different emulsion adjuvants. American journal of veterinary research. 44(1):72-75.
Bulbul A, Yilmaz IC, Yilmaz N, Turay N, Ipekoglu EM, Guvencli N, Yildirim M, Canavar T, Evcili I, Bildik T et al. 2021. Manufacturing of a SARS-CoV-2 virus-like particle vaccine adjuvanted with K3 CpG oligodeoxynucleotide and alhydrogel for phase 1/2 clinical trials. European journal of immunology. 51:370-370.
Burny W, Callegaro A, Bechtold V, Clement F, Delhaye S, Fissette L, Leroux-Roels G, Marchant A, van den Berg RA, Garcon N et al. 2017. Different adjuvants induce common innate pathways that are associated with enhanced adaptive responses against a model antigen in humans. Frontiers in immunology. 8(AUG) (no pagination):14-30.
Bystryn JC, Oratz R, Harris MN, Roses DF, Golomb FM, Speyer JL. 1988. Immunogenicity of a polyvalent melanoma antigen vaccine in humans. Cancer. 61(6):1065-1070.
Chichester JA, Green BJ, Jones RM, Shoji Y, Miura K, Long CA, Lee CK, Ockenhouse CF, Morin MJ, Streatfield SJ et al. 2018. Safety and immunogenicity of a plant-produced Pfs25 virus-like particle as a transmission blocking vaccine against malaria: a Phase 1 dose-escalation study in healthy adults. Vaccine. 36(39):5865-5871.
Collier LH, Polakoff S, Mortimer J. 1979. Reactions and antibody responses to reinforcing doses of adsorbed and plain tetanus vaccines. Lancet (London, England). 1(8131):1364-1368.
Comstock GW, Edwards PQ. 1972. An American view of BCG vaccination, illustrated by results of a controlled trial in Puerto Rico. Scandinavian journal of respiratory diseases. 53(4):207-217.
Coulaud IP, Gougeon ML, Gomard E, Descamps D, Lebon P, Aboulker JP, Bizzini B, Zagury D. 1997. A placebo-controlled clinical phase I trial with combined anti-HIV-1 and anti-interferon-alpha immunization. AIDS (London, England). 11(7):937-938.
D'Arcy Hart P, Pollock TM, Sutherland I. 1957. Assessment of the first results of the Medical Research Council's trial of tuberculosis vaccines in adolescents in Great Britain. Bibliotheca tuberculosea. 13(12):171-189.
De Serres G. 2014. Selection bias in the comparison between adjuvanted and unadjuvanted influenza vaccine effectiveness. Vaccine. 32(49):6596.
Di Mario S, Basevi V, Borsari S, Balduzzi S, Magrini N. 2012. Overall efficacy of HPV-16/18 AS04-adjuvanted vaccine. Lancet oncology. 13(2):E50-E50.
Diaz C, Koren M, Lin L, Martinez LJ, Eckels KH, Campos M, Jarman RG, De La Barrera R, Lepine E, Febo I et al. 2020. Safety and Immunogenicity of Different Formulations of a Tetravalent Dengue Purified Inactivated Vaccine in Healthy Adults from Puerto Rico: final Results after 3 Years of Follow-Up from a Randomized, Placebo-Controlled Phase I Study. American journal of tropical medicine and hygiene. 102(5):951-954.
Diemert DJ, Zumer M, Campbell D, Grahek S, Li G, Peng J, Elena Bottazzi M, Hotez P, Bethony J. 2022. Safety and immunogenicity of the Na-APR-1 hookworm vaccine in infection-naive adults. Vaccine. 40(42):6084-6092.
Dingermann T. 2022. Adjuvanted and high-dose: comparison of influenza vaccines in the elderly. Pharmazeutische Zeitung. 167(23):36-39.

Dowlati Y, Ehsasi S, Shidani B, Bahar K. 1996. Stepwise safety trial of a killed Leishmania vaccine in Iran. Clinics in dermatology. 14(5):497-502.
Drachenberg KJ, Mitsch A, Pfeiffer P. 1999. Short-Term Therapeutic Allergy Vaccination: a Multi-Centre, Randomised Double-Blind Placebo-Controlled Study With L-Tyrosine-Adsorbed Pollen Allergoids. Immunology. 98(suppl. 1):140.
Dunkle LM, Kotloff KL, Gay CL, Anez G, Adelglass JM, Hernandez ABQ, Harper WL, Duncanson DM, McArthur MA, Florescu DF et al. 2022. Efficacy and Safety of NVX-CoV2373 in Adults in the United States and Mexico. New England journal of medicine.
Egerton JR, Thompson JJ, Merritt GC. 1979. Comparison of oil adjuvant and alum precipitated Bacteroides nodosus vaccines in treatment of foot-rot. Veterinary record. 104(5):98-100.
Evidence-based review of clinical studies on trauma. 2009. Journal of endodontics. 35(8):1160-1162.
Fernandez-Cruz E, Munoz-Fernandez MA, Navarro J, Clotet B, Gonzalez-Lahoz J, Moreno S, Pena JM, Perez Molina J, Podzamczer D, Lopez P. 2001. Randomized Control Phase Ii Trial Evaluating Hiv-1 Immunogen Administered to Patients With Hiv-1 Chronic Infection Having 300 to 700 Cells/Ml Cd4 Cell Counts: data From a Recently Unblinded Study. Journal of human virology. 4(3):156.
Fox EN, Pachman LM, Wittner MK, Dorfman A. 1970. Primary immunization of infants and children with group A streptococcal M protein. Zentralblatt fur Bakteriologie, Parasitenkunde, Infektionskrankheiten und Hygiene 1 Abt Medizinisch-hygienische Bakteriologie, Virusforschung und Parasitologie Originale. 214(3):352-363.
Frasch CE, Zahradnik JM. 1986. Human immune response to an aluminum hydroxide absorbed Neisseria meningitidis serotype 2b protein vaccine. Antonie van leeuwenhoek, international journal of general and molecular microbiology. 52(3):229-231.
Fries LF, Smith GE, Glenn GM. 2013. A recombinant viruslike particle influenza A (H7N9) vaccine. New England journal of medicine. 369(26):2564-2566.
Gao Q, Li CG, Liu SZ. 2008. Development of a method for determination of hemagglutinin content in pandemic influenza vaccine containing aluminium adjuvant. Chinese journal of biologicals. 21(1):60-61+69.
Gardner Jason P, Slovin Susan F, Schuelke N, Morrissey Donna M, Donovan Gerald P, Surowitz Kenneth G, Olson William C, Goeckeler William F, Scher Howard I, Israel Robert J. 2004. Phase I trial of an adjuvanted recombinant soluble prostate-specific membrane antigen (rsPSMA) vaccine. Proceedings of the american association for cancer research annual meeting. 45:284.
GlaxoSmithKline Vaccine HPV-007 Study Group. 2009. Sustained efficacy a,d immunogenicity of the human papillomavirus (HPV)-16/18 ASO4-adjuvanted vaccine: analysis of a randomised placebo-controlled trial up to 6.4 years. Lancet. 374:1975-1985.
Gotch FM, Imami N. 2001. Outcomes of a trial of HIV-1 immunogen in patients with HIV infection. JAMA. 285(17):2191-2192; author reply 2193.
Herrington DA, Clyde DF, Losonsky G. 1987. Safety and immunogenicity in man of a synthetic peptide malaria vaccine against Plasmodium falciparum sporozoites. Nature. 328(6127):257-259.
Houser KV, Gaudinski MR, Happe M, Narpala S, Verardi R, Sarfo EK, Corrigan AR, Wu R, Rothwell RS, Novik L et al. 2022. Safety and immunogenicity of an HIV-1 prefusion-stabilized envelope trimer (Trimer 4571) vaccine in healthy adults: a first-in-human open-label, randomized, dose-escalation, phase 1 clinical trial. EClinicalMedicine. 48.
Joob B, Wiwanitkit V. 2018. Comparison between adjuvanted and nonadjuvanted influenza vaccination among patients undergoing hemodialysis: a cost-effectiveness analysis from Thailand. Saudi journal of kidney diseases and transplantation. 29(2):474-475.
Kanai K, Takehiro A, Noto H. 1985. Prevention of perinatal transmission of hepatitis B virus (HBV) to children of e antigen-positive HBV carrier mothers by hepatitis B immune globulin and HBV vaccine. Journal of infectious diseases. 151(2):287-290.
Keam SJ, Harper DM. 2008. Human Papillomavirus Types 16 and 18 Vaccine (Recombinant, As04 Adjuvanted, Adsorbed). Biodrugs. 22(3):205-208.

Keefer MC, Belshe R, Graham B, McElrath J, Clements ML, Sposto R, Fast P. 1994. Safety profile of HIV vaccination: first 1000 volunteers of AIDS vaccine evaluation group. NIAID AIDS Vaccine Clinical Trials Network. AIDS research and human retroviruses. 10 Suppl 2:S139-140.

Knuf M, Habermehl P, Faber J, Bock HL, Saenger R, Bogaerts H, Clemens R, Schuind A, Du Prel JB, Schmitt HJ. 2006. Assessment of Nine Candidate Dtp-Vaccines With Reduced Amount of Antigen and/or Without Adjuvant as a Fourth (Booster-) Dose in the Second Year of Life. Vaccine. 24(27-28):5627-5636.

Kong NCT, Beran J, Kee SA, Miguel JL, Sanchez C, Bayas JM, Vilella A, de J, Jr., Arrazola P, Calbo-Torrecillas F et al. 2005. Immunogenicity and safety of an adjuvanted hepatitis B vaccine in pre-haemodialysis and haemodialysis patients. Nephrology (Carlton, Vic). 10(Suppl):A187.

Kooijman S, Brummelman J, Van Els C, Marino F, Heck AJR, Van Riet E, Metz B, Kersten GFA, Pennings JLA, Meiring HD. 2018. Vaccine antigens modulate the innate response of monocytes to Al(OH)<inf>3</inf>. PloS one. 13(5).

Kreijtz J, Goeijenbier M, Moesker FM, van den Dries L, Goeijenbier S, De Gruyter HLM, Lehmann MH, de Mutsert GD, van de Vijver D, Volz A et al. 2014. Safety and immunogenicity of a modified-vaccinia-virus-Ankara-based influenza A H5N1 vaccine: a randomised, double-blind phase 1/2a clinical trial. The Lancet Infectious diseases. 14(12):1196-1207.

Kulkarni PS, Manjunath K, Agarkhedkar S, Group of SIIIIVS. 2012. Safety and immunogenicity of an adjuvanted whole virion, inactivated A (H1N1) 2009 influenza vaccine in young and elderly adults, and children. Vaccine. 31(1):20-22.

Kuwert EK, Menzel H, Marcus I, Majer M. 1978. Antigenicity of low concentrated HDCS vaccine with and without adjuvant as compared to the standard fluid formulation. Developments in biological standardization. 40(pp 29-34).

Langley J, Frenette L, Ferguson L, Riff D, Folkerth S, Sheldon E, Segall N, Risi G, Middleton R, Johnson C et al. 2008. Safety and Cross-Reactive Immunogenicity of Two H5N1 A/Indonesia/5/2005 (Clade 2.1) AS-Adjuvanted Prepandemic Candidate Influenza Vaccines: a Phase I/II Clinical Trial. International journal of infectious diseases. 12(Suppl. 1):E146-E147.

Lell B, Agnandjil S, Von Glasenapp I, Oyakhiromen S, Haertle S, Kremsner Peter G, Ramboer I, Lievens M, Ballou R, Vekemans J et al. 2007. A Randomized, Observer-Blind Trial to Compare Safety and Immunogenicity of Two Adjuvanted Rts,S Anti-Malaria Vaccine Candidates in Gabonese Children. American journal of tropical medicine and hygiene. 77(5, Suppl. S):3.

Leroux-Roels G, Maes C, Willekens J, De Boever F, De Rooij R, Martell L, Bedell L, Wittke F, Slobod K, Dull P. 2016. A randomized, observer-blind phase ib study to identify formulations and vaccine schedules of a trivalent group B streptococcus vaccine for use in non-pregnant and pregnant women. Vaccine. 34(15):1786-1791.

Leroux-Roels G, Marchant A, Levy J, Van Damme P, Schwarz Tino F, Horsmans Y, Jilg W, Kremsner Peter G, Haelterman E, Clement F et al. 2016. Impact of adjuvants on CD4(+) T cell and B cell responses to a protein antigen vaccine: results from a phase II, randomized, multicenter trial. Clinical immunology (orlando, fla). 169:16-27.

Leroux-Roels G, Van Damme P, Haazen W, Shakib S, Caubet M, Aris E, Devaster J-M, Peeters M. 2016. Phase I, randomized, observer-blind, placebo-controlled studies to evaluate the safety, reactogenicity and immunogenicity of an investigational non-typeable haemophilus influenzae (nthi) protein vaccine in adults. Vaccine. 34(27):3156-3163.

Leroux-Roels G, Van Damme P, Haazen W, Shakib S, Caubet M, Aris E, Devaster J-M, Peeters M. 2017. Phase I, randomized, observer-blind, placebo-controlled studies to evaluate the safety, reactogenicity and immunogenicity of an investigational non-typeable Haemophilus influenzae (NTHi) protein vaccine in adults (vol 34, pg 3156, 2016). Vaccine. 35(42):5729-5730.

Loebermann M, Anders G, Brestrich G, Fritzsche C, Klammt S, Borso D, Frimmel S, Riebold D, Reisinger EC. 2011. Safety and immunogenicity of a trivalent single dose seasonal influenza vaccine containing pandemic A(H1N1) antigen in younger and elderly subjects: a phase III open-label single-arm study. Vaccine. 29(6):1228-1234.

Ma H, Lim Tien H, Leerapun A, Weltman M, Jia J, Lim Y-S, Tangkijvanich P, Sukeepaisarnjaroen W, Ji Y, Le Bert N et al. 2021. Therapeutic vaccine BRII-179 restores HBV-specific immune responses in patients with chronic HBV in a phase Ib/IIa study. JHEP reports. 3(6):100361.
Ma KSK, Lee CC, Liu KJ, Wang LT. 2021. Development of COVID-19 vaccines through augmenting antiviral immune responses: a systematic review and meta-analysis. European journal of immunology. Conference: 6th European Congress of Immunology. Virtual. 51(SUPPL 1):395.
Ma R, Taphoorn MJB, Plaha P. 2021. Advances in the management of glioblastoma. Journal of neurology, neurosurgery and psychiatry. 92(10):1103-1111.
Martins KAO, Cooper CL, Stronsky SM, Norris SLW, Kwilas SA, Steffens JT, Benko JG, van Tongeren SA, Bavari S. 2016. Adjuvant-enhanced CD4 T Cell Responses are Critical to Durable Vaccine Immunity. EBioMedicine. 3(pp 67-78).
McFarland G, La Joie E, Thomas P, Lyons-Weiler J. 2021. Corrigendum to "Acute exposure and chronic retention of aluminum in three vaccine schedules and effects of genetic and environmental variation". [J. Trace Elem. Med. Biol. 58 (2020) 126444] (Journal of Trace Elements in Medicine and Biology (2020) 58, (S0. Journal of trace elements in medicine and biology. 65(no pagination).
Meier S, Bel M, L'Huillier A, Crisinel PA, Combescure C, Kaiser L, Grillet S, Posfay-Barbe K, Siegrist CA. 2011. Antibody responses to natural influenza A/H1N1/09 disease or following immunization with adjuvanted vaccines, in immunocompetent and immunocompromised children. Vaccine. 29(19):3548-3557.
Meiklejohn G. 1960. Adjuvant polyvalent influenza - adrenovirus vaccine [abstract]. Journal of laboratory and clinical medicine. 56:927.
Moser C, Strezova A, Lal H, Enweonye I, Campora L, Beukelaers P, Segall N, Heineman TC, Schuind AE, Oostvogels L. 2020. The adjuvanted recombinant zoster vaccine co-administered with a tetanus, diphtheria and pertussis vaccine in adults aged >= 50 years: a randomized trial. Internist. 61(SUPPL 1):S33-S33.
Muderspach Laila I, Roman Lynda D, Facio G, Marty V, Bade E, Burnett Alexander F, Brader Kevin R, Morrow CP, Felix Juan C, Weber Jeffrey S. 1999. Phase I trial of a HPV-16 E7 peptide vaccine in women with high-grade cervical and vulvar dysplasia. Gynecologic oncology. 72(3):447.
Mustafa A. 2021. Adjuvants and Antigen-Delivery Systems for Subunit Vaccines against Tuberculosis. Vaccines. 9(9).
Nagaputra JC, Rollier CS, Sadarangani M, Hoe JC, Mehta OH, Norheim G, Saleem M, Chan H, Derrick JP, Feavers I et al. 2014. Neisseria meningitidis native outer membrane vesicles containing different lipopolysaccharide glycoforms as adjuvants for meningococcal and nonmeningococcal antigens. Clinical and vaccine immunology. 21(2):234-242.
Nardin EH, Calvo-Calle JM, Oliveira GA, Nussenzweig RS, Schneider M, Tiercy JM, et al. 2001. A totally synthetic polyoxime malaria vaccine containing Plasmodium falciparium B cell and universal T cell epitopes elicits immune responses in volunteers of diverse HLA types. Journal of immunology (Baltimore, Md : 1950). 166(1):481-489.
Nct. 1999. A Multicenter, Randomized, Placebo-Controlled, Double-Blind Trial to Evaluate the Safety and Immunogenicity of an HIV-1 Pseudovirion Vaccine. https://clinicaltrialsgov/show/NCT00000835.
Nct. 2005a. Human Papilloma Virus Vaccine Immunogenicity and Safety Trial in Young Adult Women With GSK Bio's Novel HPV Vaccine. https://clinicaltrialsgov/show/NCT00231413.
Nct. 2005b. Trial of rPA-102 Vaccine in Healthy Adult Volunteers. https://clinicaltrialsgov/show/NCT00100724.
Nct. 2006a. Human Papillomavirus Vaccine Immunogenicity and Safety Trial in Young Adult Women With GSK Biologicals Novel HPV Vaccine. https://clinicaltrialsgov/show/NCT00359619.
Nct. 2006b. Randomized, Double Blinded, Placebo Control Clinical Trial of Pandemic Influenza Inactive Vaccine on Healthy Subjects. https://clinicaltrialsgov/show/NCT00356798.
Nct. 2007a. A Phase 2 Study of Immunogenicity, Safety and Tolerability of CSL412 in Elderly Participants. https://clinicaltrialsgov/show/NCT00479648.

Nct. 2007b. Safety and Efficacy of SmithKline Beecham (GlaxoSmithKline [GSK]) Biologicals' Candidate Adjuvanted Vaccines (287615). https://clinicaltrialsgov/show/NCT00508833.
Nct. 2008. Study of Different Formulations of an Intramuscular A/H5N1 Inactivated, Split Virion Influenza Adjuvanted Vaccine. https://clinicaltrialsgov/show/NCT00664417.
Nct. 2009a. Safety, Tolerability, and Immunogenicity of Different Combinations of Trivalent Influenza Vaccine Varying Influenza Antigen Dose, Adjuvant Dose, and Route of Administration in Healthy Elderly Individuals Ages 65 Years and Older. https://clinicaltrialsgov/show/NCT00848848.
Nct. 2009b. A Study of Different Formulations of an Adjuvanted A/H1N1 Pandemic Vaccine in Healthy Adults and the Elderly. https://clinicaltrialsgov/show/NCT00952276.
Nct. 2009c. A Study of Inactivated Swine-Origin A/H1N1 Influenza Vaccines in Healthy European Subjects Aged 3 to 17 Years. https://clinicaltrialsgov/show/NCT00956202.
Nct. 2009d. A Study of Swine-origin A/H1N1 Influenza Vaccines in Healthy European Adults and the Elderly. https://clinicaltrialsgov/show/NCT00954798.
Nct. 2012a. A/H5N1 Virus-Like Particle Antigen Dose Ranging Study With Adjuvant 1. https://clinicaltrialsgov/show/NCT01594320.
Nct. 2012b. A/H5N1 Virus-Like Particle Antigen Dose Ranging Study With Adjuvant 2. https://clinicaltrialsgov/show/NCT01596725.
Nct. 2014. Study to Evaluate the Persistence of the Cellular and Humoral Immune Response Following Vaccinations With GlaxoSmithKline (GSK) Biologicals' Candidate Vaccines Containing HBsAg and Different Adjuvants in Healthy Adult Volunteers. https://clinicaltrialsgov/show/NCT02153320.
Nct. 2017. A Phase Ⅰ Study of the Recombinant Mycobacterium Tuberculosis Vaccine Freeze-dried. https://clinicaltrialsgov/show/NCT03026972.
Nct. 2020. Study of Recombinant Protein Vaccine Formulations Against COVID-19 in Healthy Adults 18 Years of Age and Older. https://clinicaltrialsgov/show/NCT04537208.
Nielsen PI, Kusk MV, Thulstrup N, Simonsen J. 2020. Immunogenicity and safety of an adjuvanted inactivated polio vaccine, IPV-Al, in primary and booster trials in Asia, Central America and Europe. International journal of infectious diseases. 101(Suppl. 1):471-472.
Pactr. 2022. Phase 1 Dose Escalating, Double-Blind, Randomised Comparator Controlled Trial of the Safety and Immunogenicity of Different Adjuvant Formulations of R0.6C and ProC6C transmission blocking vaccines candidates against Plasmodium falciparum in Adults in Burk. https://trialsearchwhoint/Trial2aspx?TrialID=PACTR202201848463189.
Pereira Junior E. 2000. Uso do bioterapico preparado a partir do precipitado da vacina triplice na prevencao dos efeitos adversos locais produzidos pela adjuvante hidroxido de aluminio existente no precipitado da vacina^ipt. 16(2):27-42.
Raponi A, Brewer JM, Garside P, Laera D. 2022. Corrigendum to "Nanoalum adjuvanted vaccines: small details make a big difference" [Semin. Immunol. 56 (2021) 101544] (Seminars in Immunology (2021) 56, (S1044532321000750), (10.1016/j.smim.2021.101544)). Seminars in immunology. 59(no pagination).
Ratnapriya S, Perez-Greene E, Schifanella L, Herschhorn A. 2021. Adjuvant-mediated enhancement of the immune response to HIV vaccines. FEBS journal.
Riedmann EM. 2012. Therapeutic vaccine for treatment of genital herpes enters phase 2. Human vaccines & immunotherapeutics. 8(12):1744.
Rosenbaum MJ, Poudstone JW, Edwards EA. 1976. Acceptability and antigenicity of adjuvant soluble sub-unit adenovirus vaccines in navy recruits. Military medicine. 141(6):383-388.
Roy P, Venugopalan AT. 1998. Potentiation of immune response of live lentogenic Newcastle disease vaccine using adjuvant. Tropical animal health and production. 30(1):37-39.
Rutter DA, Ashworth LAE, Day A, Funnell S, Lovell F, Robinson A. 1988. Trial of a new accellular pertussis vaccine in healthy adult volunteers. Vaccine. 6(1):29-32.
Sacks HS. 2017. In adults >= 70 years of age, an adjuvanted herpes zoster subunit vaccine reduced herpes zoster at a mean 3.7 years. Annals of internal medicine. 166(2):JC5.

Sarateanu DE, Ehrengut W. 1981a. Influenza vaccination, efficacy of content of antigen and an adjuvant with regard to humoral antibody production after influenza vaccination, double blind trial comparing several vaccines. Fortschritte der Medizin. 99(34):1348-1352.
Sarateanu DE, Ehrengut W. 1981b. Influenza vaccination. The importance of antigen amount and adjuvant with reference to the humoral antibody formation after influenza vaccination. A comparative double-blind study with various vaccines. Fortschritte der Medizin. 99(34):1348-1352.
Schmader KE, Levin MJ, Chen M, Matthews S, Riley M, Woo W, Herve C, Grupping K, Schuind A, Oostvogels L et al. 2019. Impact of reactogenicity on quality of life and physical functioning in adults >=50 years receiving both doses of the adjuvanted recombinant zoster vaccine. Open forum infectious diseases. Conference: Infectious Diseases Week, IDWeek 2019. Washington DC United States. 6(Supplement 2):S980-S981.
Siegmund-Schultze N. 2022. Safety of hepatitis B vaccines: no more heart attacks with CpG adjuvant compared to aluminum hydroxide. Deutsches Arzteblatt international. 119(25):A1150.
Slovin SF, Gardner JP, Morrissey DM, Donovan GP, Surowitz KG, Olson WC, Scher HI, Israel RJ. 2004. Phase I trial of an adjuvanted recombinant soluble prostate-specific membrane antigen (RSPSMA) vaccine. Journal of urology. 171(4):286-287.
Sullivan K, Abhyankar S, Campora L, Cellini C, Chandrasekar P, de la Serna J, El Idrissi M, Gaidano G, Issa N, Lee JJ et al. 2018. Immunogenicity and Safety of an Adjuvanted Herpes Zoster Subunit Vaccine in Adult Autologous Hematopoietic Stem Cell Transplant Recipients: phase 3, Randomized, Placebo-Controlled, ZOE-HSCT Clinical Trial. Bone marrow transplantation. 53:95-97.
Timms LM, Marshall RN, Breslin MF. 1994. Laboratory and field trial assessment of protection given bt a salmonella-enteritidis pt4 inactivated. adjuvant vaccine. British veterinary journal. 150(1):93-102.
Tomaka F, Stieh D, Barouch D, Robb M, Michael N, Tomaras G, Alter G, McElrath J, Lavreys L, Nijs S et al. 2018. Long-term data from APPROACH: phase 1/2a randomized, double-blind, placebo-controlled study evaluating safety/ tolerability and immunogenicity of vaccine regimens using combinations of Ad26. Mos. HIV, MVA-mosaic and gp140 envelope protein. Journal of the international AIDS society Conference: 22nd international AIDS conference, AIDS 2018 Netherlands. 21(Supplement 6) (no pagination).
Tomaka F, Stieh DJ, Barouch DH, Robb ML, Michael NL, Lavreys L, Nijs S, Callewaert K, Hendriks J, Euler Z et al. 2019. Two-year post-vaccination follow-up from APPROACH: phase 1/2a randomized study evaluating safety and immunogenicity of prophylactic HIV vaccine regimens combining Ad26.Mos.HIV and gp140 envelope protein. Journal of the International AIDS Society. 22.
Vajo Z, Balaton G, Vajo P, Torzsa P. 2021. A reduced dose whole virion aluminum adjuvanted seasonal influenza vaccine is immunogenic, safe, and well tolerated in pediatric patients. Viruses. 13(3).
Van Buynder PG, Dhaliwal JK, Van Buynder JL, Couturier C, Minville-Leblanc M, Garceau R, et al. 2010. Protective effect of single-dose adjuvanted pandemic influenza vaccine in children. Influenza and other respiratory viruses. 4(4):171-178.
Vink P, Mingorance ID, Alonso CM. 2020. Immunogenicity and safety of the adjuvanted recombinant zoster vaccine in patients with solid tumors, vaccinated before or during chemotherapy: a randomized trial 9vol 125, pg 1301, 2019). Cancer. 126(12):2941-2941.
Weibel RE, Woodhour AF, Stokes Jr J, Metzgar DP, Hilleman MR. 1967. New metabolizable immunologic adjuvant for human use. 5. Evaluation of highly purified influenza-virus vaccine in adjuvant 65. New England journal of medicine. 276(2):78-84.
Weitz B, Klimek L, Renz H. 2007. Conjugated vaccine as a new therapy option for allergic rhinitis. Laryngo- rhino- otologie. 86(4):248-249.
Wigand R, Esser I, Bodtlander D. 1971. Aluminium hydroxide as a cause of non-specificity in the intracutaneous vaccinia test. Deutsche medizinische Wochenschrift (1946). 96(51):1988-1989.

Woolridge RL, Grayston JT, Chang IH, Yang CY, Cheng KH. 1967. Long-term follow-up of the initial (1959-1960) trachoma vaccine field trial on Taiwan. American journal of ophthalmology. 63(5):Suppl:1650-1655.
Xia S, Duan K, Zhang Y, Zhao D, Zhang H, Xie Z, Li X, Peng C, Zhang W, Yang Y et al. 2020. Effect of an Inactivated Vaccine Against SARS-CoV-2 on Safety and Immunogenicity Outcomes: interim Analysis of 2 Randomized Clinical Trials. JAMA. 324(10):951-960.
Zhang L, Xu ZL, Mak JWY, Chow KM, Lui G, Li TCM, Wong CK, Chan PKS, Ching JYL, Fujiwara Y et al. 2022. Gut microbiota-derived synbiotic formula (SIM01) as a novel adjuvant therapy for COVID-19: an open-label pilot study. Journal of gastroenterology and hepatology. 37(5):823-831.
Zhang NR, Li KC, Liu ZZ, Nandakumar KS, Jiang SB. 2022. A Perspective on the Roles of Adjuvants in Developing Highly Potent COVID-19 Vaccines. VIRUSES-BASEL. 14(2).
Zhang Y, Ma X, Yan G, Wu Y, Chen Y, Zhou Z, Wan N, Su W, Liu FW, Dai MX et al. 2022. Immunogenicity, durability, and safety of an mRNA and three platform-based COVID-19 vaccines as a third dose following two doses of CoronaVac in China: a randomised, double-blinded, placebo-controlled, phase 2 trial. EClinicalMedicine. 54:101680.
Zhang Y, Wang Y, Jia C, Li G, Zhang W, Li Q, Chen X, Leng W, Huang L, Xie Z et al. 2022. Immunogenicity and safety of an egg culture-based quadrivalent inactivated non-adjuvanted subunit influenza vaccine in subjects ≥3 years: a randomized, multicenter, double-blind, active-controlled phase III, non-inferiority trial. Vaccine. 40(34):4933-4941.
